# Repositioning a Human Kinase Platelet-Derived Growth Factor Receptor Alpha Type II Inhibitor for Malaria and Inhibition of Hemozoin Formation

**DOI:** 10.1021/acsmedchemlett.5c00560

**Published:** 2025-12-26

**Authors:** Mmakwena M. Mmonwa, Oluwatosin Audu, Keletso Maepa, Godwin A. Dziwornu, Preshen Govender, Liso Tshaka, James Burrows, Dale Taylor, Keabetswe Masike, Mathew Njoroge, Kathryn J. Wicht, Lauren B. Coulson, Kelly Chibale

**Affiliations:** † Holistic Drug Discovery and Development (H3D) Centre, Department of Chemistry, 37716 University of Cape Town, Rondebosch 7701, South Africa; ‡ Department of Chemistry, KwaDlangezwa Campus, University of Zululand, Empangeni 3886, South Africa; § Department of Chemistry, 37716 University of Cape Town, Rondebosch 7701, South Africa; ∥ Holistic Drug Discovery and Development (H3D) Centre, Institute of Infectious Disease and Molecular Medicine, 37716 University of Cape Town, Observatory, Cape Town 7925, South Africa; ⊥ South African Medical Research Council Drug Discovery and Development Research Unit, Department of Chemistry and Institute of Infectious Disease and Molecular Medicine, 37716 University of Cape Town, Rondebosch 7701, South Africa

**Keywords:** Malaria, Plasmodium, β-Hematin, Hemozoin

## Abstract

A type II Platelet-Derived Growth Factor Receptor Alpha **(**PDGFRA) human kinase inhibitor **GSK190937**, with antiplasmodium activity against asexual blood stage parasites (*Pf*NF54 IC_50_ = 0.22 μM) was identified from the Kinase Chemogenomics Set, a collection of narrow-spectrum human kinase inhibitors. Medicinal chemistry progression of the hit focused on improving potency, selectivity, and ADME properties, leading to compounds **20**, **23**, and **29** with improved microsomal metabolic stability and asexual blood stage antiplasmodium activity. Mechanism of action studies showed that this series inhibits hemozoin formation, killing late-stage trophozoites.

## Graphical abstract

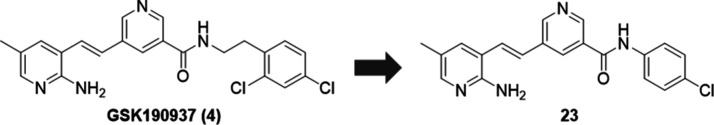

Although preventable and curable, malaria, a disease caused by *Plasmodium* parasites, remains a challenging problem in many parts of the world, particularly in sub-Saharan Africa. According to the World Health Organization (WHO), 263 million cases of malaria and 597 000 deaths were reported globally in 2023.^1^ This is an increase of 14 million cases from 249 million cases in 2022. Of these, 94% of all malaria cases and 95% of deaths in 2023 occurred in the African region. Children under five years of age in the African region accounted for an estimated 76% of all malaria deaths in 2023. The currently approved and recommended antimalarial drugs, particularly artemisinin-based combination therapy (ACT) regimens, are fast becoming less efficacious against the *Plasmodium* parasite due to the emergence of resistance-conferring mutations in the parasite, resulting in a delayed death phenotype.^2,3^ While chemoprophylaxis efforts through the administration of the RTS,S/AS01 malaria vaccine to children under the age of 5 in endemic areas is a major advance toward control and eradication of the disease,^4^ this vaccine only provides modest (∼30%) protection against severe malaria. New affordable drugs with a lower propensity for resistance are needed to overcome the rapid rise in parasite resistance toward first line treatments for malaria.

Human kinase inhibitors with potent antiplasmodium activity have been identified as promising starting points for malaria drug discovery and have led to the identification of several promising *Plasmodium* kinase targets.^5–7^

**GSK190937** (Figure 1), a human kinase Platelet-Derived Growth Factor Receptor Alpha (PDGFRA) type II inhibitor, was identified from a phenotypic screen of the Kinase Chemogenomics Set (KCGS)^8^ against asexual blood stage *Plasmodium falciparum (Pf)* NF54 parasites. **GSK190937** displayed an IC_50_ of 0.22 μM against *Pf*NF54 parasites and no significant cross resistance with the multidrug resistant *Pf*K1 strain, albeit with low solubility and low microsomal metabolic stability.

**Figure 1:**
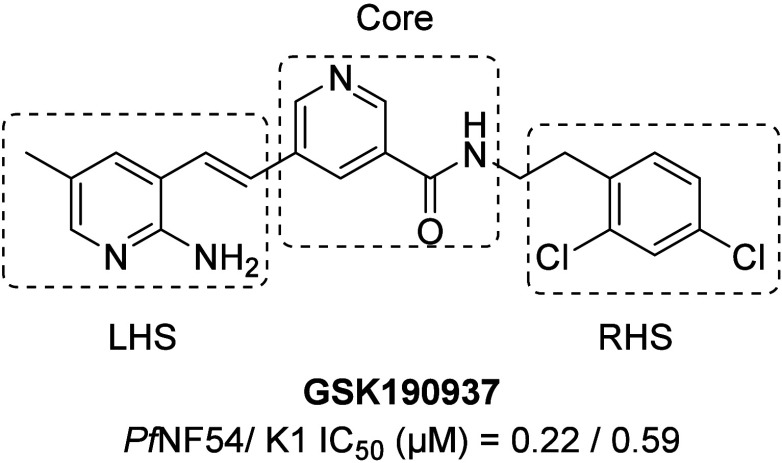
Structure of **GSK190937**. The three regions for SAR exploration are labeled. LHS = left-hand side; RHS = right-hand side.

Here we describe the medicinal chemistry exploration of **GSK190937** with the goal of optimizing potency, selectivity, and ADME properties. This exploration focused on three regions of the molecule, the left-hand side (LHS), the right-hand side (RHS) and the core (Figure 1). We demonstrate that this series inhibits β-hematin formation in a cell-free assay with representative compounds inhibiting hemozoin formation within the parasite, leading to a dose-dependent accumulation of toxic heme.

## Synthesis

We first investigated the structure–activity relationship (SAR) on the LHS of the molecule. Hydrolysis of ethyl 5-bromonicotinate with aqueous NaOH in methanol afforded 5-bromonicotinic acid **1**, which was subjected to amide coupling with 2-(2,4-dichlorophenyl)­ethan-1-amine to deliver 5-bromonicotinamide intermediate **2**. The vinyl group was introduced via a palladium-catalyzed Stille cross-coupling reaction of the latter with tributyl­(vinyl)­stannane in toluene to afford 5-vinylnicotinamide **3**. A palladium-catalyzed Heck cross-coupling reaction of intermediate **3** with various aryl bromides afforded target compounds **4**–**13** (Scheme 1). In addition, compounds **22** and **23**, as well as **26**–**29** were prepared following Scheme 1 using relevant bromo aryl-esters in place of the ethyl 5-bromonicotinate (see Supplementary Information).

**Scheme 1:**
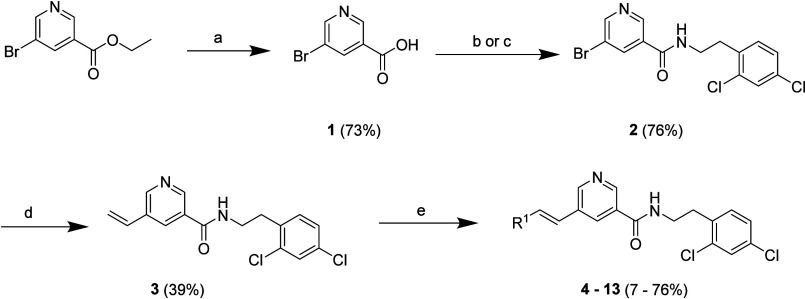
Synthesis of Compounds 4–13

Compounds for RHS SAR exploration were synthesized, as depicted in Scheme 2. A Stille cross-coupling reaction of ethyl 5-bromonicotinate with butyl­(vinyl)­stannane in toluene afforded 5-vinylnicotinamide **14**, which was subjected to a Heck cross-coupling reaction with 3-bromo-5-methylpyridin-2-amine to generate intermediate **15**. This intermediate was hydrolyzed with aqueous NaOH in methanol to deliver the carboxylic derivative **16**, which was subjected to amide coupling with various amines to afford target compounds **17**–**21** and **24**.

**Scheme 2:**
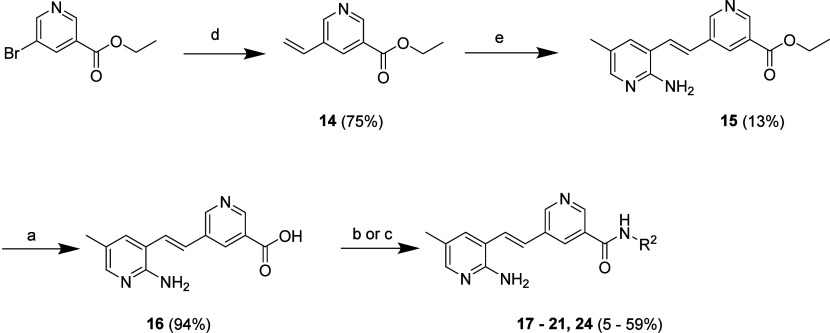
Synthesis of Compounds 17–21, 24

a NaOH­(aq), MeOH, 60 °C, 1 h
b R-NH_2_, HATU, Et_3_N, 1,4-Dioxane, 60 °C, 1 h;
c EDC, HOBt Hydrate, CH_3_CN:THF (1:1, v/v), 25 °C, 1 h;
d tributyl­(vinyl)­stannane, Pd­(PPh_3_)­Cl_2_, Toluene, 120 °C, 0.5 h;
e 3-bromo-5-methylpyridin-2-amine, Pd­(OAc)_2_, PPh_3_, Et_3_N, THF, sealed tube, 140 °C, 3 h.

## Antiplasmodium Activity and Cytotoxicity

The resynthesized hit **GSK190937** and derivatives were evaluated for *in vitro* asexual blood stage (ABS) activity against drug sensitive *Pf*NF54 parasites and cytotoxicity against Chinese hamster ovarian (CHO) cells. Compounds with *Pf*NF54 IC_50_ ≤ 1 μM were further tested against multidrug-resistant *Pf*K1 and/or *Pf*Dd2 strains (Tables 1 and 2).

**Table 1:**
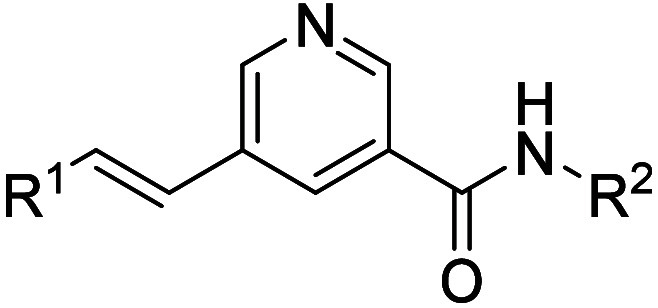

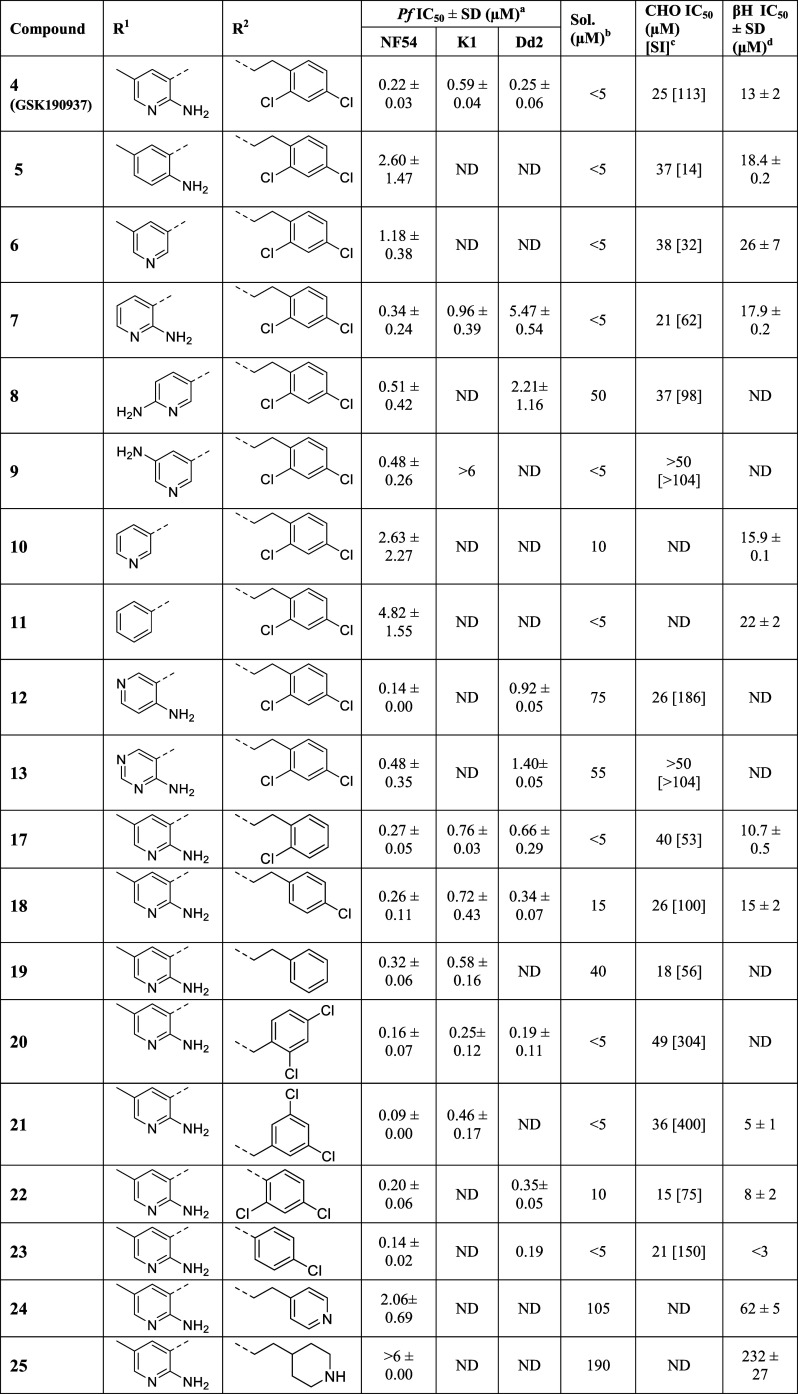
*In Vitro* Asexual Blood Stage Activity Against *P. falciparum* NF54, Dd2 and K1 Strains, Chinese Hamster Ovarian (CHO) Cytotoxicity, Kinetic Aqueous Solubility, and Inhibition of β-Hematin Formation (βH) (LHS SAR)^a^

* ND: Not determined.

a Mean IC_50_ values ± standard deviation (SD) were calculated based on *n* ≥ 2 independent experiments, each with technical duplicates;

b Thermodynamic solubility at pH 6.5;

c Chinese hamster ovary cells tested as one biological replicate with technical triplicates [SI] selectivity index;

d Mean IC_50_ ± SD values were calculated from 2 independent experiments, each with technical duplicates; βH: β-hematin, Chloroquine βH IC_50_ = 20 ± 1 μM.

**Table 2:**
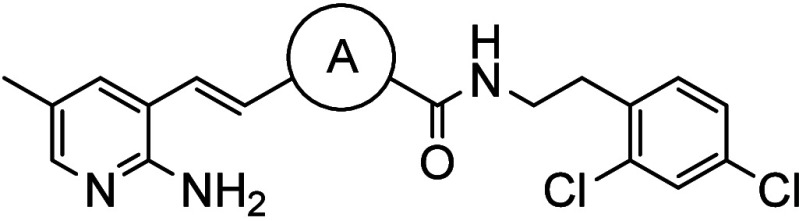

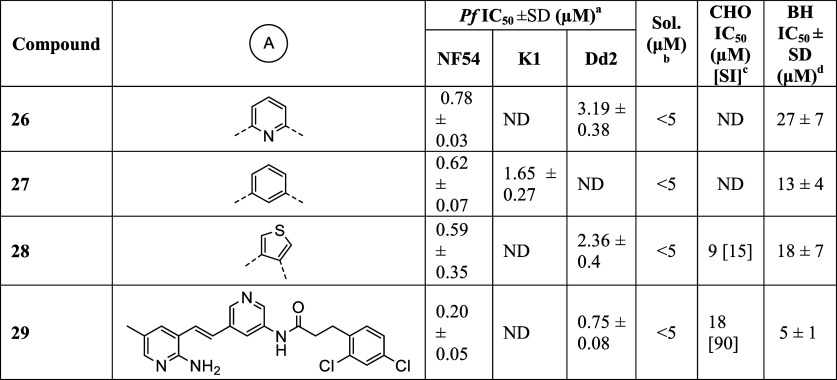
*In Vitro* Asexual Blood Stage *P. falciparum* NF54/Dd2/K1 Parasite Activity, Chinese Hamster Ovarian (CHO) Cytotoxicity, Kinetic Aqueous Solubility, and Inhibition of β-Hematin Formation (βH) IC_50_ Values (Core Change SAR)^a^

* ND: Not determined.

a Mean IC_50_ values ± standard deviation (SD) were calculated based on *n* ≥ 2 independent experiments, each with technical duplicates;

b Thermodynamic solubility at pH 6.5;

c Chinese hamster ovary cells tested as one biological replicate with technical triplicates [SI] selectivity index;

d Mean IC_50_ ± SD values were calculated from 2 independent experiments, each with technical duplicates; βH: β-hematin, Chloroquine βH IC_50_ = 20 ± 1 μM.

### LHS SAR

Resynthesized **GSK190937** displayed ABS activity comparable to that of the original hit from the KCGS library (*Pf*NF54 IC_50_ = 0.22 μM) with a selectivity index (SI, CHO IC_50_/*Pf*NF54 IC_50_) of 113 relative to CHO cells. No significant cross-resistance was observed against the *Pf*K1 and *Pf*Dd2 strains (IC_50_ = 0.59 and 0.25 μM, respectively). Removal of pyridine nitrogen or amino substituent in compounds **5** and **6**, respectively, led to significantly reduced *Pf*NF54 activity (IC_50_ = 2.60 and 1.18 μM, respectively). When the pyridine methyl substituent was removed in compound **7**, the *Pf*NF54 activity was retained (IC_50_ = 0.34 μM). While compound **7** showed marginal cross-resistance against the *Pf*K1 strain (IC_50_ = 0.96 μM), significant cross-resistance was observed against the *Pf*Dd2 strain (IC_50_ = 5.47 μM). Over 2-fold loss in *Pf*NF54 activity (IC_50_ = 0.51 and 0.48 μM) was observed when the amino substituent was moved to position 5 or 6 of the pyridine ring as in compounds **8** and **9**, respectively. These compounds showed selectivity indices comparable to those of the parent compound (SI = 98 and 104). Cross-resistance was also observed for compounds **8** and **9** with the former displaying an IC_50_ of 2.21 μM against *Pf*Dd2, while the latter showed significant cross resistance against *Pf*K1 strain (IC_50_ > 6 μM). Interestingly, *Pf*NF54 activity (IC_50_ = 0.14 μM) was retained when the amino substituent was moved to position 4 of the pyridine ring in compound **12**. Reduced *Pf*NF54 activity (IC_50_ = 2.63 μM) was observed when both the methyl and amino substituents were omitted from pyridine in compound **10**. On the other hand, there was an ∼20-fold loss in *Pf*NF54 activity (IC_50_ = 4.28 μM) for compound **11** where the 2-amino-5-methylpyridin-3-yl was replaced with a phenyl group. Lastly, replacing the 2-amino-5-methylpyridin-3-yl with 4-aminopyrimidin-5-yl in compound **13** resulted in a *Pf*NF54 IC_50_ of 0.48 μM and a SI of 104.

### RHS SAR

Comparable *Pf*NF54 activity to the hit compound was observed when either of the chlorine atoms or both were removed in compounds **17**, **18**, and **19** [IC_50_ (SI) = 0.27 μM (53), 0.26 μM (100) and 0.32 μM (56), respectively] with activity of <1 μM against both *Pf*K1 and *Pf*Dd2 strains. Equipotent activity was also observed when the carbon chain linker was reduced or removed, as in compounds **20** and **22**, respectively (*Pf*NF54/K1/Dd2 IC_50_ = 0.16/0.25/0.19 and 0.20/ND/0.35 μM, respectively). Compound **20** showed improved selectivity (SI = 304) compared to **4** (SI = 113). Shifting the chlorine atoms in compound **20** to positions 3 and 5 as in compound **21** led to comparable *Pf*NF54 and *Pf*K1 activities (*Pf*NF54/K1 IC_50_ = 0.09/0.46 μM) to the hit. Compound **21** also showed high selectivity relative to that of CHO cells (SI = 400). Significant loss of *Pf*NF54 activity was observed when the dichlorophenyl group was replaced with basic groups (4-pyridine and saturated 4-piperidine) as in compounds **24** and **25**. Compound **24** displayed weak *Pf*NF54 activity (IC_50_ = 2.06 μM), while there was a complete loss of activity for **25** at the highest concentration tested (IC_50_ > 6 μM).

### Core Change and Amide Flip SAR

Scaffold hopping led to reduced *Pf*NF54 activity as well as cross resistance against *Pf*K1 and/or *Pf*Dd2 strains relative to resynthesized **4** (Table 2). Moving the amide and vinyl substituents to positions 2 and 6 of the pyridine core, respectively, as in compound **26**, resulted in a 4-fold loss of *Pf*NF54 activity as well as cross resistance against the *Pf*Dd2 strain (*Pf*NF54/Dd2 IC_50_ = 0.78/3.19 μM). Similarly, compound **27** where the pyridine core was replaced with a phenyl group gave *Pf*NF54 IC_50_ = 0.62 μM and showed some cross resistance against the *Pf*K1 strain (IC_50_ = 1.65 μM). Cross-resistance was also observed when a thiophene core was used in place of the pyridine core in compound **28 (** *Pf*NF54/K1 IC_50_ = 0.59 μM/2.36 μM). Flipping the amide group to produce compound **29** resulted in retention of *Pf*NF54 activity (IC_50_ = 0.20 μM) albeit this compound was 4-fold less active against *Pf*Dd2 (IC_50_ = 0.75 μM).

## Mode of Action Studies

As already mentioned, GSK190937 (**4**) is a PDGFRA type II inhibitor.^8^ PDGFRA is part of the receptor tyrosine kinase family, for which there are no *Plasmodium* orthologs.^9^ However, antiplasmodium compounds containing a nicotinamide group have been reported to inhibit hemozoin formation in the parasite via π–π interactions with heme/hemozoin.^10^ Notably, the synthesized compounds **4**–**26** herein contain a nicotinamide core. In addition, the pyridine core in compounds **4**–**26** has the potential to contribute to π-π interactions with heme, while the amide H could contribute to H-bonding, disrupting hemozoin crystal packing.^10^ Based on these hypotheses, selected compounds were tested for their ability to inhibit β-hematin (synthetic hemozoin) formation in a cell free assay (Tables 1 and 2). All tested compounds, except for **25**, showed β-hematin inhibition activity within 5-fold of the control, chloroquine, with IC_50_ < 100 μM. Compounds **21**, **22**, **23**, and **29** showed the most potent inhibition of cell free β-hematin formation, with IC_50_ values at least 2-fold less than chloroquine (IC_50_ < 10 μM).

In the cellular context, antiplasmodium activity mediated by disruption of the hemoglobin degradation pathway is largely dependent on the extent to which compounds accumulate in the acidic digestive vacuole via heme binding and pH trapping.^11,12^ To confirm that inhibition of hemozoin is contributing to antiplasmodium activity in the cellular context, intracellular heme fractionation assays were carried out.^13,14^

In the cell fractionation assay, a compound that inhibits the formation of intracellular hemozoin in the digestive vacuole of the parasite causes an increase in the absolute amount (fg/cell) of free heme and a decrease in the amount of hemozoin relative to the untreated control. Single-point cell fractionation was performed with compounds **4**(**GSK190937**) and **17** at 2× their IC_50_ values using chloroquine and mefloquine as positive and negative controls, respectively (Figure 2A). Both tested compounds showed significantly increased levels of absolute free heme at 2 × IC_50_, even more so than the classical hemozoin formation inhibitor chloroquine, relative to the untreated control parasites, with an equally significant decrease in the hemozoin levels (Figure 2B). These high levels may be indicative of the compounds killing the parasites later in the trophozoite stage compared to chloroquine, allowing more time for the buildup of heme. Furthermore, compound **4** was tested using this assay at increasing concentrations from 0.5× to 3× IC_50_, resulting in a dose-dependent increase in free heme and decrease in hemozoin (Figure 2C and 2D). Additionally, an inverse correlation was observed between parasite survival and the amount of free heme, where the curves intersected close to the IC_50_ value of compound **4**. This strongly suggests that elevated levels of cytotoxic heme cause inhibition of parasite growth and, therefore, that inhibition of hemozoin formation is the primary mode of action for this series.

**Figure 2:**
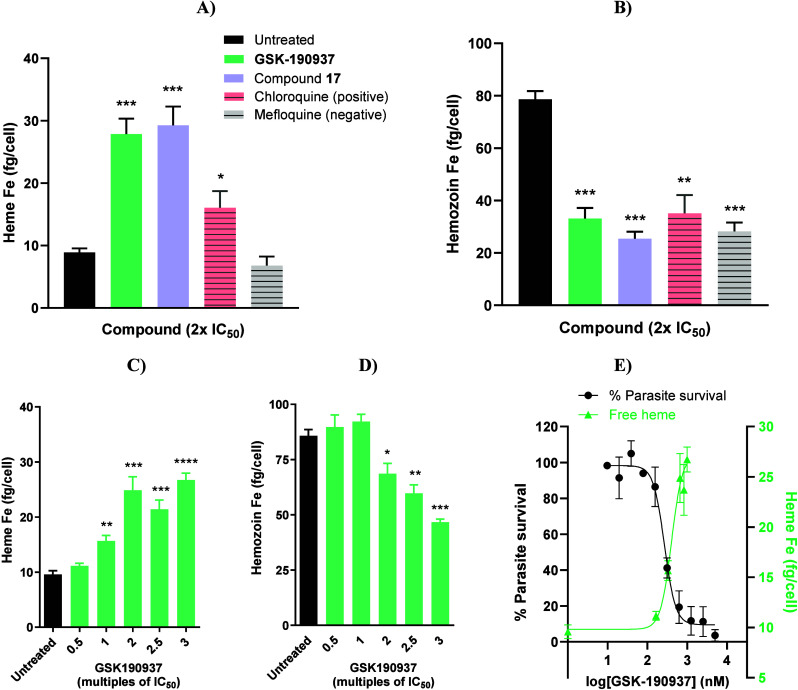
Cell fractionation results showing A) the increase in free heme levels (fg of heme-derived Fe per cell) and **B**) hemozoin levels (fg of hemozoin-derived Fe per cell) at 2× IC_50_ (single point) for **GSK190937** (**4**) and compound **17** relative to controls, chloroquine and mefloquine. Bars represent mean ± SEM from two independent experiments where *n* = 4. **C**) The dose–response increase in free heme or **D**) dose–response decrease in hemozoin for **GSK190937** (**4**) relative to the untreated control (black). Bars represent mean ± SEM from two independent experiments where *n* = 4. Significance was determined using unpaired *t*-tests where comparisons are shown relative to the untreated control; *p < 0.05; **p < 0.01; ***p < 0.001; ****p < 0.0001. **E**) The inverse correlation between parasite survival and levels of free heme for **GSK190937** (**4**). The curves intersect close to the IC_50_ value, suggesting that increased heme levels are driving parasite cell death. Values represent mean ± SEM from *n* = 4.

## Aqueous Solubility and Microsomal Metabolic Stability

A good oral drug candidate should meet acceptable levels of solubility and microsomal stability, among other parameters.^15^ With this in mind, emphasis was also put on improving these parameters for the prepared derivatives (Tables 1 and 2). Using the kinetic solubility assay at pH 6.5, improved solubility relative to the hit compound (solubility <5 μM) was observed for compounds **8**, **12**, **13**, **18**, **19**, **24**, and **25**. Shifting the LHS amino substituent to position 6 or 4 of the pyridine in compounds **8** and **12** improved the solubility to 50 and 75 μM, respectively, while the amino pyrimidinyl group in compound **13** had a solubility of 55 μM. On the other hand, the removal of 2-chloro from the RHS phenyl group resulted in a small improvement in solubility to 15 μM, while removing both chlorine atoms in compound **19** resulted in a solubility of 40 μM. Replacing the dichlorophenyl group on the RHS with hydrophilic groups, pyridine and piperidine groups, in compounds **24** and **25** significantly improved the solubility to 105 and 190 μM, respectively, albeit reduced *Pf*NF54 and cell-free β-hematin formation inhibition activity.

Lastly, due to low microsomal metabolic stability of the hit compound **4**, we tested selected compounds for metabolic stability using mouse, rat and human liver microsomes (Table 3). Despite most of these compounds showing low microsomal stability, compounds **20** and **23** with a reduced or removed RHS alkyl chain linker, respectively, as well as compound **29** with a flipped amide group showed improved microsomal stability across all three species.

**Table 3:**
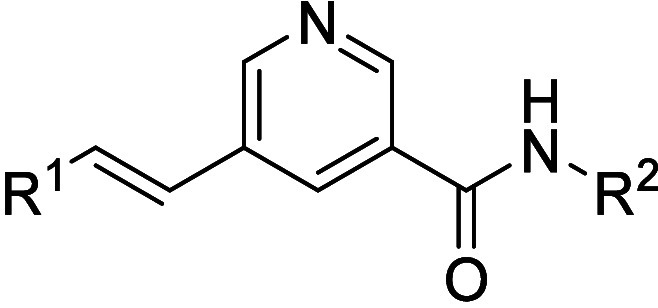

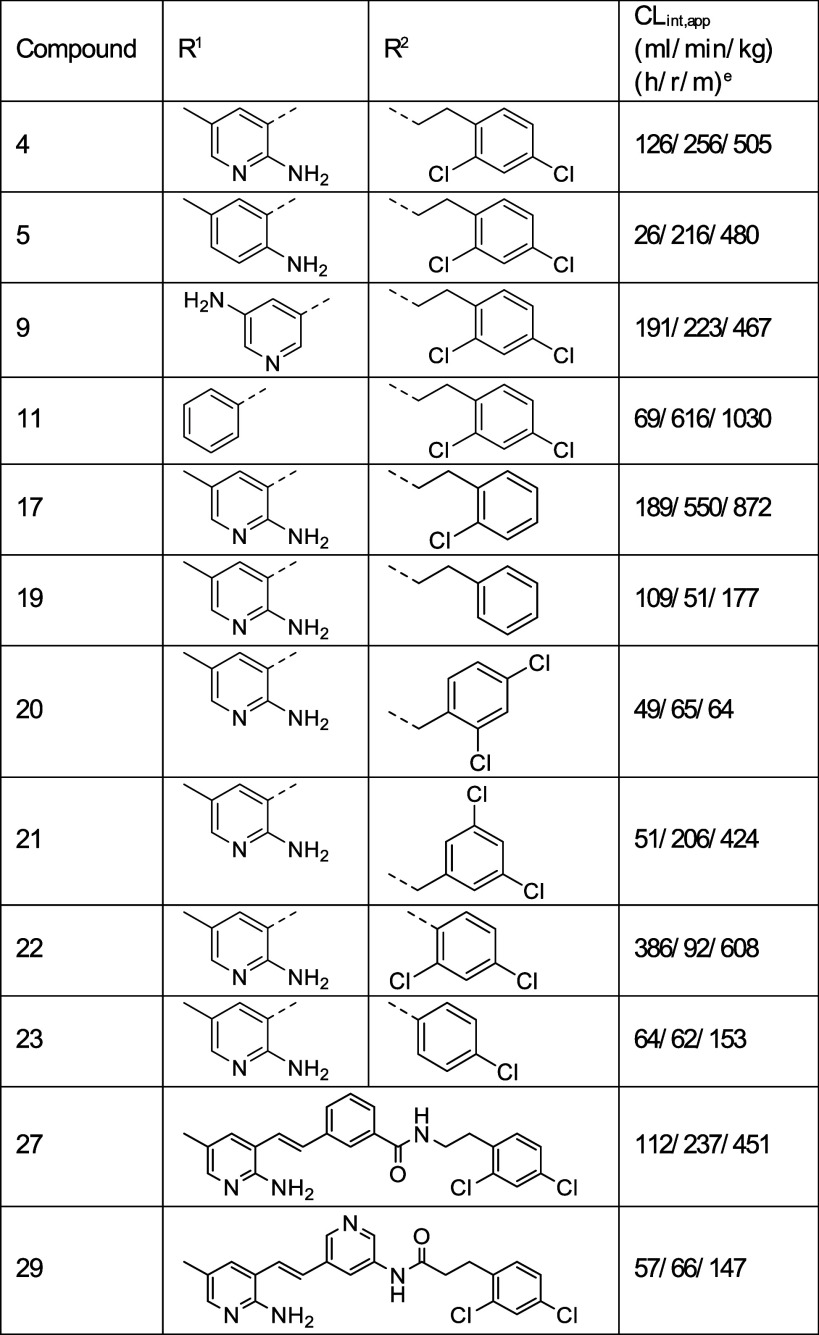
*In Vitro* Microsomal Stability for Selected Compounds

1 Predicted intrinsic clearance using human, rat and mouse liver microsomes.

**GSK190937** (**4**), identified from the Kinase Chemogenomics Set, showed antiplasmodium activity against both sensitive *Pf*NF54 and multidrug resistant *Pf*K1 strains. The medicinal chemistry efforts to address the low aqueous solubility and microsomal metabolic stability led to identification of compounds **20**, **23**, and **29** with equipotent antiplasmodium activities and improved microsomal stability. Reduced or lost antiplasmodium activity was observed with solubility improvement. Mode of action studies revealed that this series inhibits hemozoin formation with significantly elevated levels of free heme at concentrations ≥ IC_50_. We conducted a formal hit assessment to evaluate if the series has a tractable SAR and early ADME studies toward identifying issues to be addressed in a future lead optimization campaign, including addressing solubility, which remains an issue for the series. Work to further improve the antiplasmodium activity and physicochemical properties toward a pharmacology proof-of-concept in a malaria mouse infection model is ongoing in our laboratories.

## Supplementary Material



## Notes

Experimental procedures of some intermediates and all target compounds. The intermediates and target compounds were characterized using ^1^H and/or ^13^C NMR, and purity was determined using LC-MS. Copies of LC-MS and ^1^H NMR spectra for all target compounds are included. ^13^C NMR spectra of lead compounds are also included. Biological and physicochemical assays procedures are provided as well. (PDF)

## Notes

No unexpected or unusually high safety hazards were encountered.

## Abbreviations

PDGFRA: Platelet-Derived Growth Factor Receptor Alpha
ADME: Absorption, Distribution, Metabolism and Excretion
WHO: World Health Organization
KCGS: Kinase Chemogenomics Set
NaOH: Sodium hydroxide
HATU: 1-[Bis­(dimethylamino)­methylene]-1*H*-1,2,3-triazolo­[4,5-*b*]-pyridinium 3-oxid hexafluorophosphate
EDC: 1-Ethyl-3-(3-(dimethylamino)­propyl)­carbodiimide
HOBt: Hydroxybenzotriazole
CH_3_CN: Acetonitrile
THF: Tetrahydrofuran
Pd­(PPh_3_)­Cl_2_: Bis­(triphenylphosphine)­palladium­(II) chloride
PPh_3_: Triphenylphosphine
Pd­(OAC)_2_: Palladium­(II) acetate
Et_3_N: Triethylamine
SAR: Structure Activity Relationship
SI: Selectivity Index
RHS: Right Hand Side
LHS: Left Hand Side:
CHO: Chinese Hamster Ovary
βH: Beta-hematin
LCMS: Liquid Chromatography–Mass Spectrometry
NMR: Nuclear Magnetic Resonance
DMSO: Dimethyl sulfoxide
pLDH: Plasmodium lactate dehydrogenase
