# Supplementary material for: Repositioning a Human Kinase Platelet-Derived Growth Factor Receptor Alpha Type II Inhibitor for Malaria and Inhibition of Hemozoin Formation
Source: ACS Med Chem Lett. 2025 Dec 26;17(1):162–9. doi: 10.1021/acsmedchemlett.5c00560 (PMC12794062; doi:10.1021/acsmedchemlett.5c00560)
Supplement: Supplementary file 1 [file ml5c00560_si_001.pdf]

Supporting Information

Repositioning a human kinase Platelet-Derived Growth Factor Receptor Alpha type II inhibitor for malaria and inhibition of hemozoin formation

Mmakwena M. Mmonwa,[a,b‡] *Oluwatosin Audu,*[a‡] *Keletso Maepa,*[c] *Godwin A. Dziwornu,*[a] *Preshen Govender,*[a] *Liso Tshaka,*[a] *James Burrows,*[a] *Dale Taylor,*[d] *Keabetswe Masike,*[d] *Mathew Njoroge,*[d] *Kathryn J. Wicht,*[a,e] *Lauren B. Coulson*[d*] *and Kelly Chibale,*[a,e*]

[a]Holistic Drug Discovery and Development (H3D) Centre, Department of Chemistry, University of Cape Town, Rondebosch 7701, South Africa;
[b]Department of Chemistry, KwaDlangezwa Campus, University of Zululand, Empangeni 3886, South Africa
[c]Department of Chemistry, University of Cape Town, Rondebosch 7701, South Africa;
[d]Holistic Drug Discovery and Development (H3D) Centre, Institute of Infectious Disease and Molecular Medicine, University of Cape Town, Observatory, Cape Town 7925, South Africa;
[e]South African Medical Research Council Drug Discovery and Development Research Unit, Department of Chemistry and Institute of Infectious Disease and Molecular Medicine, University of Cape Town, Rondebosch 7701, South Africa.
South Africa
‡These authors contributed equally to this work.
*Corresponding authors: Kelly Chibale (kelly.chibale@uct.ac.za); Lauren B. Coulson (lauren.coulson@uct.ac.za)

TABLE OF CONTENTS

1. Experimental section S2
2. Analytical data of final compounds S14
3. Biological and physicochemical assays S61
4. References S64

1. Experimental Section

All commercially available chemicals were purchased from Sigma-Aldrich, Merck, or Combi-Blocks Limited and were of analytical grade, thus generally used without further purification. Reactions were monitored via analytical thin layer chromatography (TLC) sourced from Merck (TLC Silica gel 60 F_{254} aluminium-backed) and LCMS. The former was visualized under ultraviolet light at 254 nm. Where necessary, compounds were purified on silica gel chromatography using flash column chromatography on a Biotage Isolera™ system (Biotage AB, Uppsala, Sweden). ^{1}H NMR spectra were recorded on Varian Mercury (300 MHz). ^{13}C NMR spectra were recorded on the Bruker Ultrashield-Plus (101 MHz), or a Bruker (151 MHz). NMR samples were dissolved in deuterated dimethyl sulfoxide (DMSO-d_6), or chloroform ($CDCl_3$). Chemical shifts (δ) are reported in parts per million (ppm) and rounded to two decimal places. Coupling constants (*J*) are reported in Hertz (Hz) and rounded to two decimal places. Abbreviations used in assigning ^{1}H-NMR signals are d (doublet), dd (doublet of doublets), ddd (doublet of doublet of doublets), m (multiplet), q (quartet), s (singlet), t (triplet), or td (triplet of doublets).

Mass spectra used to determine the purities of target compounds were acquired on an Agilent HPLC system equipped with Agilent 1260® Infinity Binary Pump, Agilent 1260® Infinity Diode Array Detector, Agilent 1290® Infinity Column Compartment, Agilent 1260® Infinity Autosampler, Agilent 6120® Quadrupole LCMS, and Peak Scientific® Genius 1050 Nitrogen Generator. The column used was an X-bridge® C18, 2.6 μm, 30 mm (ID) 2C.1 mm (length) maintained at 35 ˚C. The composition and gradient conditions of the mobile phase used at a flow rate of 0.9 mL/min are listed in Table 4. The injection volume was 2 μL, and the mass spectra were obtained in the positive mode by electrospray ionization (ESI) and atmospheric pressure chemical ionization (APCI). The diode array detector was programmed to scan the eluents at an absorption wavelength range of 210-640 nm. All synthesized intermediates were characterized by LCMS, while final compounds were confirmed by LCMS and, at least, a ^{1}H NMR data. The purity of target compounds was determined by HPLC, and all compounds were confirmed to have > 95% purity.

General synthesis methods

Method A: Intermediate **3** (1.00 equiv), bromo pyridine/aniline derivatives (3.00 equiv), $Pd(OAc)_2$ (0.20 equiv), PPh_3 (0.40 equiv), Et_3N (2.00 equiv) were placed in a round sealed tube with a stirrer bar. 1, 4-Dioxane (3 mL) or Dry tetrahydrofuran (THF) (3 mL) was added, and the mixture was degassed by bubbling N_2 gas through the mixture, at room temperature for 10 min. A balloon filled with nitrogen gas was connected to the top of the condenser and the mixture was stirred at 110 ^{0}C for 3 h or until the reaction was complete (tlc and LCMS monitored). After the completion of the reaction, the mixture was cooled to ambient temperature, quenched with ice-cold water (5 mL). The crude product was dissolved in MeOH and filtered through celite. Purification for all target compounds were done by normal phase Teledyne ISCO CombiFlash using decreasing polar mixtures of 0.5 M NH_3/MeOH in DCM as eluents, and reverse phase Teledyne ISCO CombiFlash or biotage column chromatography

(C18 OBD column, 5 μm silica 19 mm diameter, 150 mm length) using decreasingly polar mixtures of milli-q water and MeCN as eluents. Fractions containing the desired product were evaporated under reduced pressure to dryness to afford target compounds.

Method B: (*E*)-5-(2-(2-amino-5-methylpyridin-3-yl)vinyl)nicotinic acid **16** (1.00 equiv), relevant amine (1.50 equiv), HATU (2.50 equiv), Et_3N (2.50 equiv) were placed in a round bottom flask equipped with a stirrer bar. Dry 1,4-dioxane (10 mL) was added, and the mixture was stirred at 60 ^{0}C for 1 h or until the reaction was complete (tlc and LCMS monitored). After the completion of the reaction, solvent was removed under reduced pressure, the crude product was purified by either by normal phase using decreasingly mixtures of 0.5 M NH_3/MeOH in DCM as eluents or reverse phase biotage column chromatography (C18 OBD column, 5 μm silica 19 mm diameter, 150 mm length), using decreasingly polar mixtures of milli-q water and MeCN as eluents. Fractions containing the desired product were evaporated under reduced pressure to 20 mL and freeze-dried dryness to give target compounds.

Synthesis of intermediates

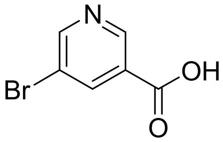

5-Bromonicotinic acid (1)

To a stirring solution of ethyl 5-bromonicotinic acid ethyl ester (200.0 mg, 0.87 mmol) in methanol (10 mL) was added 1 M solution of sodium hydroxide (208.64 mg, 5.22 mmol) in water (5 mL). The mixture was stirred at 70 ˚C for 1 h (LC-MS monitored). After the completion of the reaction, the mixture was cooled to an ambient temperature. Solvent was reduced under on rotary evaporator to about 5 mL. The pH of the solution was adjusted to 3 using 2 M aqueous HCl. The precipitate obtained was collected by filtration, dried to afford 5-bromopyridine-3-carboxylic acid (130.0 mg, 0.63 mmol, 72.55 %) as white powder which was used in the next step without further purification. LC-MS: t_R = O.599 min (Purity = 100 %); m/z = 202.0 $[M+H]^+$ (anal. calcd. for $C_6H_4BrNO_2$: m/z = 202.0).

*Synthesis of intermediates **2** and **3***

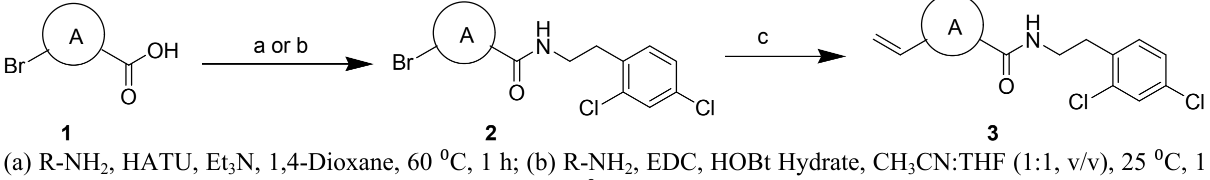

(a) R-NH_2, HATU, Et_3N, 1,4-Dioxane, 60 ^{0}C, 1 h; (b) R-NH_2, EDC, HOBt Hydrate, CH_3CN:THF (1:1, v/v), 25 ^{0}C, 1 h; (c) tributyl(vinyl)stannane, $Pd(PPh_3)Cl_2$, Toluene, 120 ^{0}C, 0.5 h

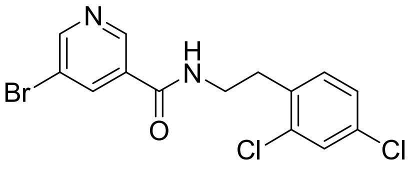

5-Bromo-*N*-(2,4-dichlorophenethyl)nicotinamide (2a)

Intermediate **1** (500 mg, 2.5 mmol), 2-(2,4-dichlorophenyl)ethanamine (705 mg, 3.71 mmol), HATU (2352 mg, 6.19 mmol) and triethylamine (0.86 mL, 6.19 mmol) were placed in a round bottom flask equipped with a stirrer bar and a condenser. 1,4-Dioxane (15 mL) was added to the mixture, and the reacting mixture was stirred at 60 °C for 1 h (TLC and LC-MS monitored). After the completion of the reaction, the mixture was cooled to ambient temperature. Solvent was removed and the crude product obtained was taken up into DCM (20 mL), washed with water (2 x 10 mL). The organic phase was dried with anhydrous $MgSO_4$, the salt was then filtered off and the dried organic solvent was removed under reduced pressure. The crude product was purified by normal phase Biotage column chromatography using increasingly polar mixtures petroleum ether and ethyl acetate as eluents. Test tubes containing desired compound were combined and the solvent evaporated under reduced pressure to give compound **3,** 5-bromo-*N*-[2-(2,4-dichlorophenyl)ethyl]pyridine-3-carboxamide (703 mg, 1.88 mmol, 75.93%) as a white powder which was used without further purification. LC-MS: t_R = 1.097 min (Purity = 100 %); *m/z* = 375.0 $[M+H]^+$ (anal. calcd. for $C_{14}H_{11}BrCl_2N_2O$: *m/z* = 374.0).

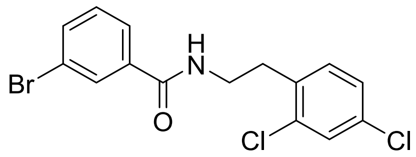

3-Bromo-*N*-[2-(2,4-dichlorophenyl)ethyl]benzamide (2b)

To a solution of 2,4-dichlorophenethylamine (1418.29 mg, 7.46 mmol), *N*-(3-dimethylaminopropyl)-*N*-ethylcarbodiimide hydrochloride (1430.45 mg, 7.46 mmol) and 1-hydroxybenzotriazole hydrate (228.54 mg, 1.49 mmol) in THF (25 mL):acetonitrile (25 mL) was added 3-bromobenzoic acid (1000 mg, 4.97 mmol) in portions at room temperature. The reaction was stirred at 25 °C for 1 h. LC-MS indicated the consumption of the starting material and formation of the desired product. The reaction was stopped and the solvent removed under reduced pressure and the solvent was reduced to 5 mL. Ice-cold water (30 mL) was added into the mixture and the precipitate formed was collected by filtration, dried to give 3-bromo-*N*-[2-(2,4-dichlorophenyl)ethyl]benzamide (1570 mg, 4.21 mmol, 84.6 %) as a white solid which was used in the next step without further purification. LC-MS: t_R = 1.189 min (purity 100%); *m/z* = 373.9 $[M+H]^+$ (anal. calcd. for $C_{15}H_{12}BrCl_2NO$: *m/z* = 372.9).

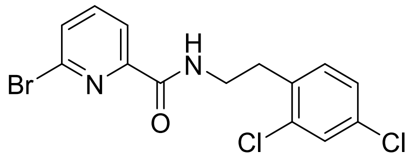

6-Bromo-*N*-[2-(2,4-dichlorophenyl)ethyl]pyridine-2-carboxamide (2c)

A mixture of 6-bromopyridine-2-carboxylic acid (500 mg, 2.48 mmol), 2-(2,4-dichlorophenyl)ethanamine (705.67 mg, 3.71 mmol) , 1-[bis(dimethylamino)methylene]-1*H*-1,2,3-triazolo[4,5-b]pyridinium 3-oxid hexafluorophosphate (2352.85 mg, 6.19 mmol) and triethylamine (0.86mL, 6.19 mmol) was placed in a round bottom flask with a stirrer bar. 1,4-Dioxane (20 mL) was added to the mixture and the solution was stirred at 60 °C for 1 h (LC-MS monitored). After the completion of the reaction, the mixture was cooled to ambient temperature. Solvent was removed and the crude product obtained was taken up into DCM (40 mL), washed with water (2 x 10 mL) to remove the HATU byproduct. The organic phase was dried with anhydrous MgSO4, the salt was then filtered off and the dried organic solvent was removed under reduced pressure to give 6-bromo-*N*-[2-(2,4-dichlorophenyl)ethyl]pyridine-2-carboxamide (772 mg, 1.82 mmol, 73.38 %) as a yellow solid which was used without further purification. LC-MS: t_R = 1.204 min (purity 88%); *m/z* = 374.7 $[M+H]^+$ (anal. calcd. for $C_{14}H_{11}BrCl_2N_2O$: *m/z* = 373.9).

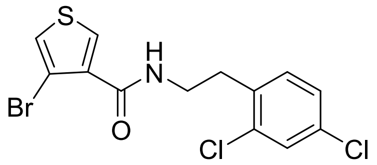

4-Bromo-*N*-[2-(2,4-dichlorophenyl)ethyl]thiophene-3-carboxamide (2d)

A mixture of 4-bromothiophene-3-carboxylic acid (500 mg, 2.41 mmol) , 2-(2,4-dichlorophenyl)ethanamine (688 53mg, 3.62 mmol) , 1-[bis(dimethylamino)methylene]-1*H*-1,2,3-triazolo[4,5-b]pyridinium 3-oxid hexafluorophosphate (2295.69 mg, 6.04 mmol) and triethylamine (0.84 mL, 6.04 mmol) was placed in a round bottom flask with a stirrer bar. 1,4-Dioxane (15 mL) was added to the mixture and the solution was stirred at 60 °C for 1 h (LC-MS monitored). After the completion of the reaction, the mixture was cooled to ambient temperature. Solvent was removed and the crude product was taken up into the DCM (30 mL), washed with deionised water (2 x 10 mL) to remove the HATU byproduct. The DCM layer was dried with anhydrous $MgSO_4$ and the $MgSO_4$ was filtered off. The DCM was removed under reduced pressure to afford 4-bromo-*N*-[2-(2,4-dichlorophenyl)ethyl]thiophene-3-carboxamide (812 mg, 2.01 mmol, 83.37 %) as a brown oil which was used without further purification. LC-MS t_R = 1.134 min (purity 94%); *m/z* = 377.9 $[M+H]^+$ (anal. calcd. for $C_{13}H_{10}BrCl_2NOS$: *m/z* = 376.9).

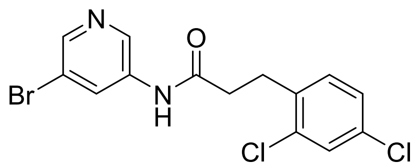

***N*-(5-bromo-3-pyridinyl)-2-(2,4-dichlorophenyl)acetamide (2e)**

Following a method for synthesis of compounds **2a**, a mixture of 3-amino-5-bromopyridine (500 mg, 2.89 mmol), 3-(2,4-dichlorophenyl)propanoic acid (949.63 mg, 4.34 mmol), 1-[bis(dimethylamino)methylene]-1*H*-1,2,3-triazolo[4,5-b]pyridinium 3-oxid hexafluorophosphate (2747.24 mg, 7.23 mmol) and triethylamine (1.01 mL, 7.23 mmol) was placed in a round bottom flask with a stirrer bar. 1,4-Dioxane (20 mL) was added to the mixture and the solution was stirred at 60 °C for 1 h (LC-MS monitored). After the completion of the reaction, the mixture was cooled to ambient temperature. Solvent was removed and the

crude product was dissolved in DCM (20 mL), washed with distilled water (2 x 10 mL). The organic layer was dried with anhydrous $MgSO_4$, filtered and volatiles were removed under reduced pressure to afford *N*-(5-bromo-3-pyridinyl)-3-(2,4-dichlorophenyl)propanamide (980 mg, 1.91 mmol, 66.18 %) as a white gum which was used without further purification. LC-MS: t_R = 1.146 min (Purity = 73 %); *m/z* = 373.0 $[M+H]^+$ (anal. calcd. For $C_{14}H_{11}Br\ Cl_2N_2O$: *m/z* = 372.9).

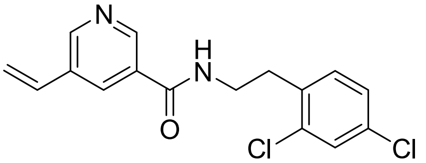

2-(2,4-Dichlorophenyl)-*N*-(5-ethenyl-3-pyridinyl)acetamide (3a)

To a round bottom flask equipped with a stirrer bar, was added compound **2a** (300 mg, 0.8 mmol) and tributyl(ethenyl)stannane (381.48 mg, 1.2 mmol). Toluene (20 mL) was added into the flask and the mixture was degassed with N_2 for 10 minutes, then bis(triphenylphosphine)palladium(II) dichloride (84.44 mg, 0.12 mmol) was added. A condenser equipped with a balloon filled with N_2 gas was connected on top of the round bottom flask. The reaction mixture was stirred at 120 ˚C for 1 h (LC-MS monitored). After the completion of the reaction, the mixture was cooled to ambient temperature. The solvent was removed under reduced pressure. The crude product obtained was taken up in DCM (25 mL), washed with Brine (2 x 5 mL). The organic layer was then dried over anhydrous $MgSO_4$, and the salt was filtered off. The filtrate was then passed through celite, and the solvent was removed under reduced pressure. The crude product was purified by normal phase biotage column chromatography using increasingly polar mixtures petroleum ether and ethyl acetate as eluents. Fractions containing desired compound were combined and the solvent evaporated under reduced pressure to give *N*-[2-(2,4-dichlorophenyl)ethyl]-5-ethenylpyridine-3-carboxamide (181 mg, 0.5522 mmol, 68.86 %) as a white solid which was used without further purification. ^1H NMR (300 MHz, DMSO-d_6) δ_H 8.86 – 8.75 (m, 3H), 8.25 (s, 1H), 7.59 (s, 1H), 7.41 – 7.35 (m, 2H), 6.84 (dd, *J* = 17.7, 11.0 Hz, 1H), 6.05 (d, *J* = 17.7 Hz, 1H), 5.49 (d, *J* = 10.9 Hz, 1H), 3.54 (q, *J* = 6.7 Hz, 2H), 2.98 (t, *J* = 7.1 Hz, 2H). LC-MS: t_R = 1.061 min (Purity = 98 %); *m/z* = 321.0 $[M+H]^+$ (anal. calcd. for $C_{16}H_{14}Cl_2N_2O$: *m/z* = 321.1).

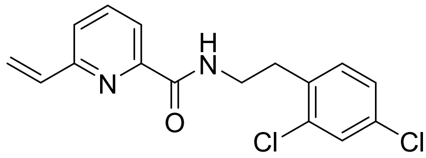

***N*-[2-(2,4-Dichlorophenyl)ethyl]-6-ethenylpyridine-2-carboxamide (3c)**

To a round bottom flask equipped with a stirrer bar, was added **2c** (300 mg, 0.8 mmol) and tributyl(ethenyl)stannane (381 48mg, 1.2 mmol). Toluene (10 mL) was added into the flask and the mixture was degassed with N_2 for 10 minutes, then bis(triphenylphosphine)palladium (II) dichloride (28.15 mg, 0.04 mmol) was added. A condenser equipped with a balloon filled with N_2 was connected on top of the round bottom flask. The reaction mixture was stirred at 120 ˚C for 1 h (LC-MS monitored). After the

completion of the reaction, the mixture was cooled to ambient temperature. The solvent was removed under reduced pressure. The crude product obtained was taken up in DCM (25 mL), washed with Brine (2 x 5 mL). The organic layer was then dried over anhydrous MgSO4, and the salt was filtered off. The filtrate was then passed through celite, and the solvent was removed under reduced pressure to give *N*-[2-(2,4-dichlorophenyl)ethyl]-6-ethenylpyridine-2-carboxamide (201 mg, 0.6258 mmol, 78.03 %) as a dark brown oil which was used without further purification. LC-MS: t_R = 1.205 min (purity 95%); *m/z* = 321.1 $[M+H]^+$ (anal. calcd. for $C_{18}H_{20}N_2O$: *m/z* = 320.0).

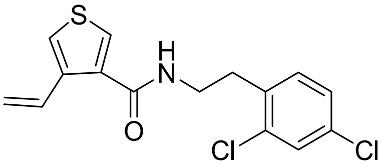

N-(2,4-Dichlorophenethyl)-5-vinylthiophene-3-carboxamide (3d)

To a round bottom flask equipped with a stirrer bar, was added **2d** (700 mg, 2.07 mmol) and tributyl(ethenyl)stannane (984.32 mg, 3.1 mmol). Toluene (20 mL) was added into the flask and the mixture was degassed with N_2 gas for 10 minutes, then bis(triphenylphosphine)palladium(II) dichloride (72.63 mg, 0.10 mmol) was added. A condenser equipped with a balloon filled with N_2 gas was connected on top of the round bottom flask. The reaction mixture was stirred at 120 ˚C for 1 h (LC-MS monitored). After the completion of the reaction, the mixture was cooled to ambient temperature. The solvent was removed under reduced pressure. The crude product obtained was taken up in DCM (25 mL), washed with Brine (2 x 5 mL). The organic layer was then dried over anhydrous $MgSO_{4,}$ and the salt was filtered off. The filtrate was then passed through celite, and the solvent was removed under reduced pressure to give *N*-(2,4-dichlorophenethyl)-5-vinylthiophene-3-carboxamide (390 mg, 1.34 mmol, 64.71 %) as a dark brown oil which was used without further purification. LC-MS t_R = 1.154 min (purity 98%); *m/z* = 326.0 $[M+H]^+$ (anal. calcd. for $C_{15}H_{13}Cl_2NOS$: *m/z* = 325.0).

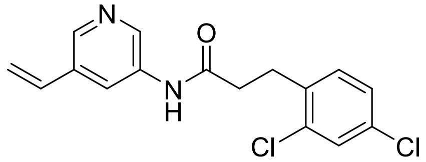

3-(2,4-Dichlorophenyl)-N-(5-ethenyl-3-pyridinyl)propenamide (3e)

Following a method for synthesis of compounds **3a**, a round bottom flask equipped with a stirrer bar, was added **2e** (300 mg, 0.8 mmol) and tributyl(ethenyl)stannane (381.48 mg, 1.2 mmol). Toluene (10 mL) was added into the flask and the mixture was degassed with N_2 gas for 10 minutes, then bis(triphenylphosphine)palladium (II) dichloride (28.15 mg, 0.04 mmol) was added. A condenser equipped with a balloon filled with N_2 was connected on top of the round bottom flask. The reaction mixture was stirred at 120 ˚C for 1 h (LC-MS monitored). After the completion of the reaction, the mixture was cooled to ambient temperature. The solvent was removed under reduced pressure. The crude product obtained was taken up in DCM (25 mL), washed with Brine (2 x 5 mL). The organic layer was then dried over anhydrous $MgSO_{4,}$ and the salt was filtered off. The filtrate was then passed through celite, and the solvent

was removed under reduced pressure to give 3-(2,4-dichlorophenyl)-*N*-(5-ethenyl-3-pyridinyl)propanamide (290 mg, 0.7765 mmol, 96.81%) as a light brown solid which was used without further purification. LC-MS t_R = 0.979 min (purity 86%); *m/z* = 321.1 $[M+H]^+$ (anal. calcd. for $C_{16}H_{14}C_{12}N_2O$: *m/z* = 320.0).

Synthesis of intermediates ***14-16***

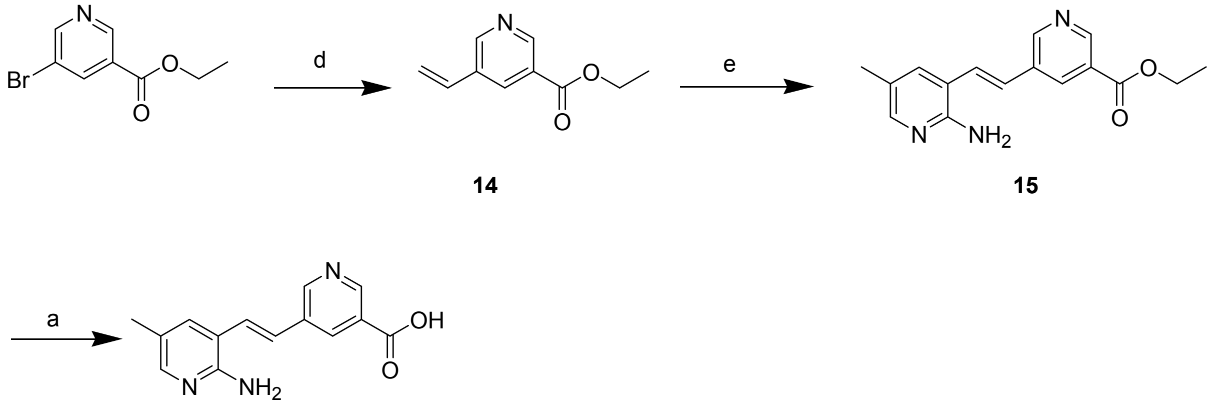

14 **15** **16**

(a) NaOH(aq), MeOH, 60 ^{0}C, 1 h;a (d) tributyl(vinyl)stannane, $Pd(PPh_3)Cl_2$, Toluene, 120 ^{0}C, 0.5 h; (e) 3-bromo-5-methylpyridin-2-amine, $Pd(OAc)_2$, PPh_3, Et_3N, THF, sealed tube, 140 ^{0}C, 3 h.

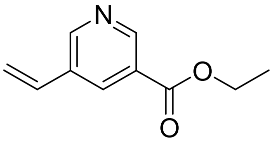

Ethyl 5-vinylnicotinate (14)

To a 100 mL round bottom flask equipped with a stirrer bar, were added ethyl 5-bromopyridine-3-carboxylate (4500 mg, 19.56 mmol), and tributyl(ethenyl)stannane (11. 96 mL, 29.3 mmol). Toluene (25 mL) was added into the flask, and the mixture was degassed with N_2 for 10 minutes, then Bis(triphenylphosphine)palladium(II) dichloride (2750 mg, 3.9 mmol) was added. The reaction was conducted under a dry condition via a balloon filled with N_2 connected on top of the round bottom flask. The reaction mixture was stirred at 120 ˚C for 1 h (LC-MS monitored). After the completion of the reaction, the mixture was cooled to an ambient temperature. The solvent was removed under reduced pressure. The crude product obtained was taken up in DCM (50 mL), washed with water (100), and Brine (2 x 15 mL). The organic layer was then dried over anhydrous MgSO4, and the salt was filtered off. The crude product was purified by normal phase ISCO Teledyne CombiFlash system (80 g SiO_2 cartrige) column chromatography using increasingly polar mixtures petroleum ether and ethyl acetate as eluents. Fractions containing desired compound were combined and the solvent was evaporated under reduced pressure to the product **14** (3510 mg, 76 %) as a yellow oil which was used without further purification. LC-MS: t_R = 0. 853 min (Purity = 75%); *m/z* = 178.1 $[M+H]^+$ (anal. calcd. For $C_{10}H_{11}NO_2$: *m/z* = 177.1).

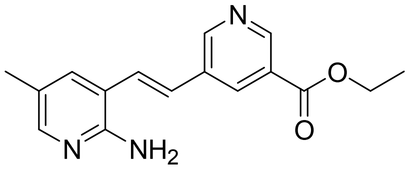

Ethyl (*E*)-5-(2-(2-amino-5-methylpyridin-3-yl)vinyl)nicotinate (15)

To a 10 mL microwave tube equipped with a stirrer bar, were added ethyl 5-ethenylpyridine-3-carboxylate (500 mg, 2.82 mmol), 2-amino-3-bromo-5-methylpyridine (686 mg, 3.67 mmol), tris(dibenzylideneacetone)dipalladium(0) (258 mg, 0.28 mmol), Et_3N (0.79 mL, 5.64 mmol). Toluene (5 mL) was added, and the mixture was degassed with N_2 for 10 minutes. The reactants were further commissioned in a microwave reactor 120 °C for 1 h, (LC-MS monitored). After the completion of the reaction, the mixture was cooled to an ambient temperature. The solvent was removed under reduced pressure. The crude product obtained was taken up in DCM (50 mL), washed with water (100), and Brine (2 x 15 mL). The organic layer was then dried over anhydrous MgSO4, and the salt was filtered off. The crude product was purified by normal phase ISCO Teledyne CombiFlash system (80 g SiO_2 cartrige) column chromatography using increasingly polar mixtures petroleum ether and ethyl acetate as eluents. Fractions containing desired compound were combined, and the solvent was evaporated under reduced pressure to give **15** (102 mg, 12.76 %) as orange powder which was used for the next reaction without further purification. LC-MS: t_R = 0. 656 min (Purity = 95%); *m/z* = 284.2 $[M+H]^+$ (anal. calcd. For $C_{16}H_{17}N_3O_2$: *m/z* = 283.2).

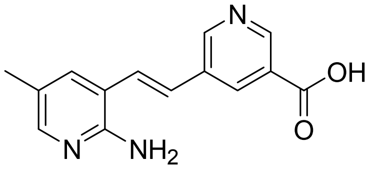

(*E*)-5-(2-(2-amino-5-methylpyridin-3-yl)vinyl)nicotinic acid (16)

To a 50 mL round bottom flask equipped with a stirrer bar were added a solution of ethyl 5-[(*E*)-2-(2-amino-5-methyl-3-pyridinyl)ethenyl]pyridine-3-carboxylate (600 mg, 2.12 mmol) in methanol (15 mL) followed by 1M solution of sodium hydroxide (423 mg, 10.59 mmol) in water (10 mL). The mixture was stirred at 70 °C for 1 h (LC-MS monitored). After the completion of the reaction, the mixture was cooled to ambient temperature. Solvent was reduced under on rotary evaporator to about 5 mL. The pH of the solutions was adjusted to 3 using 2M aqueous HCl. The aqueous solvent was removed under reduced pressure. The crude product was purified by reverse phase Biotage column chromatography using 5% MeCN in milli-q water as eluents. Test tubes containing desired compound were combined and the solvent reduced to about 20 mL, then freeze dried to afford **16** (532 mg, 96 %) as a yellow solid which was used without in the next step without further purification. LC-MS: t_R = 0. 309 min (Purity = 94%); *m/z* = 256.2 $[M+H]^+$ (anal. calcd. For $C_{14}H_{13}N_3O_2$: *m/z* = 256.1).

Synthesis of target compounds

***(E)-5-(2-(2-amino-5-methylpyridin-3-yl)vinyl)-N-(2,4-dichlorophenethyl)nicotinamide* (GSK-190937/ 4)**

Intermediate 3 (409 mg, 1.27 mmol), 2-amino-3-bromo-5-methylpyridine (833 mg, 4.46 mmol), $Pd(OAC)_2$ (114 mg, 0.50 mmol), PPh_3 (200.4 mg, 0.76 mmol), and Et_3N (3.0 mL, 21.5

mmol) were reacted according to the Method A to afford **4** as a yellow solid (58 mg, 10%); ^{1}H NMR (300 MHz, DMSO-*d*$_6$) δ_H 8.89 (s, 1H), 8.77 (s, 2H), 8.42 (s, 1H), 7.78 (s, 1H), 7.63 (d, *J* = 17.5 Hz, 2H), 7.59 – 7.46 (m, 1H), 7.39 (d, *J* = 2.3 Hz, 2H), 7.16 (d, *J* = 14.6 Hz, 1H), 6.02 (s, 2H), 3.65 – 3.49 (m, 2H), 2.99 (t, *J* = 6.7 Hz, 2H), 2.16 (s, 3H). LC-MS: t_R = 0.818 min (Purity = 100%); *m/z* = 427.1 [M+H]$^+$ (anal. calcd. for $C_{22}H_{20}Cl_2N_4O$: *m/z* = 426.1).

(E)-5-(2-Amino-5-methylstyryl)-N-(2,4-dichlorophenethyl)nicotinamide **(5)**
Intermediate **3** (30 mg, 0.09 mmol), 2-bromo-4-methylaniline (52 mg, 0.28 mmol) Pd(OAC)$_2$ (4 mg, 0.02 mmol), PPh_3 (9.8 mg, 0.04 mmol) and Et_3N (0.03 mL, 0.19 mmol) were reacted according to Method A to afford **5** as a yellow solid (7 mg, 17% yield); ^{1}H NMR (300 MHz, MeOD-*d*$_4$) δ_H 8.78 (d, *J* = 2.1 Hz, 1H), 8.69 (d, *J* = 2.0 Hz, 1H), 8.36 – 8.33 (m, 1H), 7.52 (d, *J* = 16.2 Hz, 1H), 7.42 (d, *J* = 2.1 Hz, 1H), 7.33 – 7.28 (m, 2H), 7.27 – 7.22 (m, 1H), 7.03 (d, *J* = 16.2 Hz, 1H), 6.89 (dd, *J* = 8.2, 2.0 Hz, 1H), 6.68 (d, *J* = 8.1 Hz, 1H), 3.65 (t, *J* = 7.1 Hz, 2H), 3.06 (t, *J* = 7.1 Hz, 2H), 2.22 (s, 3H). ^{13}C NMR (151 MHz, MeOD-*d*$_4$) δ_C 166.59, 149.45, 145.44, 143.01, 135.60, 134.60, 134.48, 132.73, 131.99, 131.69, 130.51, 129.90, 128.77, 128.19, 127.47, 126.99, 126.17, 122.61, 122.28, 116.84, 39.25, 32.23, 19.17. LC-MS: t_R = 1.142 min (Purity = 98%); *m/z* = 426.1 [M+H]$^+$ (anal. calcd. for $C_{23}H_{21}Cl_2N_3O$: *m/z* = 426.1).

(E)-N-(2,4-Dichlorophenethyl)-5-(2-(5-methylpyridin-3-yl)vinyl)nicotinamide **(6)**
Intermediate **3** (30 mg, 0.09 mmol), 3-bromo-5-methylpyridine (48 mg, 0.28 mmol), Pd(OAC)$_2$ (4 mg, 0.02 mmol), PPh_3 (9.8 mg, 0.04 mmol), and Et_3N (0.03 mL, 0.19 mmol) were reacted according to Method A to afford **6** as a white solid (17 mg, 42% yield); ^{1}H NMR (300 MHz, MeOD-*d*$_4$) δ_H 8.90 – 8.78 (m, 2H), 8.56 (s, 1H), 8.43 (s, 1H), 8.33 (s, 1H), 7.99 (s, 1H), 7.54 – 7.25 (m, 5H), 3.76 – 3.60 (m, 2H), 3.12 (t, 2H), 2.43 (s, 3H). ^{13}C NMR (151 MHz, MeOD-*d*$_4$) δ_C 166.34, 149.90, 148.53, 146.50, 144.92, 135.59, 134.62, 134.23, 134.18, 133.14, 132.72, 132.57, 132.03, 132.02, 130.57, 128.77, 128.12, 126.99, 125.97, 39.25, 32.20, 16.88. LC-MS: t_R = 1.142 min (Purity = 96%); *m/z* = 412.1 [M+H]$^+$ (anal. calcd. for $C_{22}H_{19}Cl_2N_3O$: *m/z* = 412.1).

(E)-5-(2-(2-Aminopyridin-3-yl)vinyl)-N-(2,4-dichlorophenethyl)nicotinamide **(7)**
Following Method A, intermediate **3** (30.0 mg, 0.09 mmol), 2-amino-3-bromopyridine (49 mg, 0.28 mmol), Pd(OAC)$_2$ (4 mg, 0.02 mmol), PPh_3 (10 mg, 0.04 mmol), and Et_3N (0.03 mL, 0.19 mmol) were reacted according to the to afford **6** as a yellow solid (16 mg, 40% yield); ^{1}H NMR (300 MHz, MeOD-*d*$_4$) δ_H 8.87 (d, *J* = 2.7 Hz, 1H), 8.77 (d, *J* = 2.3 Hz, 1H), 8.43 (s, 1H), 7.97 – 7.83 (m, 2H), 7.48 (t, *J* = 8.2 Hz, 2H), 7.41 – 7.25 (m, 2H), 7.17 (d, *J* = 15.9 Hz, 1H), 6.73 (t, *J* = 6.0 Hz, 1H), 3.70 (t, *J* = 6.8 Hz, 2H), 3.11 (t, *J* = 7.0 Hz, 2H). ^{13}C NMR (101 MHz, MeOD-*d*$_4$) δ_C 166.47, 156.89, 156.69, 149.65, 146.85, 135.60, 134.63, 134.60, 133.84, 133.07, 132.70, 132.05, 130.60, 130.30, 128.79, 127.03, 126.32, 117.17, 116.37, 39.29, 32.25. LC-MS: t_R = 0.785 min (Purity = 97%); *m/z* = 413.1 [M+H]$^+$ (anal. calcd. for $C_{21}H_{18}Cl_2N_4O$: *m/z* = 413.1).

(E)-5-(2-(6-Aminopyridin-3-yl)vinyl)-N-(2,4-dichlorophenethyl)nicotinamide (8)
Intermediate **3** (150 mg, 0.46 mmol), 3-bromopyridin-4-amine (323 mg, 1.87 mmol), $Pd(OAC)_2$ (32 mg, 0.14 mmol), PPh_3 (74 mg, 0.28 mmol), and Et_3N (0.5 mL, 3.59 mmol) were reacted following Method A to afford **8** as an off-white solid(17 mg, 9% yield); ^1H NMR (300 MHz, MeOD-*d4)* δ_H 8.75 (s, 1H), 8.72 (s, 1H), 8.32 (s, 1H), 8.05 (s, 1H), 7.87 (d, *J* = 8.8 Hz, 1H), 7.46 (s, 1H), 7.39 – 7.19 (m, 4H), 7.06 (d, *J* = 16.1 Hz, 1H), 6.66 (d, *J* = 7.4 Hz, 1H), 3.67 (t, *J* = 6.6 Hz, 2H), 3.09 (t, *J* = 6.2 Hz, 2H). LC-MS: t_R = 0.756 min (Purity = 100%); *m/z* = 413.0 $[M+H]^+$ (anal. calcd. for $C_{21}H_{18}Cl_2N_4O$: *m/z* = 412.0).

(E)-5-(2-(5-Aminopyridin-3-yl)vinyl)-N-(2,4-dichlorophenethyl)nicotinamide (9)
Intermediate **3** (20 mg, 0.06 mmol), 3-amino-5-bromopyridine (32 mg, 0.19 mmol), $Pd(OAC)_2$ (3 mg, 0.02 mmol), PPh_3 (6.53 mg, 0.02 mmol), and Et_3N (0.02 mL, 0.12 mmol) were reacted according to the Method A to afford **9** as a white solid (5 mg, 19% yield); ^1H NMR (600 MHz, MeOD-*d4)* δ_H 8.80 (d, *J* = 2.1 Hz, 1H), 8.76 (d, *J* = 2.0 Hz, 1H), 8.37 – 8.36 (m, 1H), 7.98 (d, *J* = 1.8 Hz, 1H), 7.89 (d, *J* = 2.5 Hz, 1H), 7.43 (d, *J* = 2.1 Hz, 1H), 7.33 – 7.25 (m, 5H), 3.66 (t, *J* = 7.1 Hz, 2H), 3.08 (t, *J* = 7.1 Hz, 2H). ^{13}C NMR (151 MHz, MeOD-*d4)* δ_C 166.37, 149.82, 146.38, 145.13, 136.36, 135.67, 135.61, 134.62, 133.28, 133.05, 132.73, 132.02, 131.91, 130.57, 128.78 (2 x C), 126.98, 125.25, 117.78, 39.24, 32.20. LC-MS: t_R = 0.753 min (Purity = 100%); *m/z* = 413.1 $[M+H]^+$ (anal. calcd. for $C_{21}H_{18}Cl_2N_4O$: *m/z* = 413.1).

(E)-N-(2,4-dichlorophenethyl)-5-(2-(pyridin-3-yl)vinyl)nicotinamide (10)
Intermediate **3** (30 mg, 0.09 mmol), 3-bromopyridine (0.03 mL , 0.28 mmol), $Pd(OAC)_2$ (4 mg, 0.02 mmol), PPh_3 (10 mg, 0.04 mmol), and Et_3N (0.03 mL, 0.19 mmol) were reacted according to the Method A to afford **10** as a brown solid (29 mg, 76% yield); ^1H NMR (300 MHz, MeOD-*d4)* δ_H 8.95 – 8.78 (m, 2H), 8.77 (s, 1H), 8.53 – 8.41 (m, 2H), 8.16 (d, *J* = 8.1 Hz, 1H), 7.61 – 7.41 (m, 4H), 7.41 – 7.25 (m, 2H), 3.70 (t, *J* = 7.0 Hz, 2H), 3.12 (t, *J* = 7.4 Hz, 2H). ^{13}C NMR (101 MHz, MeOD-*d4)* δ_C 166.33, 149.93, 148.11, 147.70, 146.57, 135.59, 134.62, 133.86, 133.07, 132.72, 132.10, 132.02, 130.58, 128.78, 128.05, 127.92, 127.00, 126.22, 124.08, 39.26, 32.20. LC-MS: t_R = 0.958 min (Purity = 98%); *m/z* = 398.1 $[M+H]^+$ (anal. calcd. for $C_{21}H_{17}Cl_2N_3O$: *m/z* = 398.1).

(E)-N-(2,4-dichlorophenethyl)-5-styrylnicotinamide (11)
Intermediate **3** (30 mg, 0.09 mmol), bromobenzene (0.03 mL , 0.28 mmol), $Pd(OAC)_2$ (4 mg, 0.02 mmol), PPh_3 (10 mg, 0.04 mmol), and Et_3N (0.03 mL, 0.19 mmol) were reacted according to Method A to afford **11** as a yellow solid (26 mg, 66% yield); ^1H NMR (300 MHz, MeOD-*d4)* δ_H 8.91 – 8.75 (m, 2H), 8.40 (d, *J* = 2.1 Hz, 1H), 7.68 – 7.59 (m, 2H), 7.51 – 7.19 (m, 8H), 3.70 (t, *J* = 7.1 Hz, 2H), 3.11 (t, *J* = 7.1 Hz, 2H). ^{13}C NMR (101 MHz, MeOD-*d4)* δ_C 166.48, 149.67, 146.79, 145.96, 136.48, 135.62, 134.62, 133.79, 132.73, 132.36, 132.03, 131.67, 130.54, 128.78, 128.43, 128.16, 127.62, 126.99, 126.56, 123.16, 39.23, 32.20. LC-MS: t_R = 1.300 min (Purity = 95%); *m/z* = 397.1 $[M+H]^+$ (anal. calcd. for $C_{22}H_{18}Cl_2N_2O$: *m/z* = 397.1).

(E)-5-(2-(4-aminopyridin-3-yl)vinyl)-N-(2,4-dichlorophenethyl)nicotinamide (12)

Intermediate **3** (200 mg, 0.62 mmol), 3-bromopyridin-4-amine (323 mg, 1.87 mmol), $Pd(OAC)_2$ (42 mg, 0.62 mmol), PPh_3 (97 mg, 0.37 mmol), and Et_3N (0.5 mL, 3.59 mmol) were reacted according to the Method A to afford **12** as an off-white solid (100 mg, 38% yield); ^1H NMR (300 MHz, MeOD-*d*$_4$) δ_H 8.88 (s, 1H), 8.81 (s, 1H), 8.51 (s, 1H), 8.47 – 8.36 (m, 2H), 8.00 (d, *J* = 6.7 Hz, 1H), 7.43 (d, *J* = 15.1 Hz, 2H), 7.34 (d, *J* = 7.1 Hz, 1H), 7.29 – 7.18 (m, 2H), 6.88 (d, *J* = 6.4 Hz, 1H), 3.68 (t, *J* = 6.8 Hz, 2H), 3.09 (t, *J* = 7.5 Hz, 2H). LC-MS: t_R = 0.747 min (Purity = 100%); *m/z* = 413.1 $[M+H]^+$ (anal. calcd. for $C_{21}H_{18}Cl_2N_4O$: *m/z* = 413.1).

(E)-5-(2-(4-aminopyrimidin-5-yl)vinyl)-N-(2,4-dichlorophenethyl)nicotinamide (13)
Intermediate **3** (200 mg, 0.62 mmol), 3-bromopyridin-4-amine (325 mg, 1.87 mmol), $Pd(OAC)_2$ (42 mg, 0.19 mmol), PPh_3 (97 mg, 0.37 mmol), and Et_3N (0.5 mL, 3.59 mmol) were reacted according to the Method A to afford **13** as an off-white solid (17 mg, 7% yield); ^1H NMR (300 MHz, DMSO) δ_H8.92 – 8.73 (m, 3H), 8.55 – 8.22 (m, 3H), 7.59 (s, 1H), 7.49 – 7.09 (m, 5H), 3.64 – 3.41 (m, 2H), 3.07 – 2.85 (m, 2H). (s, 3H). LC-MS: t_R = 0.752 min (Purity = 100%); *m/z* = 414.1 $[M+H]^+$ (anal. calcd. for $C_{20}H_{17}Cl_2N_5O$: *m/z* = 414.1).

(E)-5-(2-(2-amino-5-methylpyridin-3-yl)vinyl)-N-(2,4-dichlorophenethyl)nicotinamide (17)
Intermediate **16** (25 mg, 0.1 mmol), 2-(2-chlorophenyl)ethanamine (22 mg, 0.15 mmol) , HATU (93 mg, 0.24 mmol), and Et_3N (0.03mL, 0.24 mmol) were reacted according to the Method B to afford **17** as a yellow solid (11 mg, 29% yield); ^1H NMR (300 MHz, MeOD-*d*$_4$) δ_H 8.86 (s, 1H), 8.77 (s, 1H), 8.43 (d, *J* = 2.6 Hz, 1H), 7.80 – 7.72 (m, 2H), 7.55 – 7.12 (m, 6H), 3.69 (t, *J* = 7.3 Hz, 2H), 3.13 (t, *J* = 5.6 Hz, 2H), 2.25 (s, 3H). ^{13}C NMR (101 MHz, MeOD-*d*$_4$) δ_C 166.47, 154.91, 150.89, 149.59, 146.48, 145.94, 138.50, 136.61, 135.35, 133.80, 132.10, 130.95, 129.18, 127.88, 126.80, 126.33, 125.12, 122.86, 116.94, 39.51, 32.72, 15.90.
LC-MS: t_R = 0.751 min (Purity = 97%); *m/z* = 393.1 $[M+H]^+$ (anal. calcd. for $C_{23}H_{22}ClN_3O$: *m/z* = 393.1).

(E)-5-(2-(2-amino-5-methylpyridin-3-yl)vinyl)-N-(4-chlorophenethyl)nicotinamide (18)
Intermediate **16** (25 mg, 0.1 mmol), 2-(4-chlorophenyl)ethanamine (22 mg, 0.15 mmol) , HATU (93 mg, 0.24 mmol), and Et_3N (0.03mL, 0.24 mmol) were reacted according to the Method B to afford **18** as a yellow solid (23 mg, 59% yield); ^1H NMR (300 MHz, MeOD-*d*$_4$) δ_H 8.97 (s, 1H), 8.88 (s, 1H), 8.66 (s, 1H), 8.28 (s, 1H), 7.73 (s, 1H), 7.67 – 7.38 (m, 2H), 7.38 – 7.24 (m, 4H), 3.67 (t, *J* = 7.0 Hz, 2H), 2.97 (t, *J* = 7.2 Hz, 2H), 2.35 (s, 3H). ^{13}C NMR (101 MHz, MeOD-*d*$_4$) δ_C 165.94, 150.93, 149.78, 146.54, 141.83, 137.86 (2C), 133.32, 132.88, 132.73, 131.87, 130.88, 130.16, 129.54, 128.18 (2C), 123.38, 122.27, 122.05, 41.07, 34.33, 15.61. LC-MS: t_R = 0.752 min (Purity = 100%); *m/z* = 393.2 $[M+H]^+$ (anal. calcd. for $C_{22}H_{21}ClN_4O$: *m/z* = 393.1).

(E)-5-(2-(2-amino-5-methylpyridin-3-yl)vinyl)-N-phenethylnicotinamide (19)
Intermediate **16** (90 mg, 0.35 mmol), phenethylamine (85 mg, 0.71 mmol) , HATU (335 mg, 0.88 mmol), and Et_3N (0.12 mL, 0.88 mmol) were reacted according to the Method B to afford **19** as a yellow powder (20 mg, 16 %); ^1H NMR (300 MHz, MeOD-*d*$_4$) δ_H 8.87 (s, 1H), 8.77 (s,

1H), 8.44 (s, 1H), 7.78 (s, 2H), 7.47 (d, *J* = 17.0 Hz, 1H), 7.37 – 7.14 (m, 6H), 3.66 (t, *J* = 7.7 Hz, 2H), 2.97 (t, *J* = 7.5 Hz, 2H), 2.26 (s, 3H). LC-MS: t_R = 0.702 min (Purity = 100%); *m/z* = 359.2 $[M+H]^+$ (anal. calcd. for $C_{22}H_{22}N_4O$: *m/z* = 358.2).

(E)-5-(2-(2-amino-5-methylpyridin-3-yl)vinyl)-N-(2,4-dichlorobenzyl)nicotinamide **(20)**
Intermediate **16** (100 mg, 0.39 mmol), 2,4-dichlorobenzylamine (0.13 mL, 0.98 mmol), HATU (372 mg, 0.98 mmol), and Et_3N (0.14 mL, 0.98 mmol) were reacted according to the Method B to afford **20** as a yellow powder (20 mg, 12%) ; ^1H NMR (300 MHz, MeOD-*d₄*) δ_H 8.91 (s, 2H), 8.54 (s, 1H), 7.79 (d, *J* = 13.6 Hz, 2H), 7.56 – 7.45 (m, 3H), 7.37 (d, *J* = 8.1 Hz, 1H), 7.24 (d, *J* = 15.1 Hz, 1H), 4.71 (s, 2H), 2.27 (s, 3H). LC-MS: t_R = 0.775 min (Purity = 100%); *m/z* = 412.1 $[M+H]^+$ (anal. calcd. for $C_{21}H_{18}Cl_2N_4O$: *m/z* = 413.1).

(E)-5-(2-(2-amino-5-methylpyridin-3-yl)vinyl)-N-(3,5-dichlorobenzyl)nicotinamide **(21)**
Intermediate **16** (50 mg, 0.20 mmol), 3,5-dichlorophenyl)methanamine (51 mg, 0.29 mmol), HATU (186 mg, 0.49 mmol) , and Et_3N (0.07mL, 0.49 mmol) were reacted according to the Method B to afford **21** as a yellow powder (4 mg, 5%); ^1H NMR (300 MHz, MeOD-*d₄*) δ_H 8.89 (s, 2H), 8.53 (s, 1H), 7.77 (s, 2H), 7.50 (d, *J* = 17.7 Hz, 1H), 7.38 (s, 3H), 7.21 (d, *J* = 16.1 Hz, 1H), 4.61 (s, 2H), 2.25 (s, 3H). LC-MS: t_R = 0.918 min (Purity = 100%); *m/z* = 412.1 $[M+H]^+$ (anal. calcd. for $C_{21}H_{18}Cl_2N_4O$: *m/z* = 413.1).

(E)-5-(2-(2-amino-5-methylpyridin-3-yl)vinyl)-N-(2,4-dichlorophenyl)nicotinamide **(22)**
N-(2,4-dichlorophenyl)-5-ethenylpyridine-3-carboxamide (211 mg, 0.72 mmol), 2-amino-3-bromo-5-methylpyridine, (404 mg, 2.16 mmol), $Pd(OAC)_2$ (65 mg, 0.29 mmol), PPh_3 (113 mg, 0.43 mmol), and Et_3N (1.7 mL, 12.2 mmol) were reacted according to the Method A to afford **22** as a yellow powder (28 mg, 10%); ^1H NMR (300 MHz, MeOD-*d₄*) δ_H 8.97 (d, *J* = 7.7 Hz, 2H), 8.61 (s, 1H), 7.78 (s, 3H), 7.64 (d, *J* = 2.9 Hz, 1H), 7.60 – 7.39 (m, 2H), 7.24 (d, *J* = 16.2 Hz, 1H), 2.26 (s, 3H). LC-MS: t_R = 0.760 min (Purity = 100%); *m/z* = 399.0 $[M+H]^+$ (anal. calcd. for $C_{20}H_{16}Cl_2N_4O$: *m/z* = 398.0).

(E)-5-(2-(2-amino-5-methylpyridin-3-yl)vinyl)-N-(4-chlorophenyl)nicotinamide **(23)**
N-(4-chlorophenyl)-5-ethenylpyridine-3-carboxamide (354 mg, 1.37 mmol), 2-amino-3-bromo-5-methylpyridine, (768 mg, 4.11 mmol), $Pd(OAC)_2$ (122 mg, 0.54 mmol), PPh_3 (215 mg, 0.82 mmol), and Et_3N (3.23 mL, 23.2 mmol) were reacted according to the Method A to afford **23** as a yellow powder (40 mg, 8%); ^1H NMR (300 MHz, MeOD-*d₄*) δ_H 8.97 (d, *J* = 7.7 Hz, 2H), 8.61 (s, 1H), 7.78 (s, 3H), 7.64 (d, *J* = 2.9 Hz, 1H), 7.60 – 7.39 (m, 2H), 7.24 (d, *J* = 16.2 Hz, 1H), 2.26 (s, 3H). LC-MS: t_R = 0.733 min (Purity = 100%); *m/z* = 365.1 $[M+H]^+$ (anal. calcd. for $C_{20}H_{17}ClN_4O$: *m/z* = 364.1).

(E)-5-(2-(2-amino-5-methylpyridin-3-yl)vinyl)-N-(2-(pyridin-4-yl)ethyl)nicotinamide **(24)**
Intermediate **16** (25 mg, 0.09 mmol), 4-(2-Aminoethyl)pyridine (17 mg, 0.15 mmol), HATU (93 mg, 0.25 mmol) , and Et_3N (0.034 mL, 0.25 mmol) were reacted according to the Method B to afford **24** as a yellow solid (21 mg, 58%); ^1H NMR (300 MHz, MeOD-*d₄*) δ_H 8.87 (s, 1H), 8.77 (s, 1H), 8.55 – 8.39 (m, 3H), 7.80 – 7.69 (m, 2H), 7.53 – 7.32 (m, 3H), 7.18 (d, *J* = 16.2

Hz, 1H), 3.73 (t, J = 8.5 Hz, 2H), 3.04 (t, J = 8.5 Hz, 2H), 2.25 (s, 3H). ^{13}C NMR (101 MHz, MeOD-d_4) δ_C 166.44, 154.75, 149.89, 149.73, 148.65, 148.58, 145.91, 145.81, 135.65, 133.83, 132.17, 130.51, 126.12, 125.24, 124.74, 124.68, 122.83, 117.14, 39.98, 34.34, 15.97. LC-MS: t_R = 0.223 min (Purity = 98%); m/z = 360.2 [M+H]$^+$ (anal. calcd. for $C_{21}H_{21}N_5O$: m/z = 360.2).

***(E)-5-(2-(2-amino-5-methylpyridin-3-yl)vinyl)-N-(2-(piperidin-4-yl)ethyl)nicotinamide* (25)**
To a stirred solution of *tert*-butyl 4-[2-[[5-[(*E*)-2-(2-amino-5-methyl-3-pyridinyl)ethenyl]pyridine-3-carbonyl]amino]ethyl]piperidine-1-carboxylate (100 mg, 0.21 mmol) in DCM (10 mL) was added trifluoroacetic acid (4.11mL, 53.69 mmol). The mixture was stirred at 25 °C for 1 h (LCMS monitored). After the completion of the reaction, the solvent was removed under reduced pressure. The crude product obtained was purified by reverse phase Biotage column chromatography using decreasingly polar mixtures of milli-q water and MeCN as eluents. Test tubes containing desired compound were combined, and the solvent reduced to about 20 mL, then freeze dried to give **25** (52 mg, 65%) as a yellow solid; ^{1}H NMR (300 MHz, MeOD-d_4) δ_H 8.99 – 8.88 (m, 2H), 8.56 (s, 1H), 8.26 (s, 1H), 7.72 (s, 1H), 7.58 – 7.38 (m, 2H), 3.59 – 3.37 (m, 4H), 3.01 (t, J = 12.9 Hz, 2H), 2.87 – 2.80 (m, 1H), 2.34 (s, 3H), 2.12 – 2.01 (m, 2H), 1.75 – 1.62 (m, 2H), 1.56 – 1.37 (m, 2H). ^{13}C NMR (101 MHz, MeOD-d_4) δ_C 166.20, 161.55, 151.12, 150.08, 146.89, 141.64, 132.74, 130.77, 129.60, 123.27, 122.00, 118.24, 115.33, 43.81, 36.79, 35.19, 31.16, 28.47, 15.59. LC-MS: t_R = 0.220 min (Purity = 98%); m/z = 366.2 [M+H]$^+$ (anal. calcd. for $C_{21}H_{27}N_5O$: m/z = 366.2).

***(E)-6-(2-(2-amino-5-methylpyridin-3-yl)vinyl)-N-(2,4-dichlorophenethyl)picolinamide* (26)**
N-[2-(2,4-dichlorophenyl)ethyl]-6-ethenylpyridine-2-carboxamide (30 mg, 0.09 mmol), 2-amino-3-bromo-5-methylpyridine (52 mg, 0.28 mmol), Pd(OAC)$_2$ (4.19 mg, 0.02 mmol), PPh_3 (9.8 mg, 0.037 mmol), and Et_3N (0.04 mL, 0.28 mmol) were reacted according to the Method A to afford **26** as a yellow powder (17 mg, 41%); ^{1}H NMR (300 MHz, MeOD-d_4) δ_H 7.92 – 7.85 (m, 4H), 7.75 (s, 1H), 7.67 – 7.64 (m, 1H), 7.43 (s, 1H), 7.35 – 7.21 (m, 3H), 3.72 (t, J = 7.0 Hz, 2H), 3.10 (t, J = 7.0 Hz, 2H), 2.26 (s, 3H). ^{13}C NMR (151 MHz, MeOD-d_4) δ_C 165.49, 154.45, 149.31, 144.56, 137.96, 136.43, 135.58, 134.61, 132.68, 131.97, 128.78, 128.74, 126.99, 126.94, 124.84, 122.91, 120.01, 119.96, 117.51, 38.70, 32.41, 15.87. LC-MS: t_R = 0.854 min (Purity = 100%); m/z = 427.1 [M+H]$^+$ (anal. calcd. for $C_{22}H_{20}Cl_2N_4O$: m/z = 427.1).

***(E)-3-(2-(2-amino-5-methylpyridin-3-yl)vinyl)-N-(2,4-dichlorophenethyl)benzamide* (27)**
N-(2,4-dichlorophenethyl)-3-vinylbenzamide (30 mg, 0.09 mmol), 2-amino-3-bromo-5-methylpyridine (52 mg, 0.28 mmol), Pd(OAC)$_2$ (4.19 mg, 0.02 mmol), PPh_3 (9.8 mg, 0.037 mmol), and Et_3N (0.04 mL, 0.28 mmol) were reacted according to the Method A to afford **27** as a yellow powder (20 mg, 50%); ^{1}H NMR (300 MHz, MeOD-d_4) δ_H 8.86 (s, 1H), 7.96 (d, J = 8.5 Hz, 1H), 7.76 (s, 2H), 7.46 (s, 1H), 7.32 (dd, J = 9.5, 7.3 Hz, 3H), 7.13 (s, 1H), 6.83 (s, 2H), 3.65 (t, J = 7.3 Hz, 2H), 3.06 (d, J = 7.2 Hz, 2H), 2.07 (s, 3H). LC-MS: t_R = 0.867 min (Purity = 100%); m/z = 426.1 [M+H]$^+$ (anal. calcd. for $C_{23}H_{21}Cl_2N_3O$: m/z = 425.1).

***(E)-4-(2-(2-amino-5-methylpyridin-3-yl)vinyl)-N-(2,4-dichlorophenethyl)thiophene-3-carboxamide* (28)**

N-[2-(2,4-dichlorophenyl)ethyl]-4-ethenylthiophene-3-carboxamide (30 mg, 0.09 mmol), 2-amino-3-bromo-5-methylpyridine (52 mg, 0.28 mmol), $Pd(OAC)_2$ (4 mg, 0.02 mmol), PPh_3 (9.65 mg, 0.04 mmol), and Et_3N (0.03 mL, 0.18 mmol) were reacted according to the Method A to afford **28** as a yellow solid (12 mg, 29%); ^{1}H NMR (300 MHz, MeOD-*d$_4$*) δ_H 7.79 – 7.70 (m, 4H), 7.46 – 7.23 (m, 4H), 7.06 (d, *J* = 16.3 Hz, 1H), 3.64 (t, *J* = 6.6 Hz, 2H), 3.07 (t, *J* = 6.5 Hz, 2H), 2.25 (s, 3H). ^{13}C NMR (151 MHz, MeOD-*d$_4$*) δ_C 166.00, 154.09, 143.58, 139.44, 135.91, 135.66, 134.61, 132.62, 132.02, 128.73, 127.82, 127.40, 126.89, 126.64, 124.60, 124.23, 123.26, 121.74, 38.80, 32.33, 15.93. LC-MS: t_R = 0.830 min (Purity = 100%); *m/z* = 432.1 $[M+H]^+$ (anal. calcd. for $C_{21}H_{19}Cl_2N_3OS$: *m/z* = 432.1).

***(E)-N-(5-(2-(2-amino-5-methylpyridin-3-yl)vinyl)pyridin-3-yl)-3-(2,4-dichlorophenyl)propenamide* (29)**

3-(2,4-dichlorophenyl)-*N*-(5-ethenyl-3-pyridinyl)propanamide (50 mg, 0.16 mmol), 2-amino-3-bromo-5-methylpyridine (87 mg, 0.47 mmol), $Pd(OAC)_2$ (7 mg, 0.03 mmol), PPh_3 (16 mg, 0.06 mmol), and Et_3N (0.04 mL, 0.31 mmol) were reacted according to the Method A to afford **29** as a yellow solid (31 mg, 45%); ^{1}H NMR(300 MHZ, MeOD-*d$_4$*) δ_H 8.49 – 8.46 (m, 2H), 8.37 (s, 1H), 7.74 (d, *J* = 5.8 Hz, 2H), 7.46 (s, 1H), 7.41 – 7.23 (m, 3H), 7.10 (d, *J* = 16.1 Hz, 1H), 3.15 (t, *J* = 7.6 Hz, 2H), 2.77 (t, *J* = 7.7 Hz, 2H), 2.24 (s, 3H). ^{13}C NMR (151 MHz, MeOD-*d$_4$*) δ_C 171.94, 154.75, 145.96, 142.62, 139.26, 137.05, 135.82, 135.47, 134.30, 133.97, 132.58, 131.43, 128.75, 127.00, 125.92, 125.54, 124.07, 122.91, 117.27, 35.82, 28.19, 15.89.LC-MS: t_R = 0.789 min (Purity = 100%); *m/z* = 427.1 $[M+H]^+$ (anal. calcd. for $C_{22}H_{20}Cl_2N_4O$: *m/z* = 427.1).

2. Analytical data of final compounds

HPLC_UV and MS spectra of **GSK-190937/4**

Additional Info : Peak(s) manually integrated

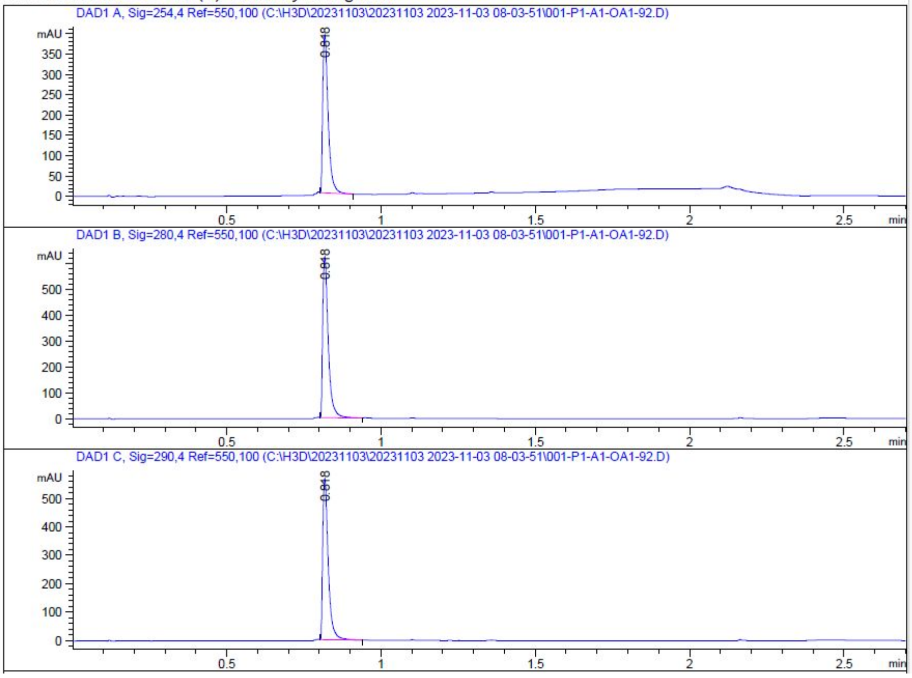

Signal 1: DAD1 A, Sig=254,4 Ref=550,100

Peak #	RetTime [min]	Type	Width [min]	Area [mAU*s]	Height [mAU]	Area %
1	0.818	BB	0.0187	471.43375	387.55301	100.0000
Totals :				471.43375	387.55301	

Signal 2: DAD1 B, Sig=280,4 Ref=550,100

Peak #	RetTime [min]	Type	Width [min]	Area [mAU*s]	Height [mAU]	Area %
1	0.818	BB	0.0190	771.51654	620.96729	100.0000
Totals :				771.51654	620.96729	

Signal 3: DAD1 C, Sig=290,4 Ref=550,100

Peak #	RetTime [min]	Type	Width [min]	Area [mAU*s]	Height [mAU]	Area %
1	0.818	BB	0.0190	698.98120	564.10107	100.0000
Totals :				698.98120	564.10107	

MS Spectrum

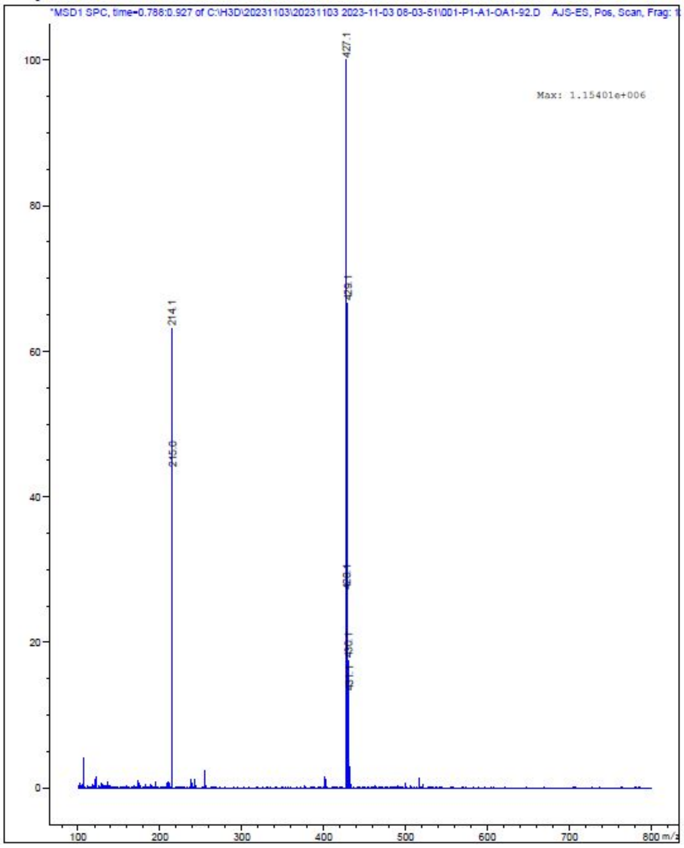

HPLC_UV and MS spectra of **5**

Additional Info : Peak(s) manually integrated

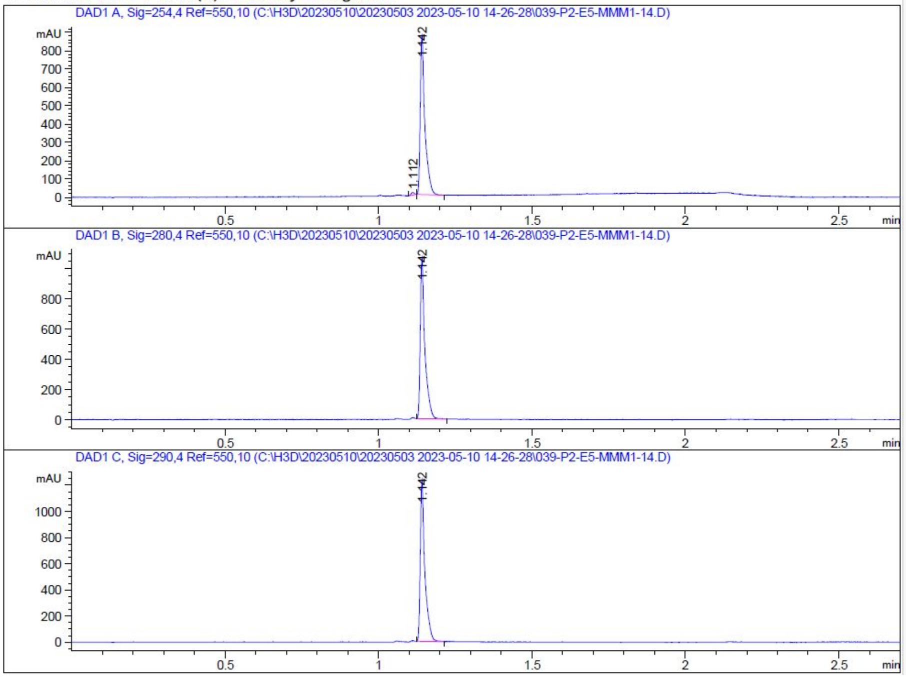

Signal 1: DAD1 A, Sig=254,4 Ref=550,10

Peak #	RetTime [min]	Type	Width [min]	Area [mAU*s]	Height [mAU]	Area %
1	1.112	BB	0.0119	9.88774	13.74242	1.0694
2	1.142	BB	0.0153	914.70935	865.37500	98.9306

Totals : 924.59709 879.11742

Signal 2: DAD1 B, Sig=280,4 Ref=550,10

Peak #	RetTime [min]	Type	Width [min]	Area [mAU*s]	Height [mAU]	Area %
1	1.142	BB	0.0153	1116.97058	1059.05054	100.0000

Totals : 1116.97058 1059.05054

Signal 3: DAD1 C, Sig=290,4 Ref=550,10

Peak #	RetTime [min]	Type	Width [min]	Area [mAU*s]	Height [mAU]	Area %
1	1.142	BB	0.0152	1284.27722	1225.37842	100.0000

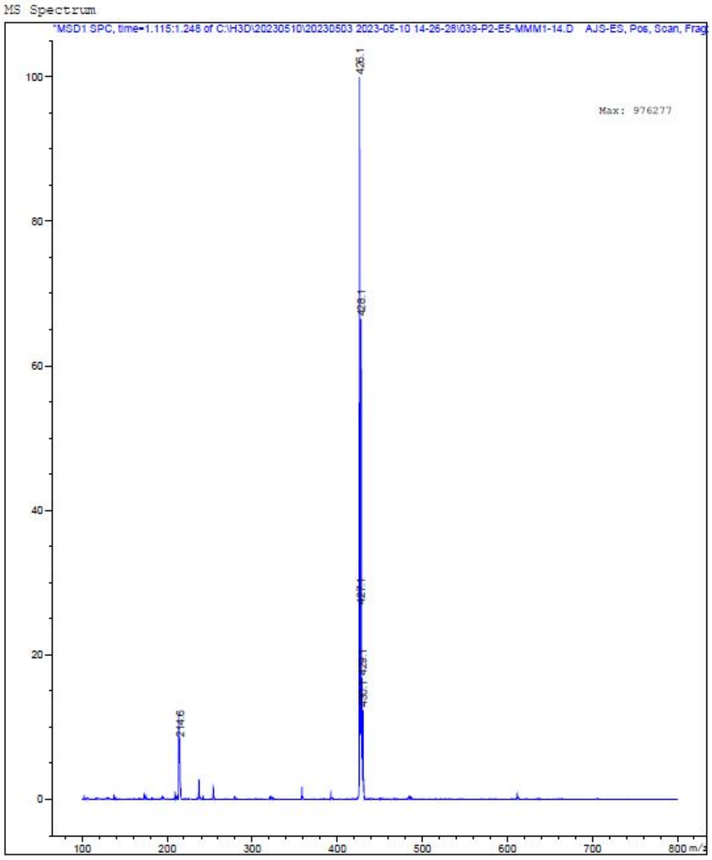

HPLC_UV and MS spectra of **6**

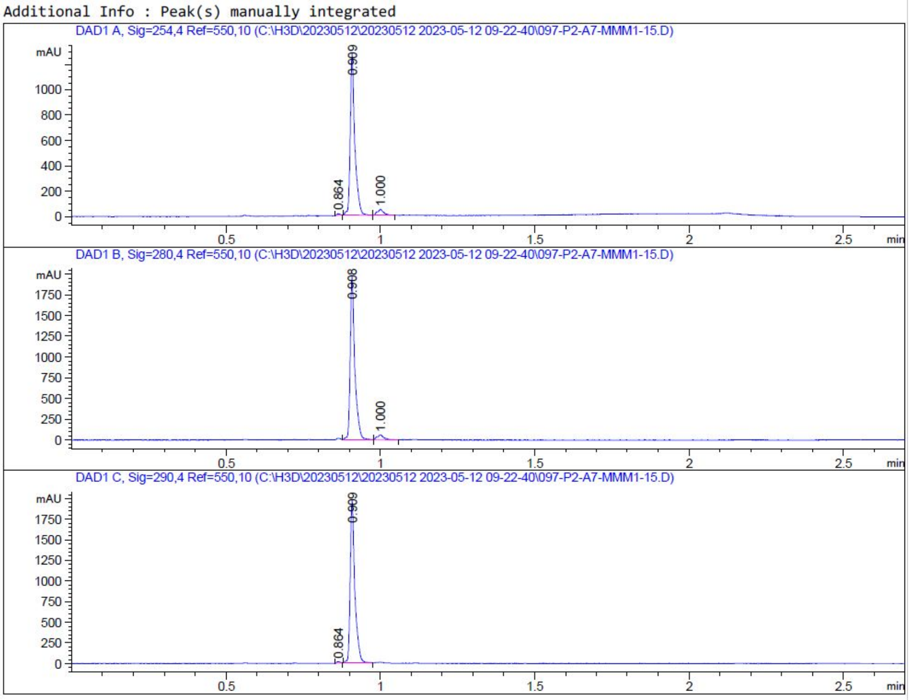

Signal 1: DAD1 A, Sig=254,4 Ref=550,10

Peak #	RetTime [min]	Type	Width [min]	Area [mAU*s]	Height [mAU]	Area %
1	0.864	BB	0.0131	9.07567	11.61669	0.6565
2	0.909	BB	0.0150	1306.09326	1266.37622	94.4708
3	1.000	BB	0.0189	67.36746	46.43729	4.8727

Totals : 1382.53639 1324.43020

Signal 2: DAD1 B, Sig=280,4 Ref=550,10

Peak #	RetTime [min]	Type	Width [min]	Area [mAU*s]	Height [mAU]	Area %
1	0.908	BB	0.0151	2020.30676	1955.88733	96.0704
2	1.000	BB	0.0197	82.63795	57.76205	3.9296

Totals : 2102.94471 2013.64938

Signal 3: DAD1 C, Sig=290,4 Ref=550,10

Peak #	RetTime [min]	Type	Width [min]	Area [mAU*s]	Height [mAU]	Area %
1	0.864	BB	0.0111	13.14273	17.85664	0.6353
2	0.909	BB	0.0152	2055.74316	1965.20386	99.3647

MS Spectrum

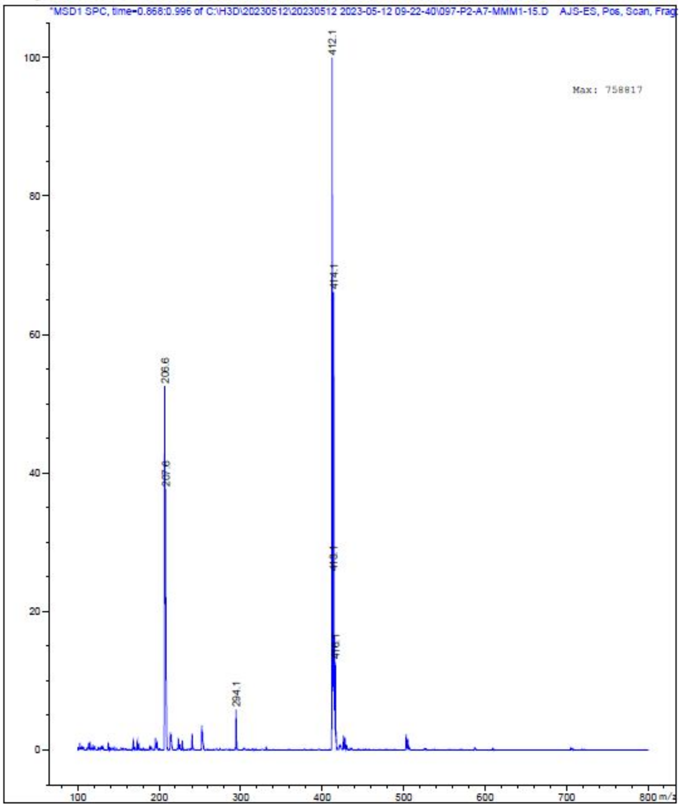

HPLC_UV and MS spectra of **7**

Additional Info : Peak(s) manually integrated

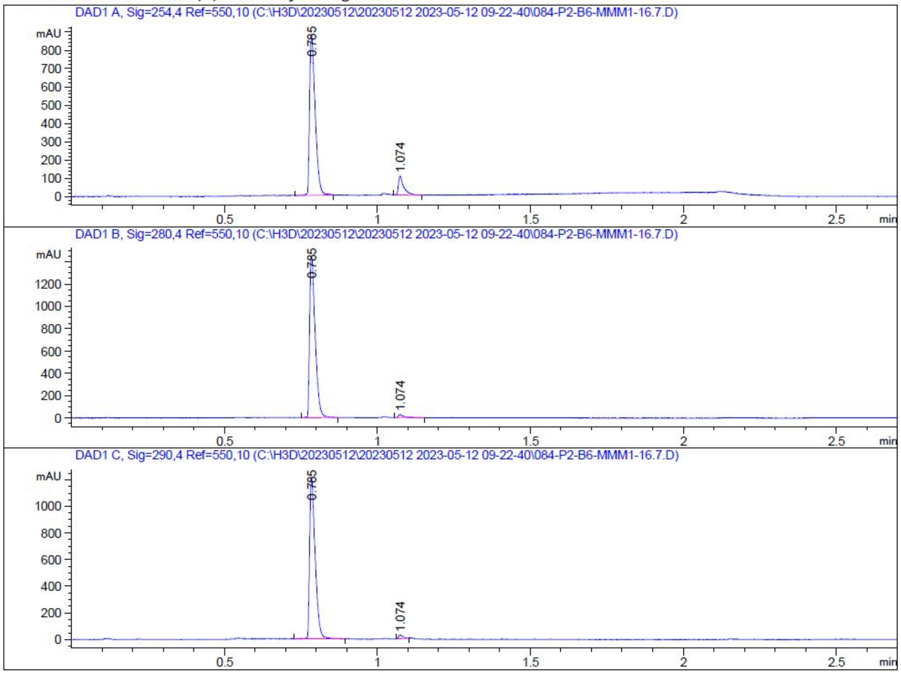

Signal 1: DAD1 A, Sig=254,4 Ref=550,10

Peak #	RetTime [min]	Type	Width [min]	Area [mAU*s]	Height [mAU]	Area %
1	0.785	BB	0.0194	1113.54822	873.65594	90.0554
2	1.074	BB	0.0175	122.96664	102.57011	9.9446
Totals :				1236.51485	976.22605	

Signal 2: DAD1 B, Sig=280,4 Ref=550,10

Peak #	RetTime [min]	Type	Width [min]	Area [mAU*s]	Height [mAU]	Area %
1	0.785	BB	0.0187	1823.84485	1450.65503	97.8671
2	1.074	BB	0.0207	39.74839	27.02620	2.1329
Totals :				1863.59324	1477.68123	

Signal 3: DAD1 C, Sig=290,4 Ref=550,10

Peak #	RetTime [min]	Type	Width [min]	Area [mAU*s]	Height [mAU]	Area %
1	0.785	BB	0.0188	1536.15161	1209.88684	98.3143
2	1.074	BB	0.0145	26.33911	26.82936	1.6857

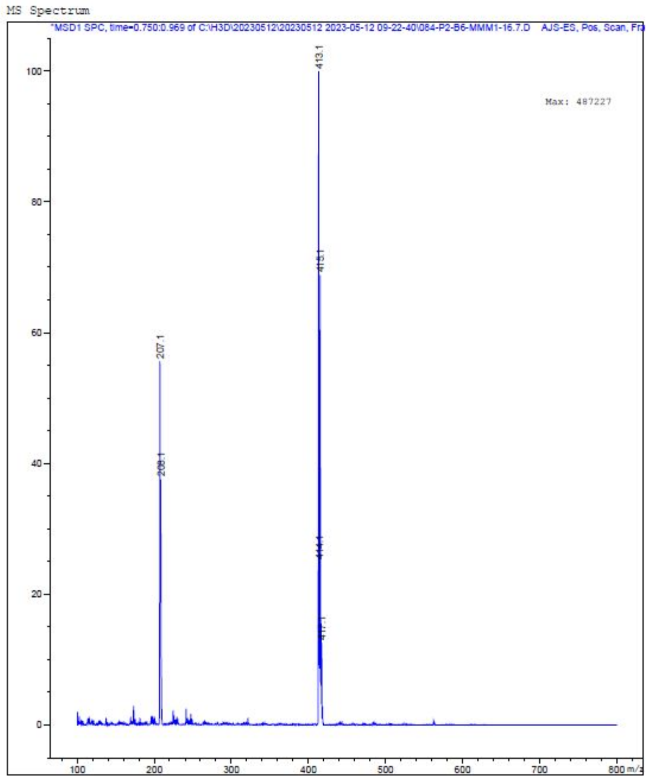

HPLC_UV and MS spectra of **8**

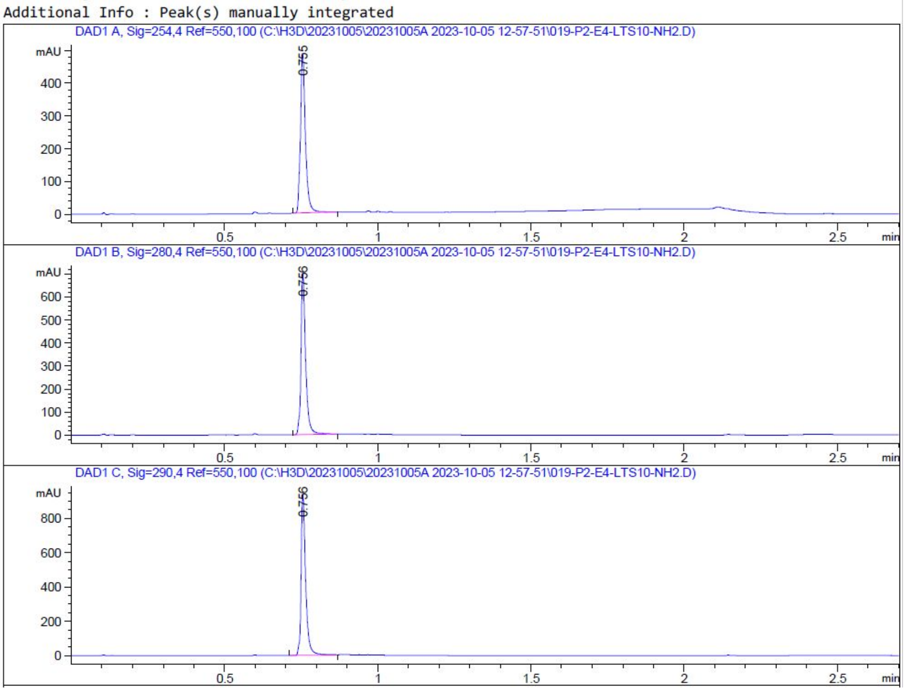

```
Signal 1: DAD1 A, Sig=254,4 Ref=550,100

Peak RetTime Type  Width     Area      Height     Area
  #   [min]        [min]   [mAU*s]     [mAU]       %
----|-------|----|-------|----------|----------|--------|
   1   0.755 BB    0.0168  556.16235  487.22961 100.0000

Totals :                   556.16235  487.22961


Signal 2: DAD1 B, Sig=280,4 Ref=550,100

Peak RetTime Type  Width     Area      Height     Area
  #   [min]        [min]   [mAU*s]     [mAU]       %
----|-------|----|-------|----------|----------|--------|
   1   0.756 BB    0.0159  738.46399  696.33490 100.0000

Totals :                   738.46399  696.33490


Signal 3: DAD1 C, Sig=290,4 Ref=550,100

Peak RetTime Type  Width     Area      Height     Area
  #   [min]        [min]   [mAU*s]     [mAU]       %
----|-------|----|-------|----------|----------|--------|
   1   0.756 BB    0.0155  963.18701  932.62299 100.0000

Totals :                   963.18701  932.62299
```

MS Spectrum

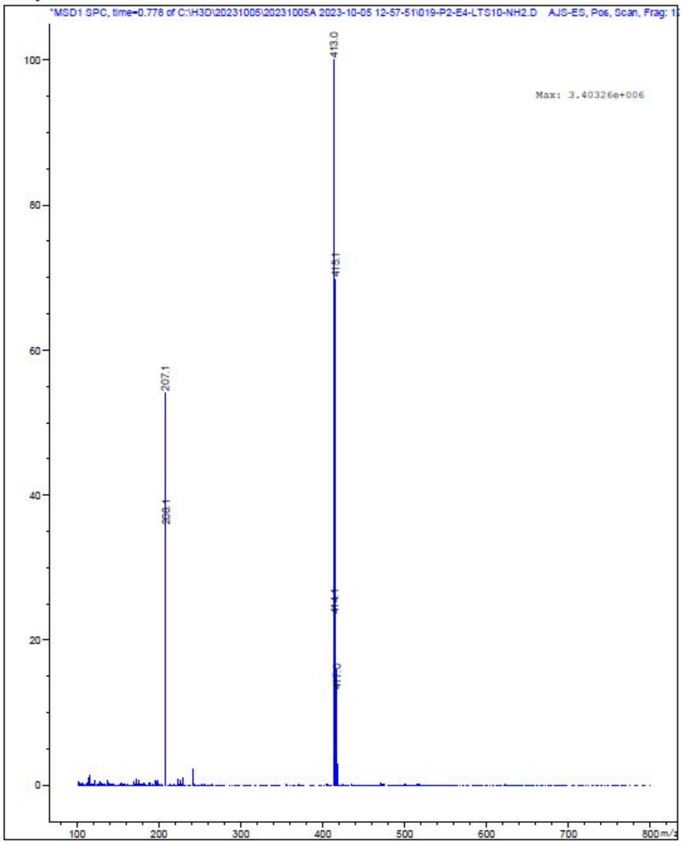

HPLC_UV and MS spectra of **9**

Additional Info : Peak(s) manually integrated

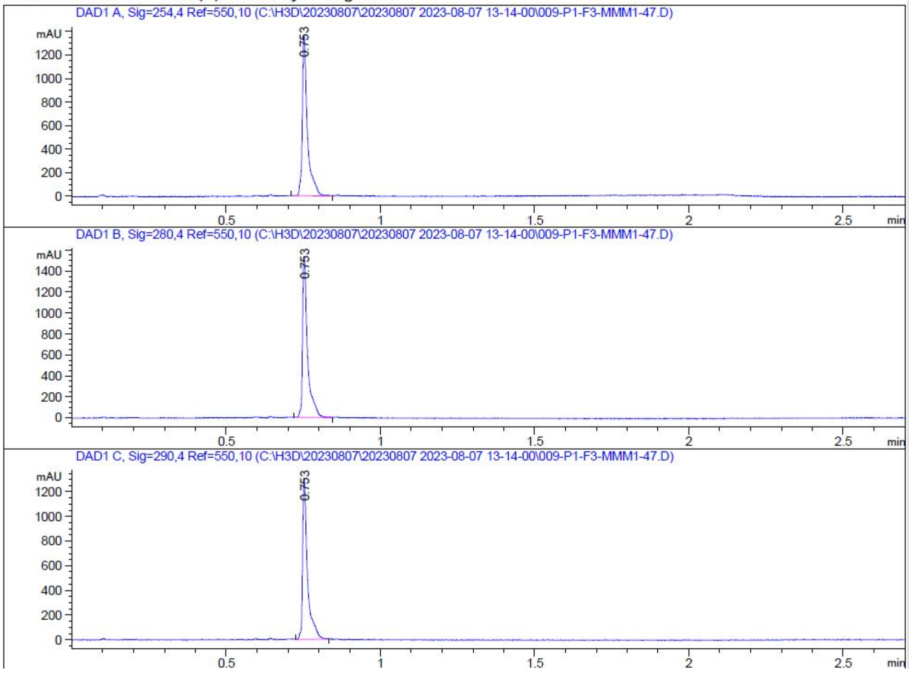

Signal 1: DAD1 A, Sig=254,4 Ref=550,10

Peak #	RetTime [min]	Type	Width [min]	Area [mAU*s]	Height [mAU]	Area %
1	0.753	BB	0.0164	1543.78137	1347.65747	100.0000

Totals : 1543.78137 1347.65747

Signal 2: DAD1 B, Sig=280,4 Ref=550,10

Peak #	RetTime [min]	Type	Width [min]	Area [mAU*s]	Height [mAU]	Area %
1	0.753	BB	0.0160	1711.33130	1530.14478	100.0000

Totals : 1711.33130 1530.14478

Signal 3: DAD1 C, Sig=290,4 Ref=550,10

Peak #	RetTime [min]	Type	Width [min]	Area [mAU*s]	Height [mAU]	Area %
1	0.753	BB	0.0163	1423.87488	1300.59290	100.0000

Totals : 1423.87488 1300.59290

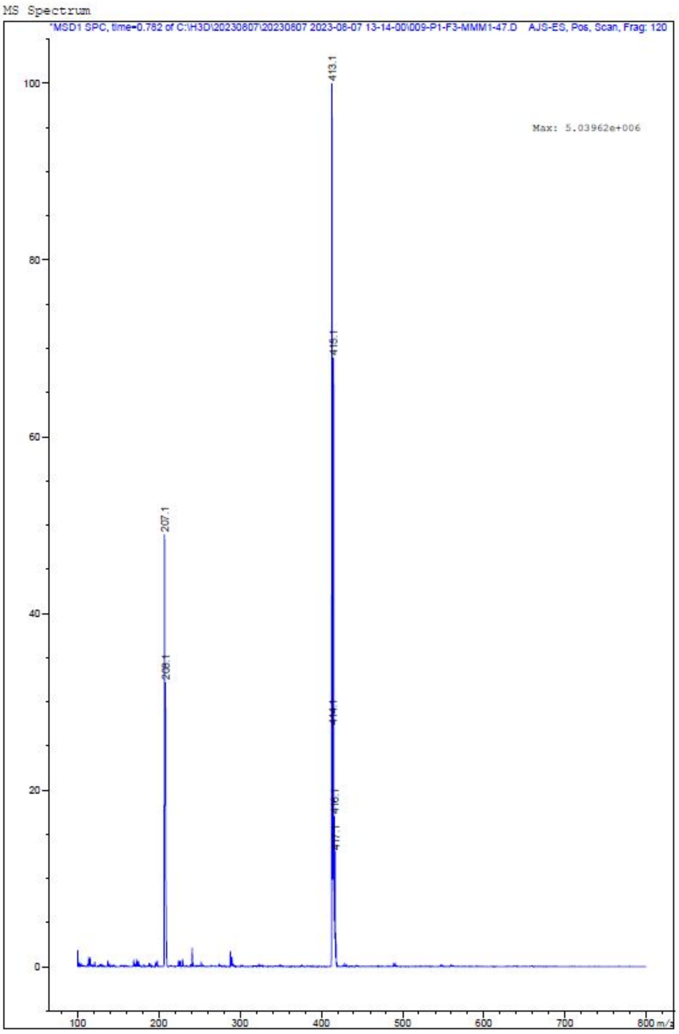

HPLC_UV and MS spectra of **10**

Additional Info : Peak(s) manually integrated

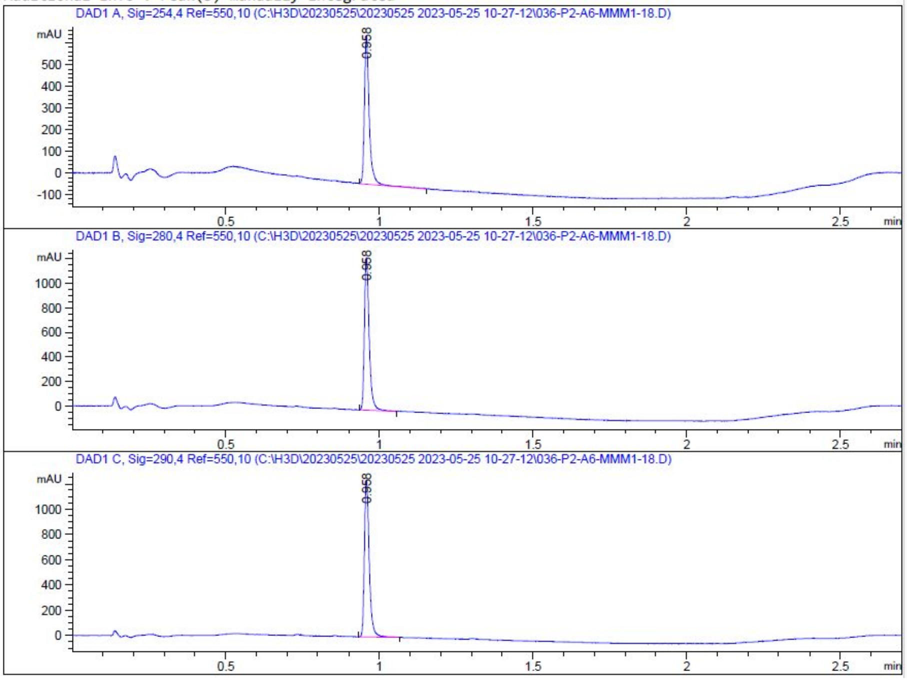

Signal 1: DAD1 A, Sig=254,4 Ref=550,10

Peak #	RetTime [min]	Type	Width [min]	Area [mAU*s]	Height [mAU]	Area %
1	0.958	BB	0.0169	754.81000	682.36603	100.0000
Totals :				754.81000	682.36603	

Signal 2: DAD1 B, Sig=280,4 Ref=550,10

Peak #	RetTime [min]	Type	Width [min]	Area [mAU*s]	Height [mAU]	Area %
1	0.958	BB	0.0168	1360.17004	1236.14380	100.0000
Totals :				1360.17004	1236.14380	

Signal 3: DAD1 C, Sig=290,4 Ref=550,10

Peak #	RetTime [min]	Type	Width [min]	Area [mAU*s]	Height [mAU]	Area %
1	0.958	BB	0.0169	1371.67029	1236.77393	100.0000
Totals :				1371.67029	1236.77393	

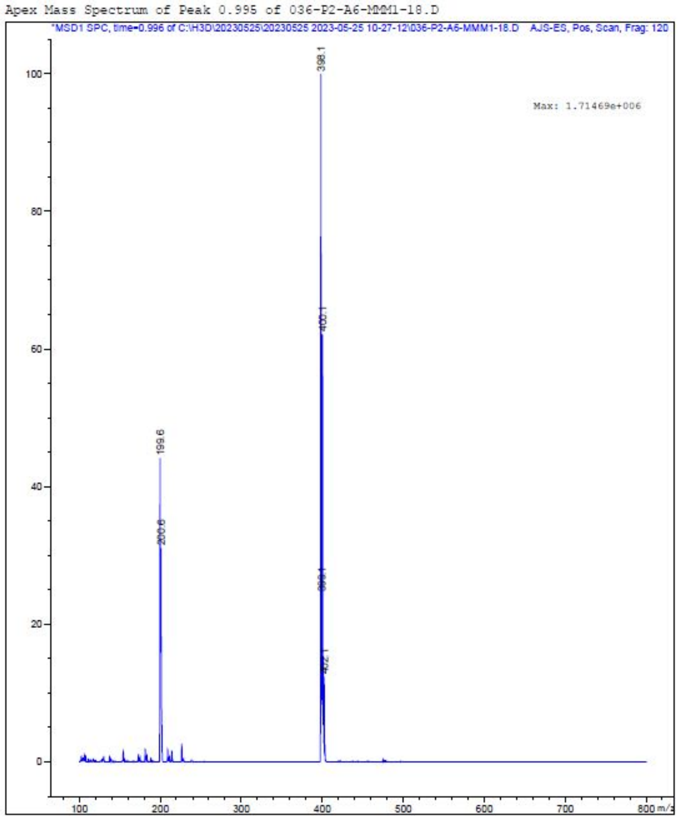

HPLC_UV and MS spectra of **12**

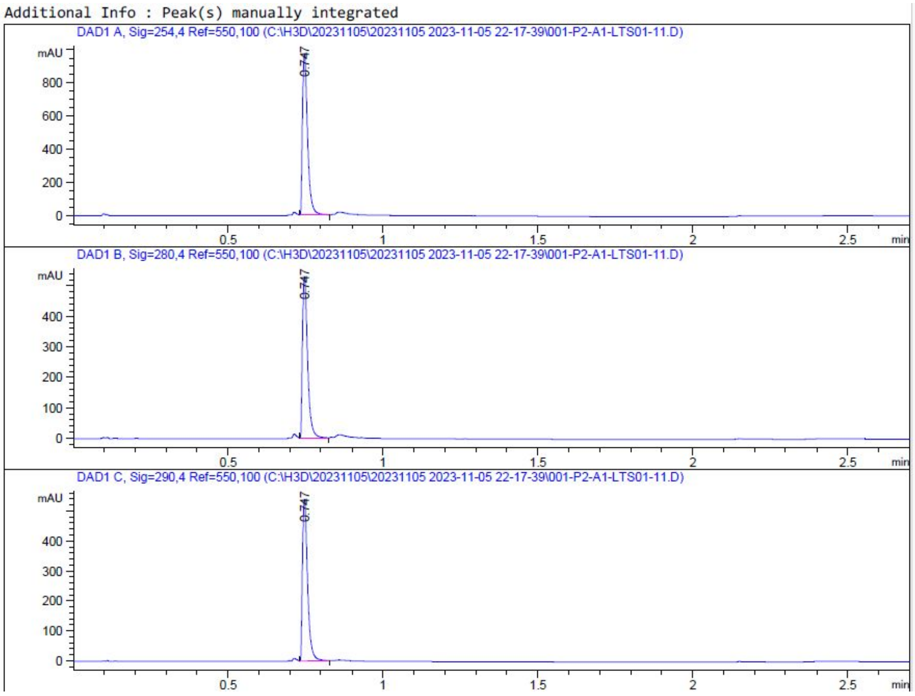

Signal 1: DAD1 A, Sig=254,4 Ref=550,100

Peak #	RetTime [min]	Type	Width [min]	Area [mAU*s]	Height [mAU]	Area %
1	0.747	BB	0.0179	1112.49731	968.74243	100.0000
Totals :				1112.49731	968.74243	

Signal 2: DAD1 B, Sig=280,4 Ref=550,100

Peak #	RetTime [min]	Type	Width [min]	Area [mAU*s]	Height [mAU]	Area %
1	0.747	BB	0.0179	608.07068	528.10980	100.0000
Totals :				608.07068	528.10980	

Signal 3: DAD1 C, Sig=290,4 Ref=550,100

Peak #	RetTime [min]	Type	Width [min]	Area [mAU*s]	Height [mAU]	Area %
1	0.747	BB	0.0179	616.38727	536.79327	100.0000
Totals :				616.38727	536.79327	

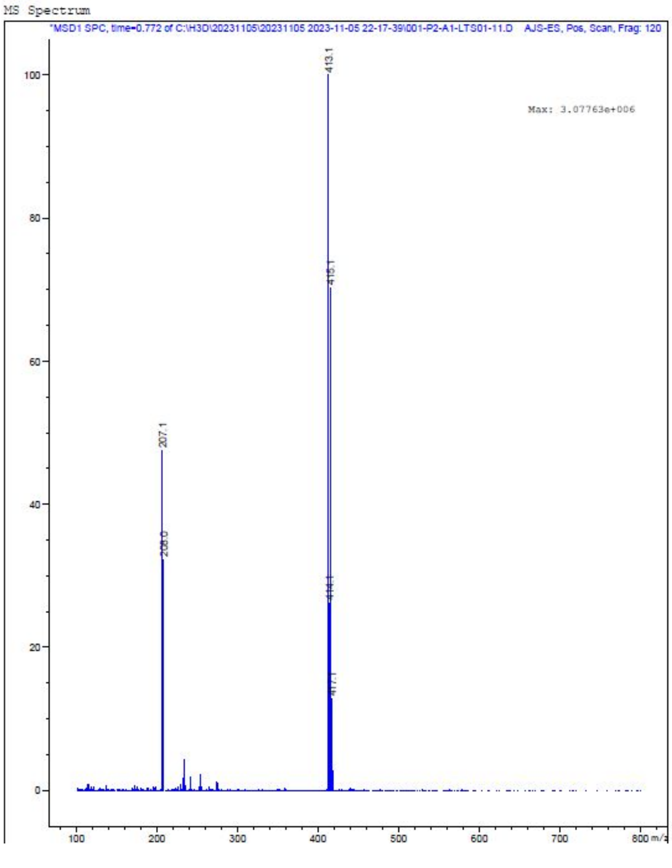

HPLC_UV and MS spectra of **13**

Additional Info : Peak(s) manually integrated

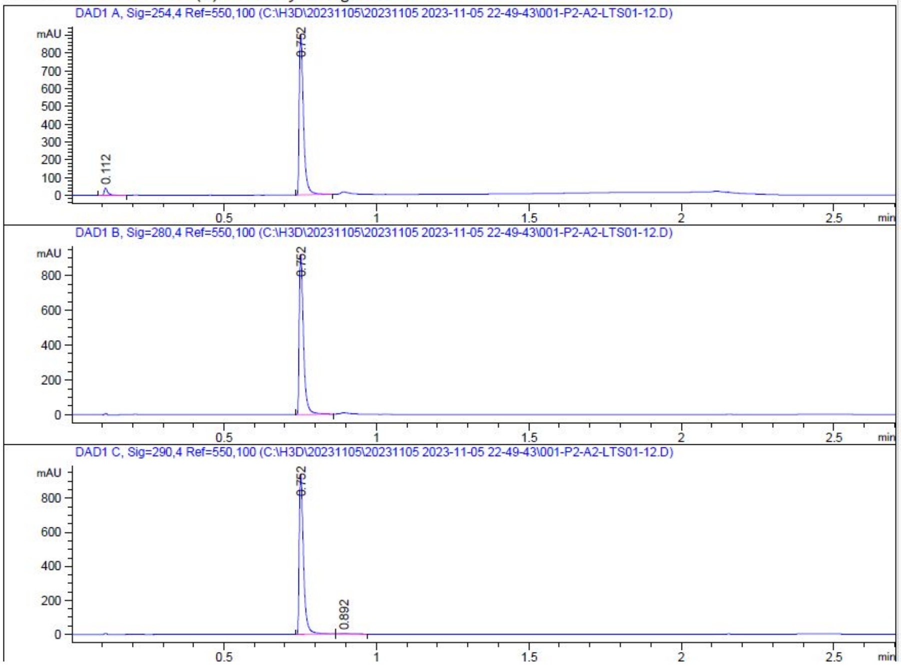

Signal 1: DAD1 A, Sig=254,4 Ref=550,100

Peak #	RetTime [min]	Type	Width [min]	Area [mAU*s]	Height [mAU]	Area %
1	0.112	BB	0.0122	33.62469	40.55139	3.7225
2	0.752	BB	0.0153	869.65649	894.60345	96.2775

Totals : 903.28119 935.15485

Signal 2: DAD1 B, Sig=280,4 Ref=550,100

Peak #	RetTime [min]	Type	Width [min]	Area [mAU*s]	Height [mAU]	Area %
1	0.752	BB	0.0153	887.58368	914.92169	100.0000

Totals : 887.58368 914.92169

Signal 3: DAD1 C, Sig=290,4 Ref=550,100

Peak #	RetTime [min]	Type	Width [min]	Area [mAU*s]	Height [mAU]	Area %
1	0.752	BB	0.0153	906.31836	934.93439	99.3407
2	0.892	BB	0.0299	6.01536	2.99638	0.6593

Totals : 912.33372 937.93077

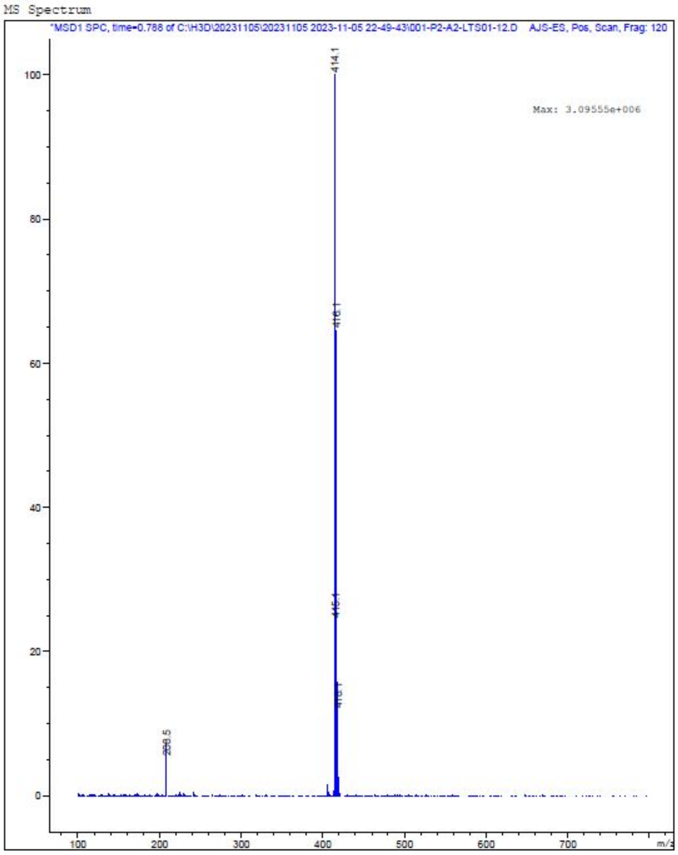

HPLC_UV and MS spectra of **17**

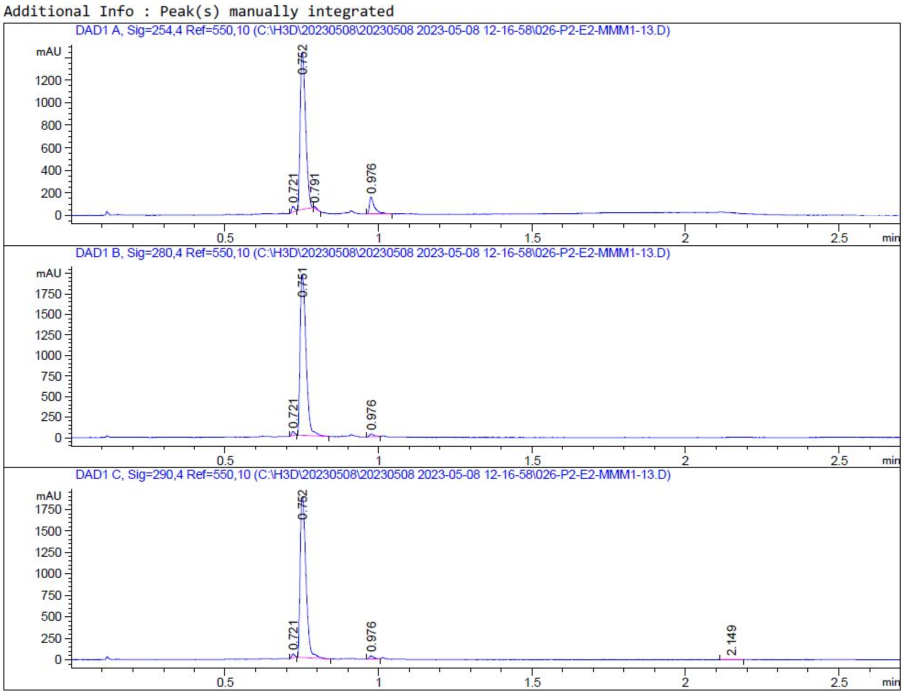

Signal 1: DAD1 A, Sig=254,4 Ref=550,10

Peak #	RetTime [min]	Type	Width [min]	Area [mAU*s]	Height [mAU]	Area %
1	0.721	BB	0.0104	33.42461	49.35742	1.7283
2	0.752	BB	0.0194	1712.96399	1389.83508	88.5706
3	0.791	BB	0.0130	12.08730	15.75022	0.6250
4	0.976	BB	0.0166	175.53304	150.68704	9.0761
Totals :				1934.00893	1605.62977	

Signal 2: DAD1 B, Sig=280,4 Ref=550,10

Peak #	RetTime [min]	Type	Width [min]	Area [mAU*s]	Height [mAU]	Area %
1	0.721	BB	0.0107	34.22047	48.97015	1.2710
2	0.751	BB	0.0213	2623.77222	1931.29956	97.4504
3	0.976	BB	0.0142	34.42404	35.87925	1.2786
Totals :				2692.41673	2016.14896	

Signal 3: DAD1 C, Sig=290,4 Ref=550,10

Peak #	RetTime [min]	Type	Width [min]	Area [mAU*s]	Height [mAU]	Area %
1	0.721	BB	0.0106	31.01176	44.74818	1.2247
2	0.752	BB	0.0209	2458.02148	1865.59265	97.0690
3	0.976	BB	0.0146	37.33307	37.63632	1.4743
4	2.149	BB	0.0269	5.87440	2.80429	0.2320
Totals :				2532.24072	1950.78145	

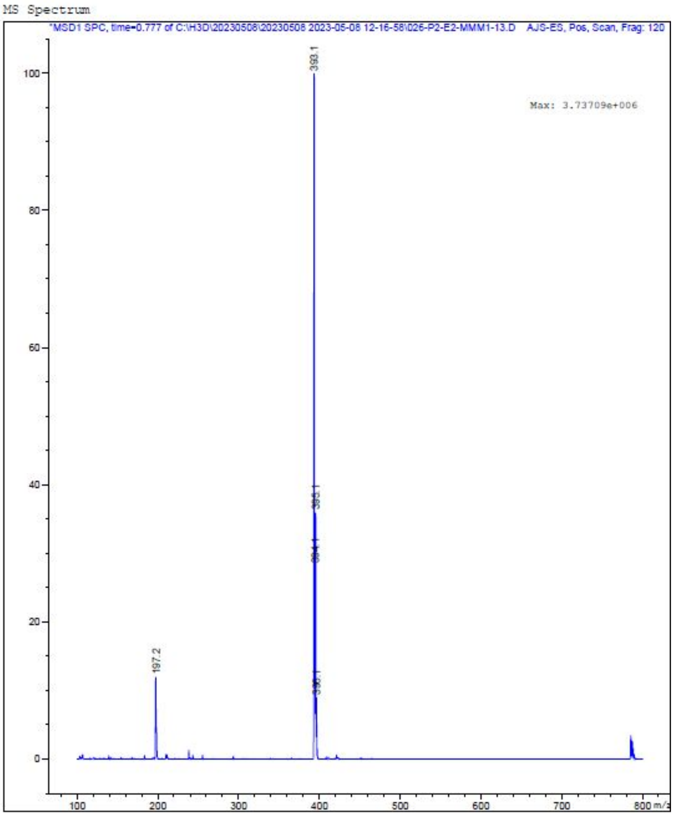

HPLC_UV and MS spectra of **18**

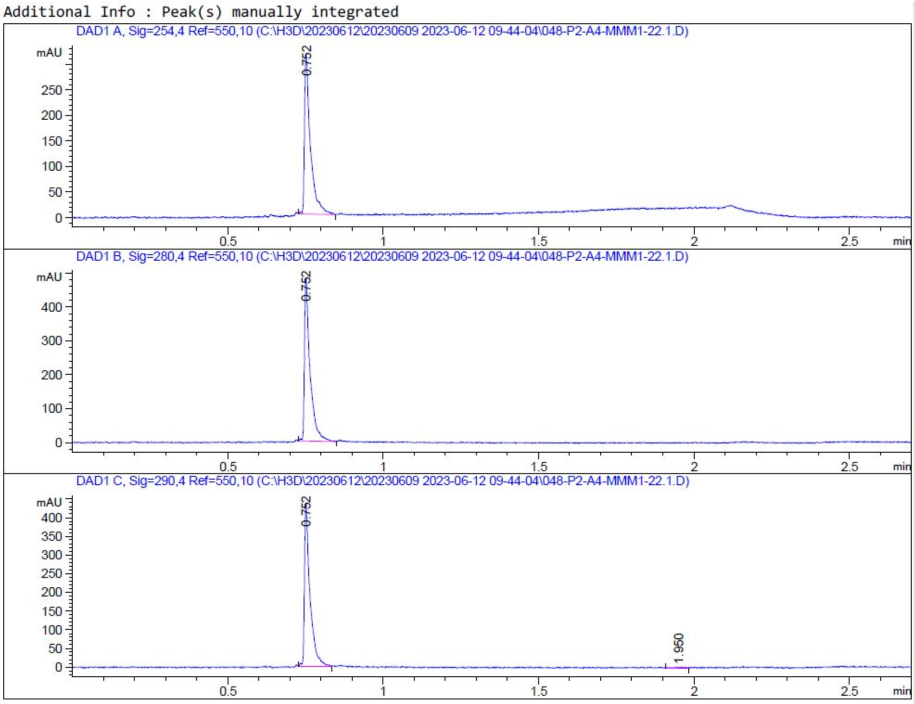

Signal 1: DAD1 A, Sig=254,4 Ref=550,10

Peak #	RetTime [min]	Type	Width [min]	Area [mAU*s]	Height [mAU]	Area %
1	0.752	BB	0.0187	419.55670	312.58749	100.0000
Totals :				419.55670	312.58749	

Signal 2: DAD1 B, Sig=280,4 Ref=550,10

Peak #	RetTime [min]	Type	Width [min]	Area [mAU*s]	Height [mAU]	Area %
1	0.752	BB	0.0178	603.50745	475.83493	100.0000
Totals :				603.50745	475.83493	

Signal 3: DAD1 C, Sig=290,4 Ref=550,10

Peak #	RetTime [min]	Type	Width [min]	Area [mAU*s]	Height [mAU]	Area %
1	0.752	BB	0.0176	540.06036	431.80829	98.8672
2	1.950	BB	0.0290	6.18808	2.58166	1.1328
Totals :				546.24844	434.38995	

MS Spectrum

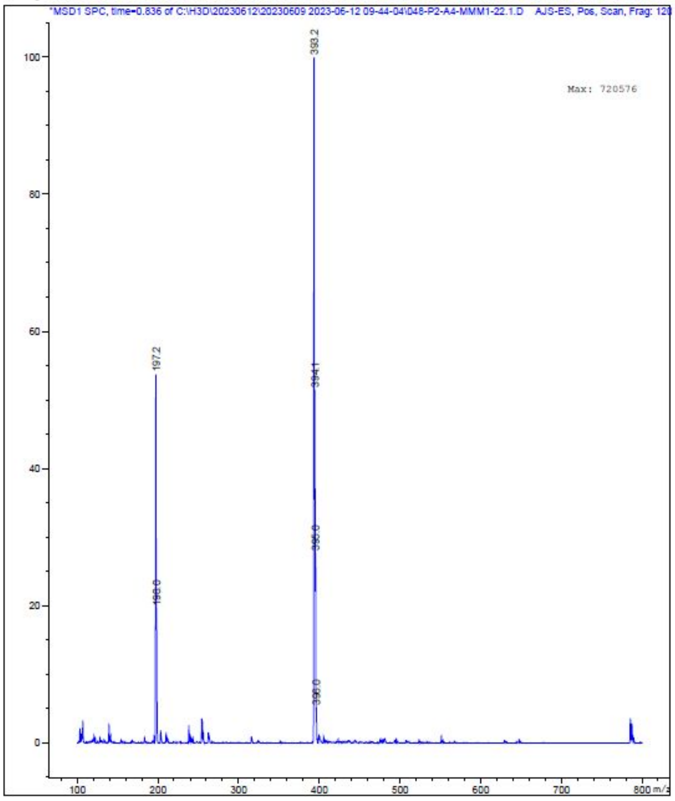

HPLC_UV and MS spectra of **19**

Additional Info : Peak(s) manually integrated

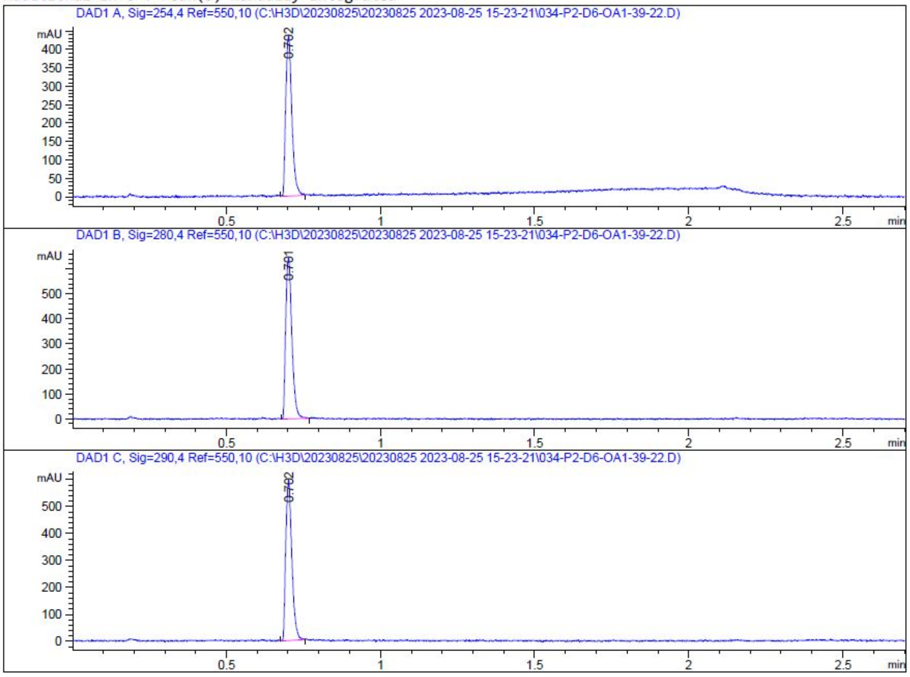

Signal 1: DAD1 A, Sig=254,4 Ref=550,10

Peak #	RetTime [min]	Type	Width [min]	Area [mAU*s]	Height [mAU]	Area %
1	0.702	BB	0.0215	593.88043	434.08969	100.0000
Totals :				593.88043	434.08969	

Signal 2: DAD1 B, Sig=280,4 Ref=550,10

Peak #	RetTime [min]	Type	Width [min]	Area [mAU*s]	Height [mAU]	Area %
1	0.701	BB	0.0221	891.07727	643.89551	100.0000
Totals :				891.07727	643.89551	

Signal 3: DAD1 C, Sig=290,4 Ref=550,10

Peak #	RetTime [min]	Type	Width [min]	Area [mAU*s]	Height [mAU]	Area %
1	0.702	BB	0.0208	807.37573	595.92920	100.0000
Totals :				807.37573	595.92920	

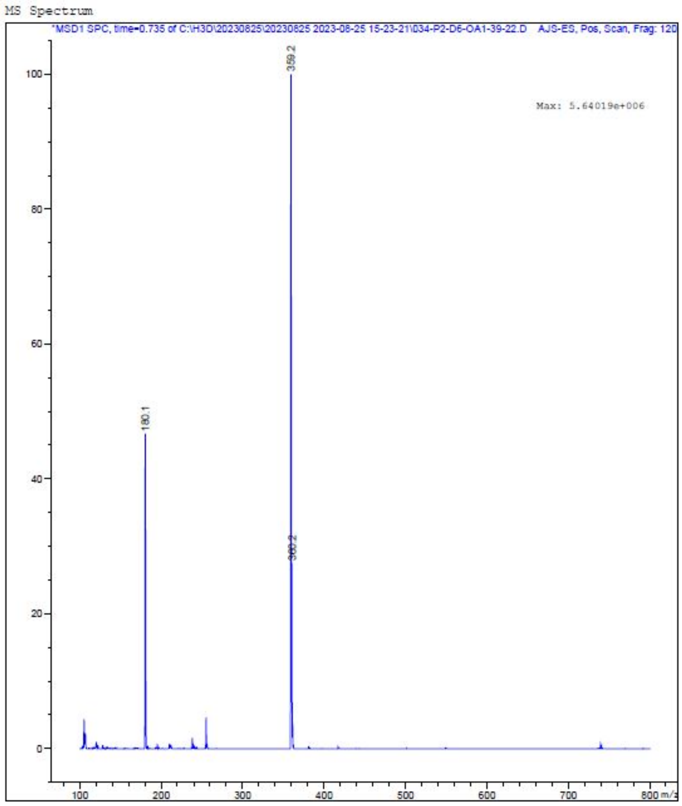

HPLC_UV and MS spectra of **20**

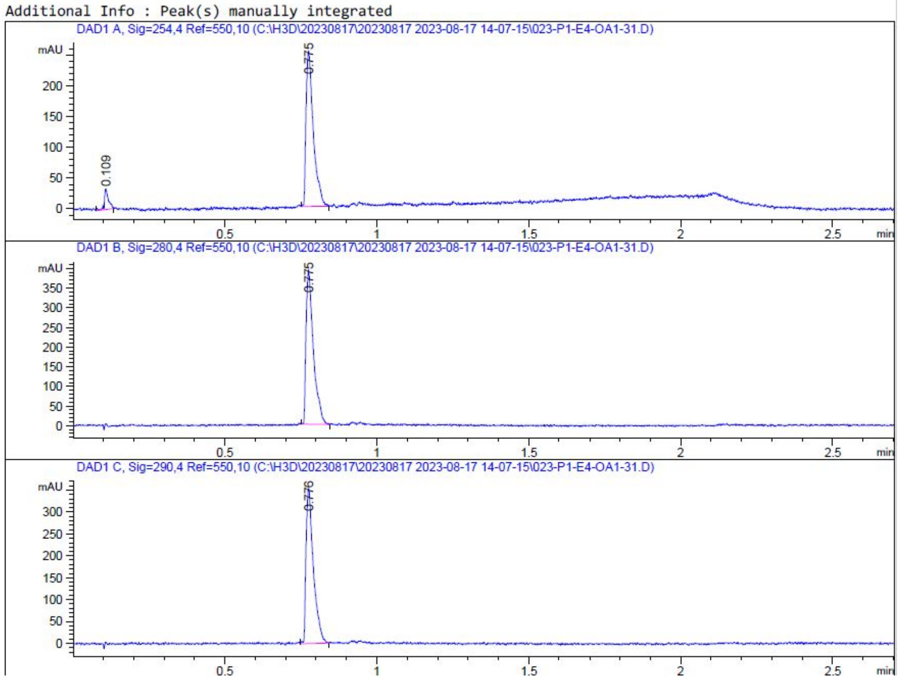

Signal 1: DAD1 A, Sig=254,4 Ref=550,10

Peak #	RetTime [min]	Type	Width [min]	Area [mAU*s]	Height [mAU]	Area %
1	0.109	BB	0.0132	31.54293	32.95142	6.9586
2	0.775	BB	0.0250	421.75232	252.12062	93.0414
Totals :				453.29525	285.07204	

Signal 2: DAD1 B, Sig=280,4 Ref=550,10

Peak #	RetTime [min]	Type	Width [min]	Area [mAU*s]	Height [mAU]	Area %
1	0.775	BB	0.0246	653.46924	389.90747	100.0000
Totals :				653.46924	389.90747	

Signal 3: DAD1 C, Sig=290,4 Ref=550,10

Peak #	RetTime [min]	Type	Width [min]	Area [mAU*s]	Height [mAU]	Area %
1	0.776	BB	0.0246	595.31573	354.16681	100.0000
Totals :				595.31573	354.16681	

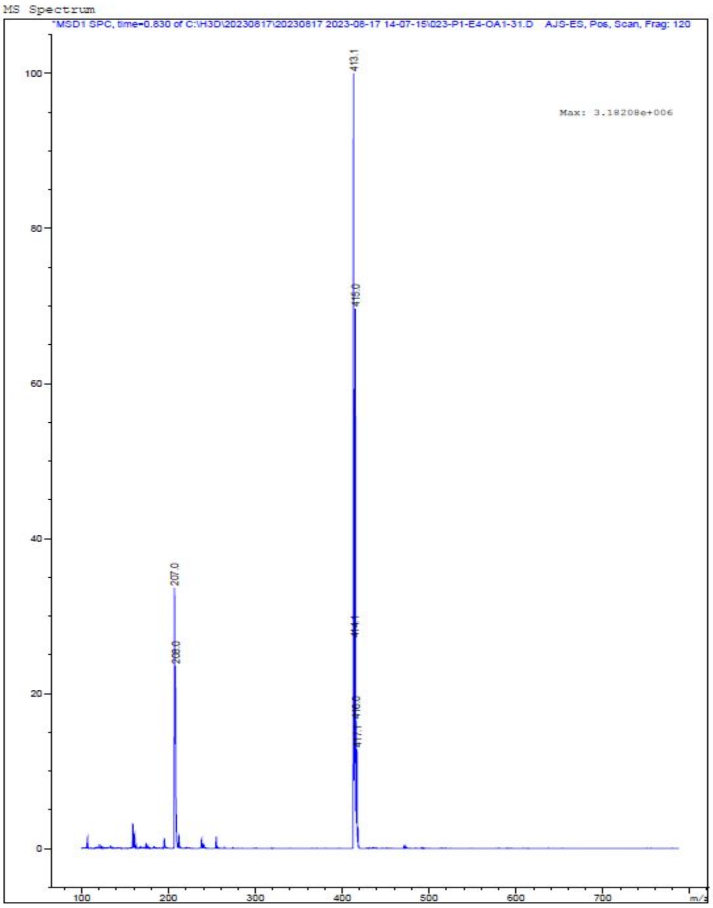

HPLC_UV and MS spectra of **21**

Additional Info : Peak(s) manually integrated

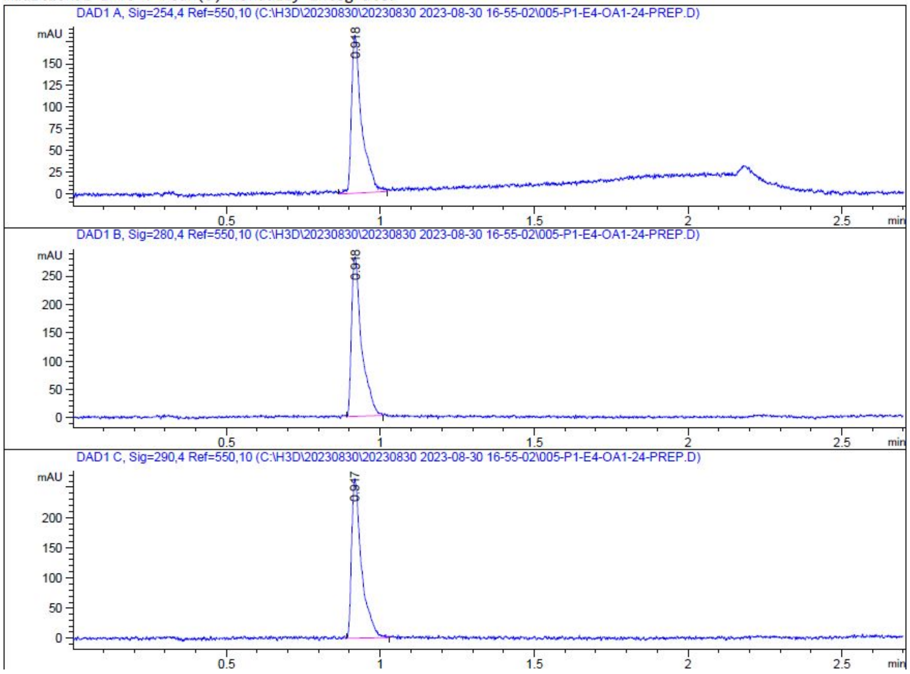

Signal 1: DAD1 A, Sig=254,4 Ref=550,10

Peak #	RetTime [min]	Type	Width [min]	Area [mAU*s]	Height [mAU]	Area %
1	0.918	BB	0.0331	422.43292	181.57036	100.0000
Totals :				422.43292	181.57036	

Signal 2: DAD1 B, Sig=280,4 Ref=550,10

Peak #	RetTime [min]	Type	Width [min]	Area [mAU*s]	Height [mAU]	Area %
1	0.918	BB	0.0332	640.64990	280.28595	100.0000
Totals :				640.64990	280.28595	

Signal 3: DAD1 C, Sig=290,4 Ref=550,10

Peak #	RetTime [min]	Type	Width [min]	Area [mAU*s]	Height [mAU]	Area %
1	0.917	BB	0.0328	593.04608	263.45978	100.0000
Totals :				593.04608	263.45978	

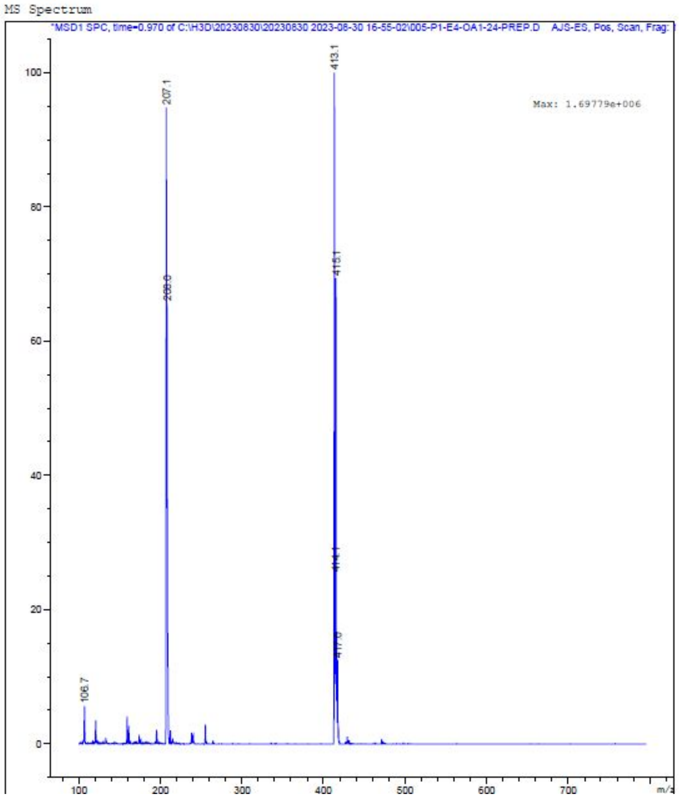

HPLC_UV and MS spectra of **22**

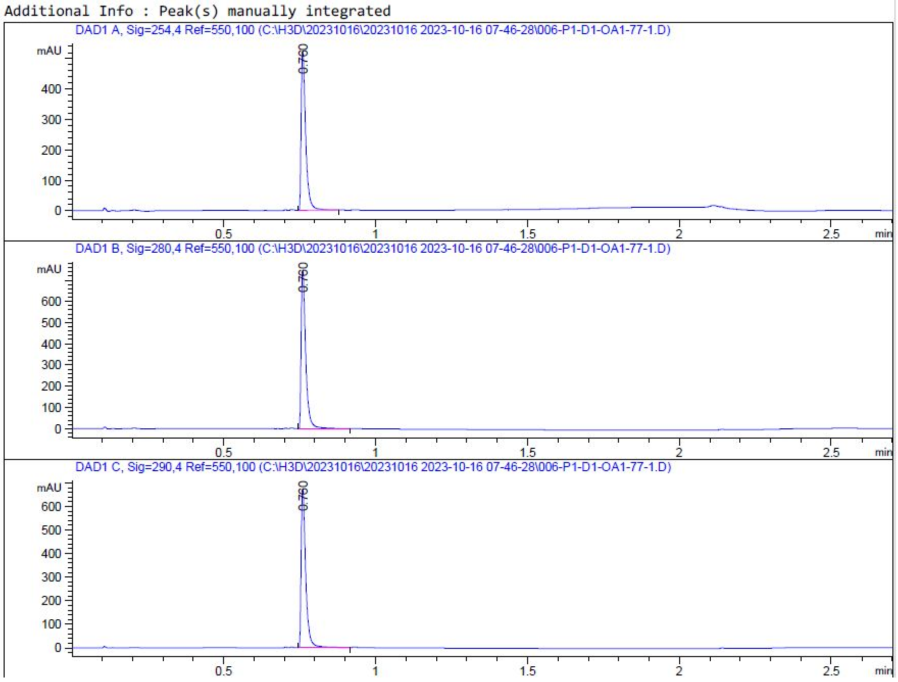

Signal 1: DAD1 A, Sig=254,4 Ref=550,100

Peak #	RetTime [min]	Type	Width [min]	Area [mAU*s]	Height [mAU]	Area %
1	0.760	BB	0.0163	547.49402	519.69495	100.0000
Totals :				547.49402	519.69495	

Signal 2: DAD1 B, Sig=280,4 Ref=550,100

Peak #	RetTime [min]	Type	Width [min]	Area [mAU*s]	Height [mAU]	Area %
1	0.760	BB	0.0164	790.39636	744.60236	100.0000
Totals :				790.39636	744.60236	

Signal 3: DAD1 C, Sig=290,4 Ref=550,100

Peak #	RetTime [min]	Type	Width [min]	Area [mAU*s]	Height [mAU]	Area %
1	0.760	BB	0.0164	713.48956	671.42969	100.0000
Totals :				713.48956	671.42969	

Apex Mass Spectrum of Peak 0.782 of 006-P1-D1-OA1-77-1.D

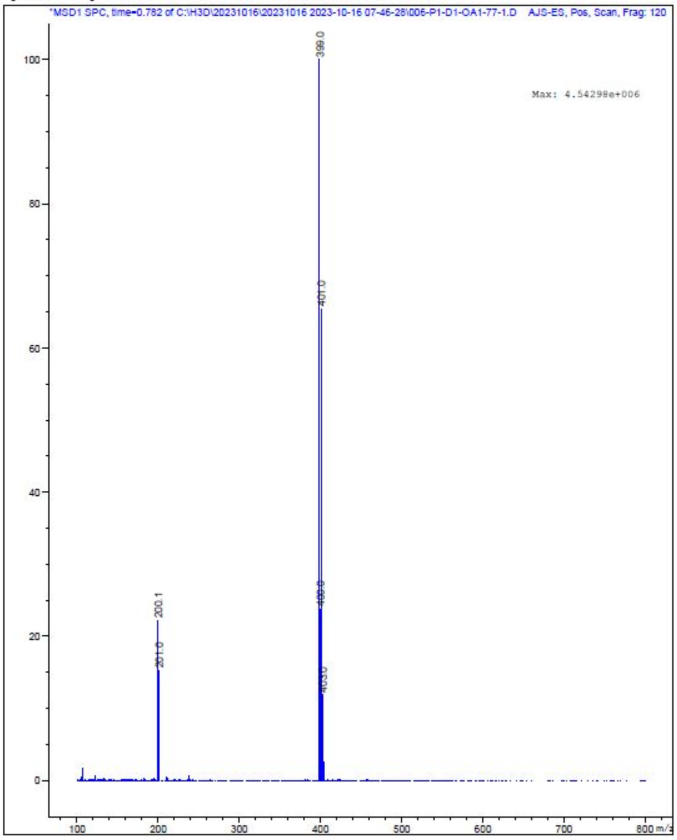

HPLC_UV and MS spectra of **23**

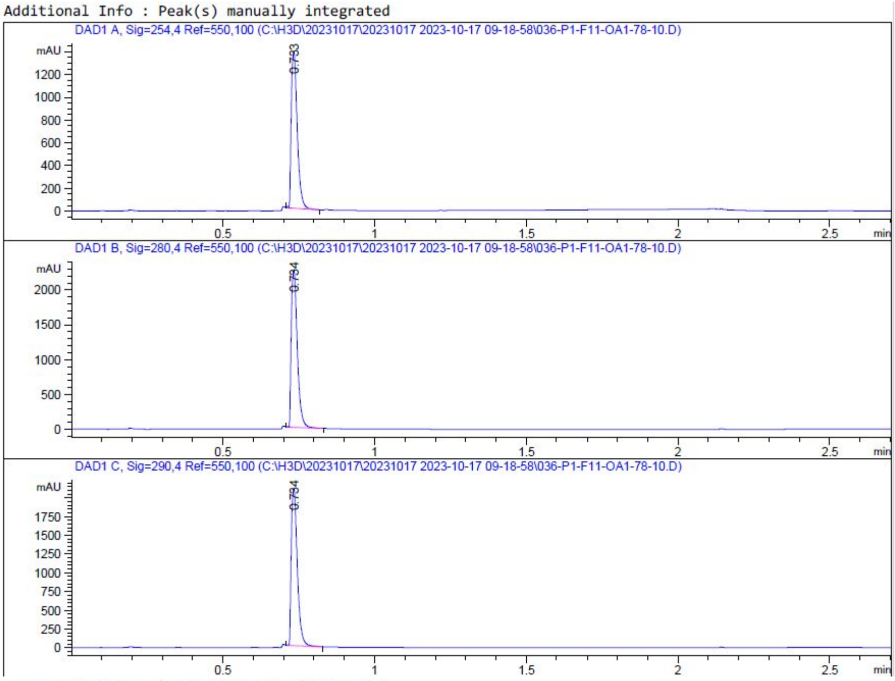

Signal 1: DAD1 A, Sig=254,4 Ref=550,100

Peak #	RetTime [min]	Type	Width [min]	Area [mAU*s]	Height [mAU]	Area %
1	0.733	BB	0.0213	1869.28943	1378.17993	100.0000
Totals :				1869.28943	1378.17993	

Signal 2: DAD1 B, Sig=280,4 Ref=550,100

Peak #	RetTime [min]	Type	Width [min]	Area [mAU*s]	Height [mAU]	Area %
1	0.734	BB	0.0214	3091.72363	2263.11890	100.0000
Totals :				3091.72363	2263.11890	

Signal 3: DAD1 C, Sig=290,4 Ref=550,100

Peak #	RetTime [min]	Type	Width [min]	Area [mAU*s]	Height [mAU]	Area %
1	0.734	BB	0.0215	2877.05347	2104.36792	100.0000
Totals :				2877.05347	2104.36792	

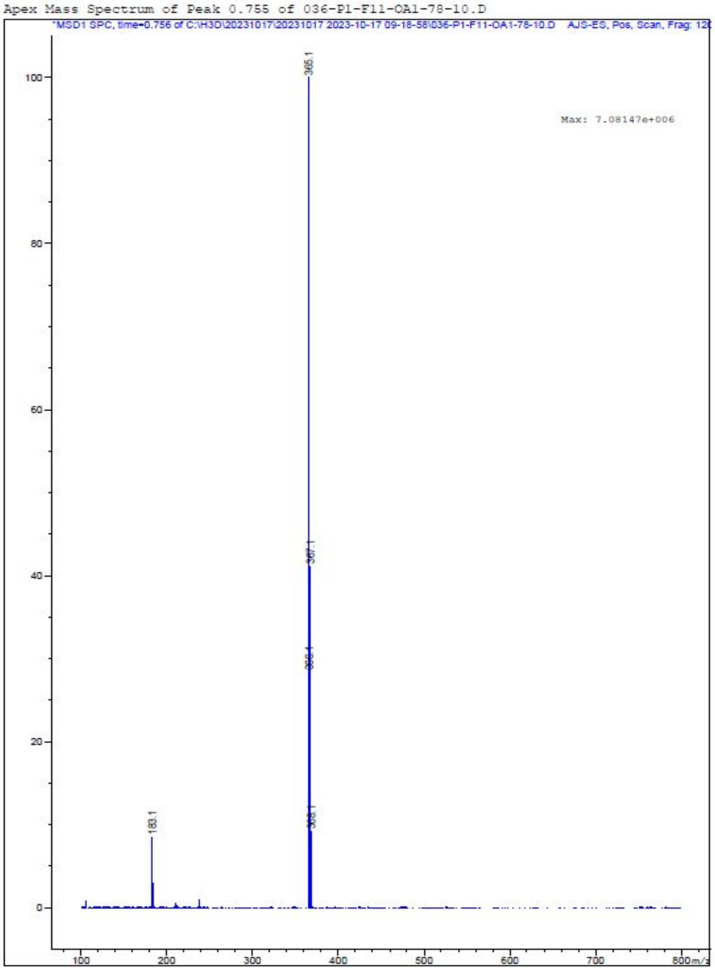

HPLC_UV and MS spectra of **24**

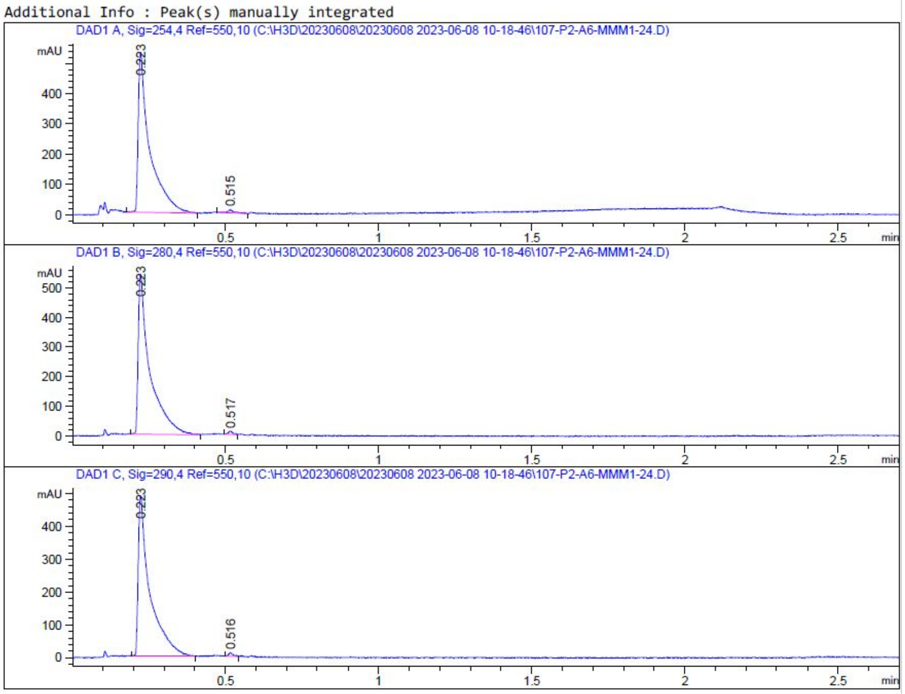

Signal 1: DAD1 A, Sig=254,4 Ref=550,10

Peak #	RetTime [min]	Type	Width [min]	Area [mAU*s]	Height [mAU]	Area %
1	0.223	BB	0.0346	1385.77380	528.02283	98.6964
2	0.515	BB	0.0244	18.30391	9.96585	1.3036
Totals :				1404.07772	537.98868	

Signal 2: DAD1 B, Sig=280,4 Ref=550,10

Peak #	RetTime [min]	Type	Width [min]	Area [mAU*s]	Height [mAU]	Area %
1	0.223	BB	0.0358	1427.32739	539.77966	99.1946
2	0.517	BB	0.0160	11.58893	10.77389	0.8054
Totals :				1438.91632	550.55355	

Signal 3: DAD1 C, Sig=290,4 Ref=550,10

Peak #	RetTime [min]	Type	Width [min]	Area [mAU*s]	Height [mAU]	Area %
1	0.223	BB	0.0349	1295.88794	488.58636	99.1918
2	0.516	BB	0.0133	10.55885	10.90593	0.8082
Totals :				1306.44679	499.49229	

MS Spectrum

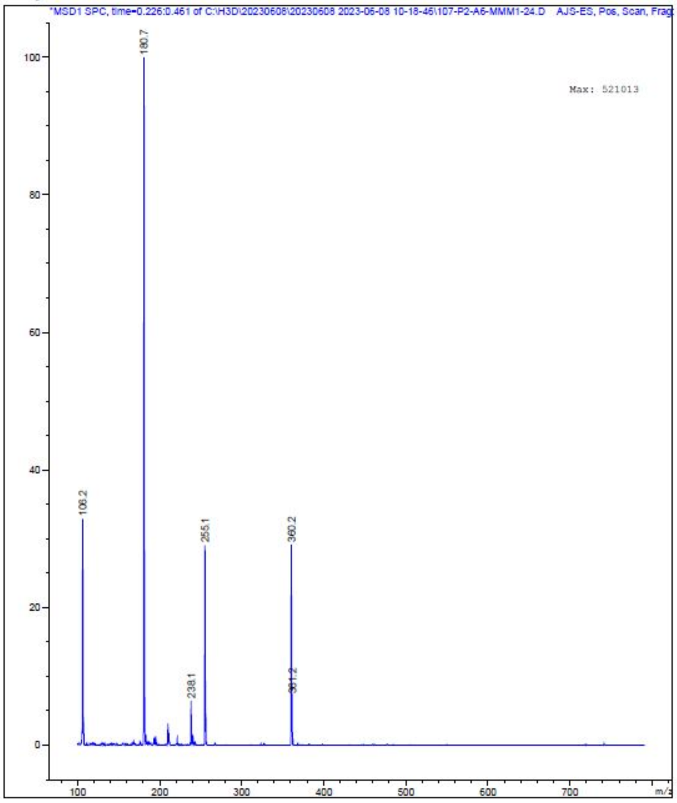

HPLC_UV and MS spectra of **25**

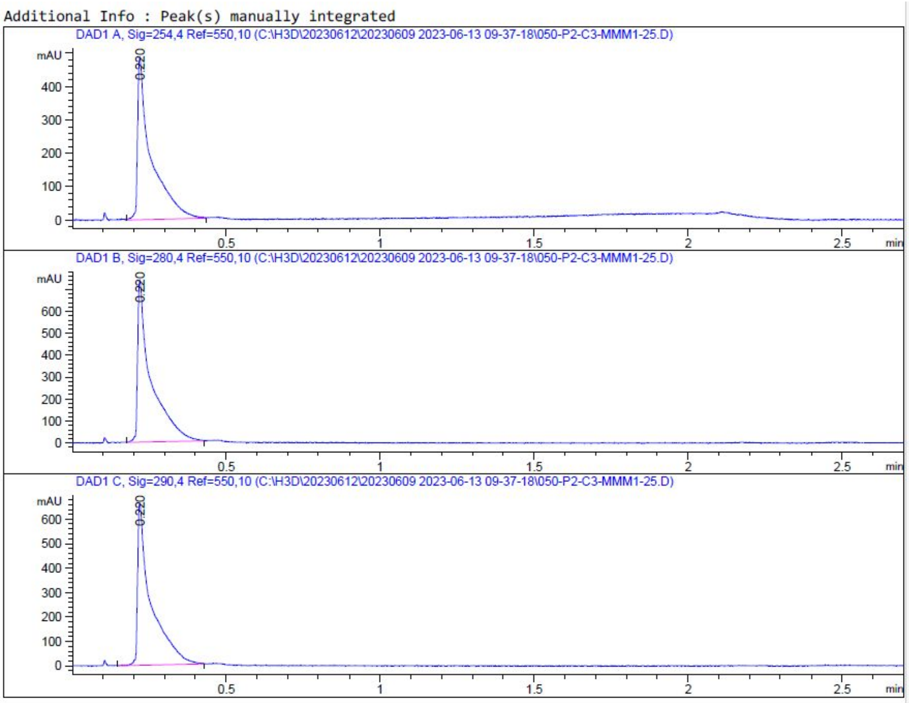

Signal 1: DAD1 A, Sig=254,4 Ref=550,10

Peak #	RetTime [min]	Type	Width [min]	Area [mAU*s]	Height [mAU]	Area %
1	0.220	BB	0.0400	1479.34180	486.85257	100.0000

Totals : 1479.34180 486.85257

Signal 2: DAD1 B, Sig=280,4 Ref=550,10

Peak #	RetTime [min]	Type	Width [min]	Area [mAU*s]	Height [mAU]	Area %
1	0.220	BB	0.0396	2225.97314	740.10754	100.0000

Totals : 2225.97314 740.10754

Signal 3: DAD1 C, Sig=290,4 Ref=550,10

Peak #	RetTime [min]	Type	Width [min]	Area [mAU*s]	Height [mAU]	Area %
1	0.220	BB	0.0398	2006.34888	663.94653	100.0000

Totals : 2006.34888 663.94653

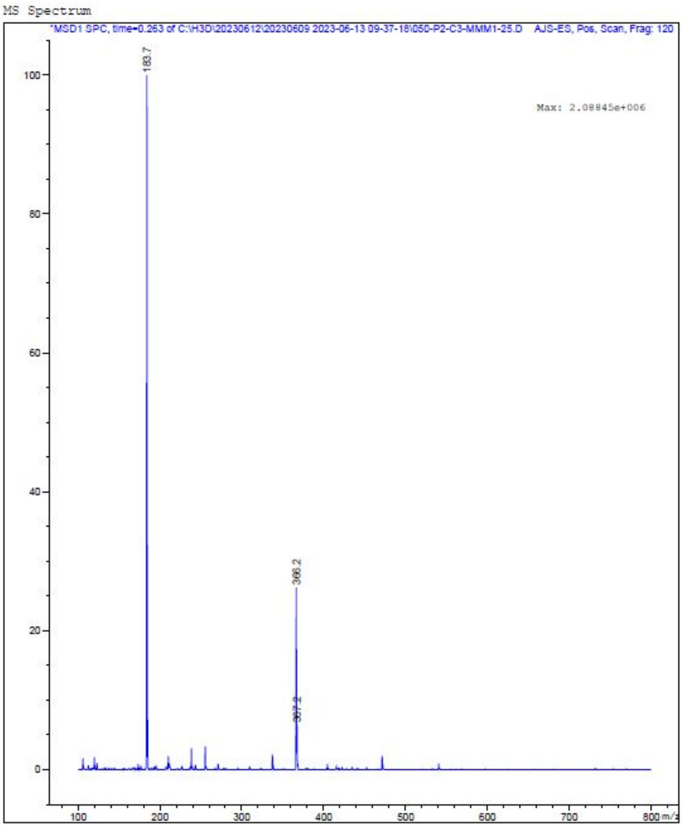

HPLC_UV and MS spectra of **26**

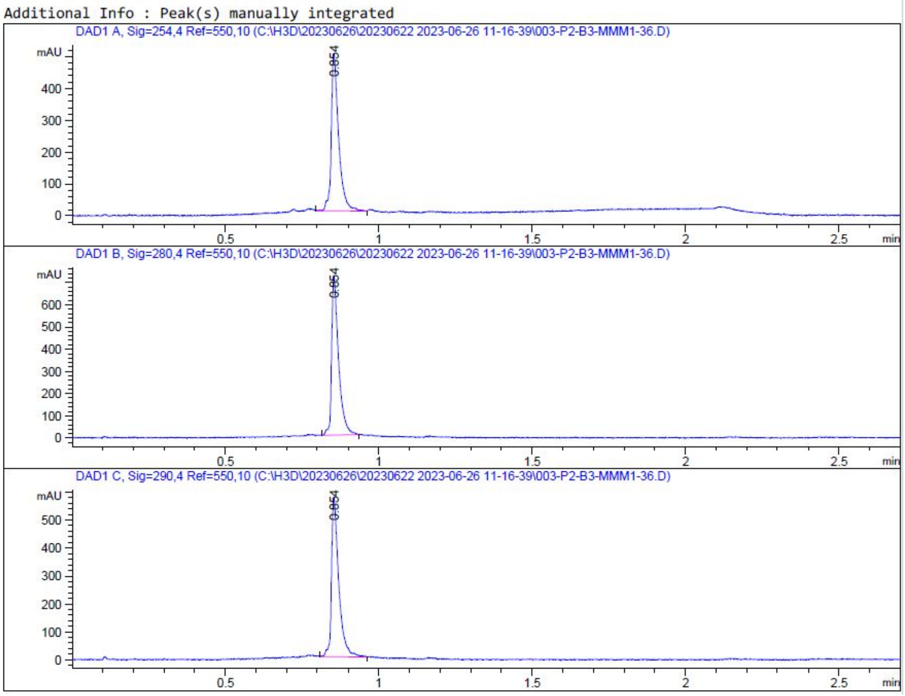

Signal 1: DAD1 A, Sig=254,4 Ref=550,10

Peak #	RetTime [min]	Type	Width [min]	Area [mAU*s]	Height [mAU]	Area %
1	0.854	BB	0.0246	829.05493	493.25574	100.0000

Totals : 829.05493 493.25574

Signal 2: DAD1 B, Sig=280,4 Ref=550,10

Peak #	RetTime [min]	Type	Width [min]	Area [mAU*s]	Height [mAU]	Area %
1	0.854	BB	0.0239	1129.27441	716.40558	100.0000

Totals : 1129.27441 716.40558

Signal 3: DAD1 C, Sig=290,4 Ref=550,10

Peak #	RetTime [min]	Type	Width [min]	Area [mAU*s]	Height [mAU]	Area %
1	0.854	BB	0.0249	940.82904	567.20984	100.0000

Totals : 940.82904 567.20984

MS Spectrum

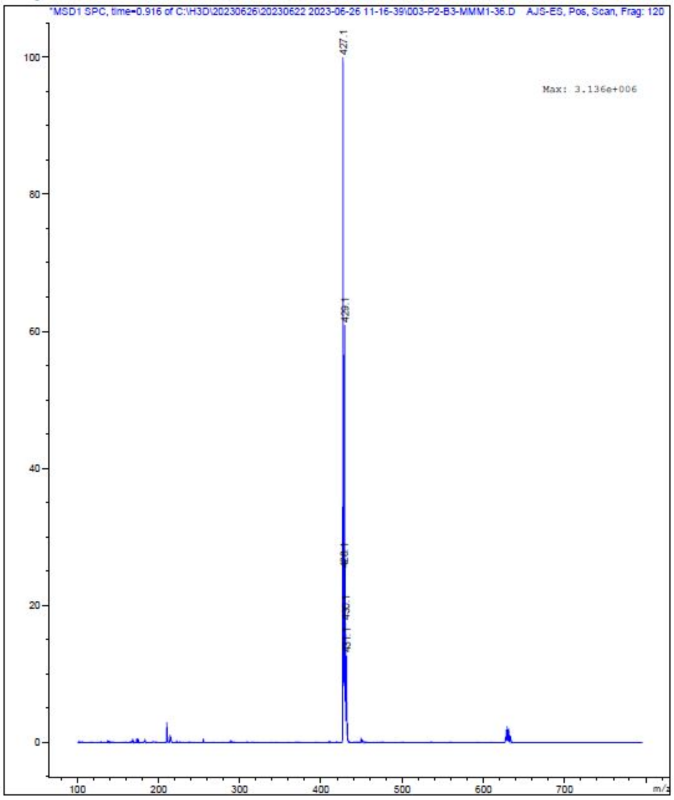

HPLC_UV and MS spectra of **27**

Additional Info : Peak(s) manually integrated

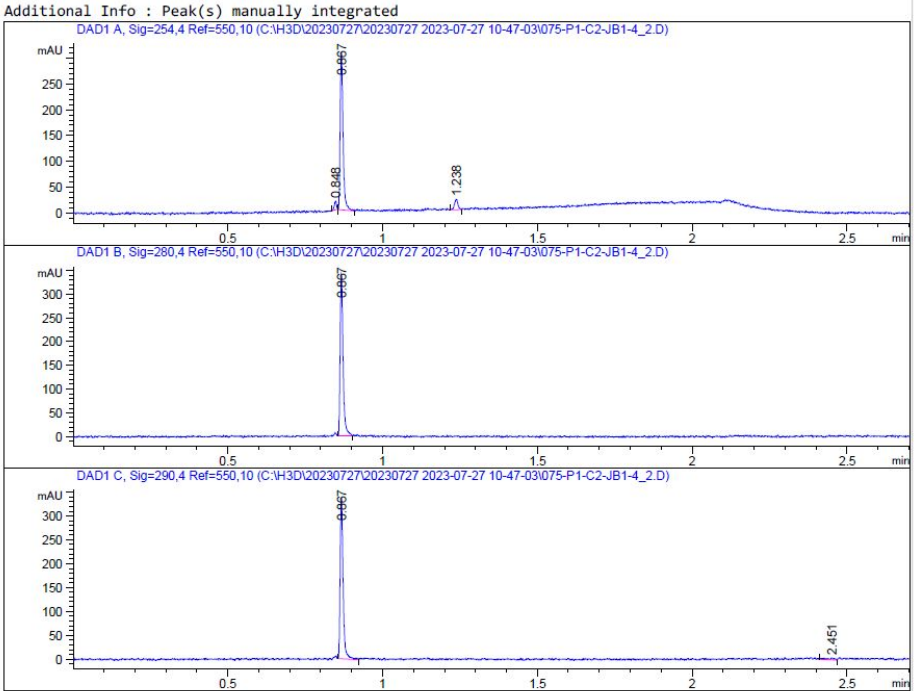

Signal 1: DAD1 A, Sig=254,4 Ref=550,10

Peak #	RetTime [min]	Type	Width [min]	Area [mAU*s]	Height [mAU]	Area %
1	0.848	BB	7.91e-3	8.40706	16.58336	3.8520
2	0.867	BB	9.55e-3	193.51971	299.08743	88.6691
3	1.238	BB	0.0124	16.32264	20.32250	7.4789

Totals : 218.24942 335.99329

Signal 2: DAD1 B, Sig=280,4 Ref=550,10

Peak #	RetTime [min]	Type	Width [min]	Area [mAU*s]	Height [mAU]	Area %
1	0.867	BB	9.64e-3	218.45354	333.61163	100.0000

Totals : 218.45354 333.61163

Signal 3: DAD1 C, Sig=290,4 Ref=550,10

Peak #	RetTime [min]	Type	Width [min]	Area [mAU*s]	Height [mAU]	Area %
1	0.867	BB	9.72e-3	219.81821	332.01355	97.2379
2	2.451	BB	0.0206	6.24414	3.81975	2.7621

Totals : 226.06235 335.83330

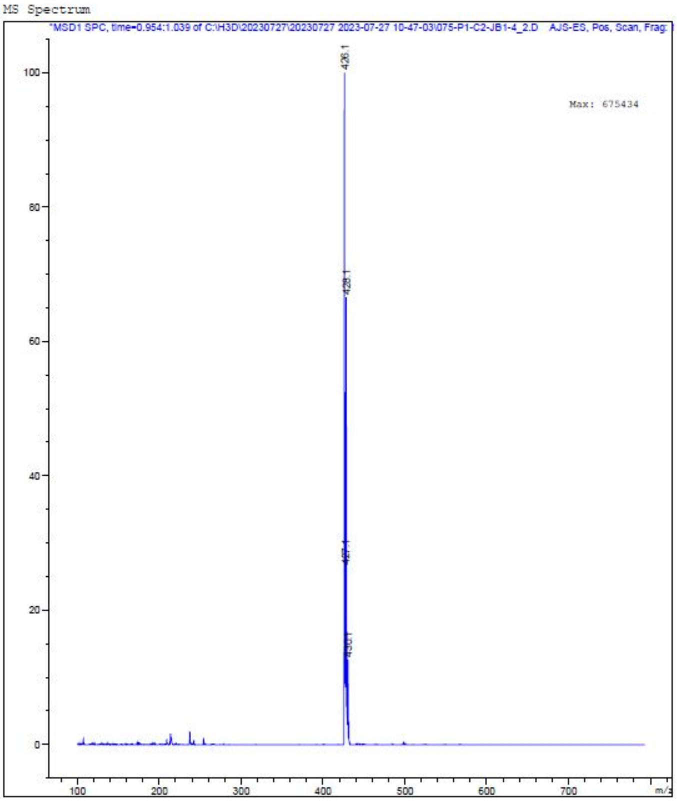

HPLC_UV and MS spectra of **28**

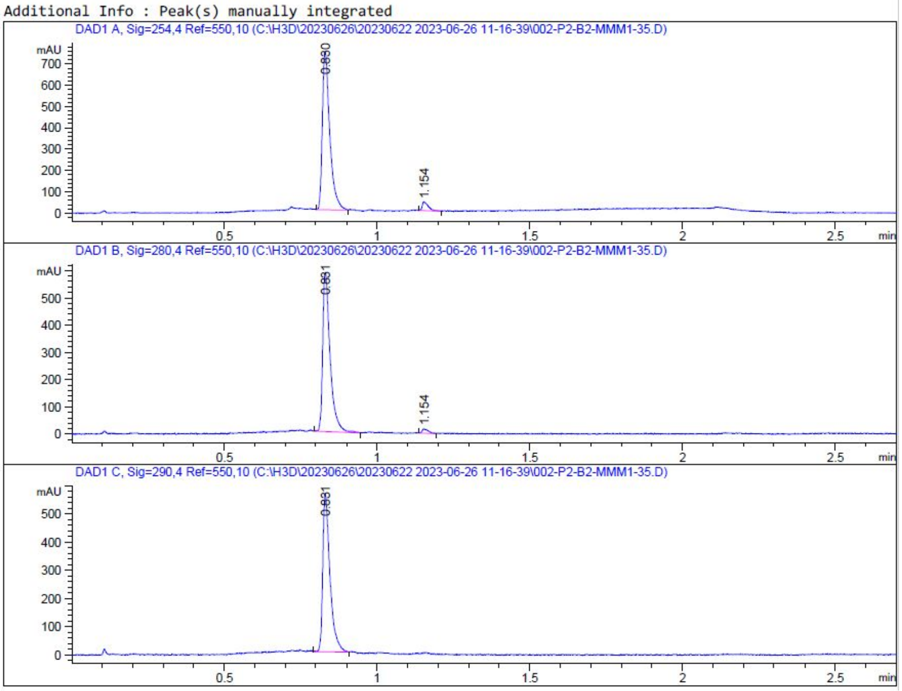

Signal 1: DAD1 A, Sig=254,4 Ref=550,10

Peak #	RetTime [min]	Type	Width [min]	Area [mAU*s]	Height [mAU]	Area %
1	0.830	BB	0.0247	1253.55188	742.29199	95.7293
2	1.154	BB	0.0187	55.92441	40.22633	4.2707
Totals :				1309.47629	782.51833	

Signal 2: DAD1 B, Sig=280,4 Ref=550,10

Peak #	RetTime [min]	Type	Width [min]	Area [mAU*s]	Height [mAU]	Area %
1	0.831	BB	0.0252	982.49011	583.14264	97.8345
2	1.154	BB	0.0192	21.74672	15.66750	2.1655
Totals :				1004.23684	598.81014	

Signal 3: DAD1 C, Sig=290,4 Ref=550,10

Peak #	RetTime [min]	Type	Width [min]	Area [mAU*s]	Height [mAU]	Area %
1	0.831	BB	0.0251	916.18665	560.62006	100.0000
Totals :				916.18665	560.62006	

MS Spectrum

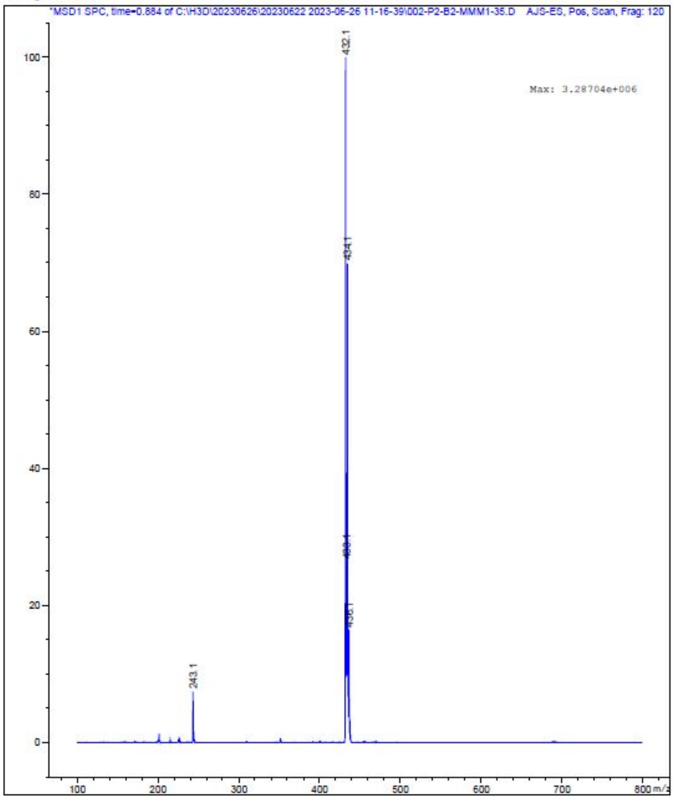

HPLC_UV and MS spectra of **29**

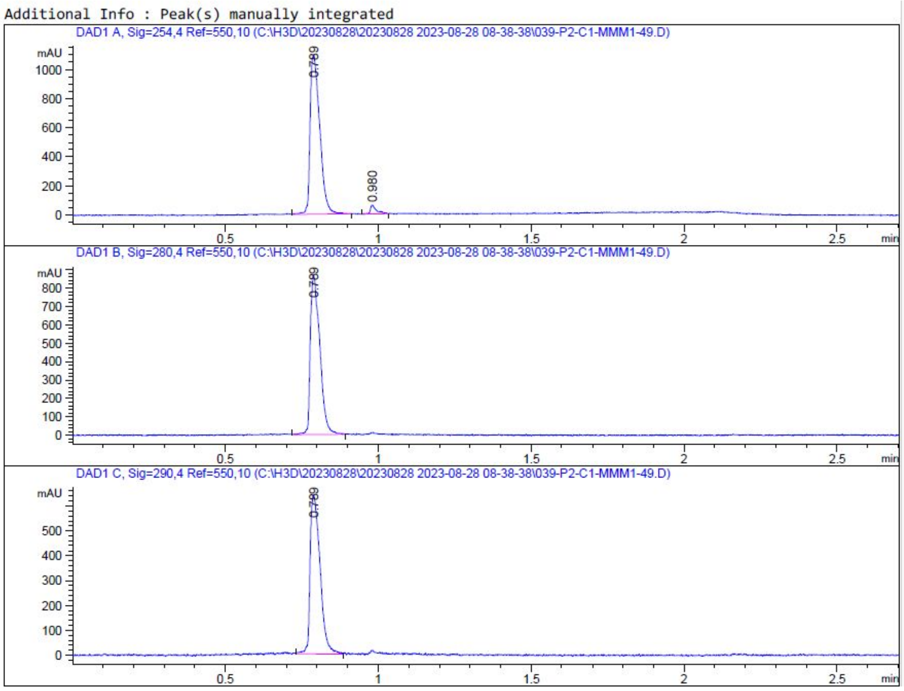

Signal 1: DAD1 A, Sig=254,4 Ref=550,10

Peak #	RetTime [min]	Type	Width [min]	Area [mAU*s]	Height [mAU]	Area %
1	0.789	BB	0.0305	2389.89600	1094.26782	96.3614
2	0.980	BB	0.0194	90.24288	62.29402	3.6386
Totals :				2480.13888	1156.56184	

Signal 2: DAD1 B, Sig=280,4 Ref=550,10

Peak #	RetTime [min]	Type	Width [min]	Area [mAU*s]	Height [mAU]	Area %
1	0.789	BB	0.0306	1856.58972	862.19067	100.0000
Totals :				1856.58972	862.19067	

Signal 3: DAD1 C, Sig=290,4 Ref=550,10

Peak #	RetTime [min]	Type	Width [min]	Area [mAU*s]	Height [mAU]	Area %
1	0.789	BB	0.0323	1441.53613	638.62128	100.0000
Totals :				1441.53613	638.62128	

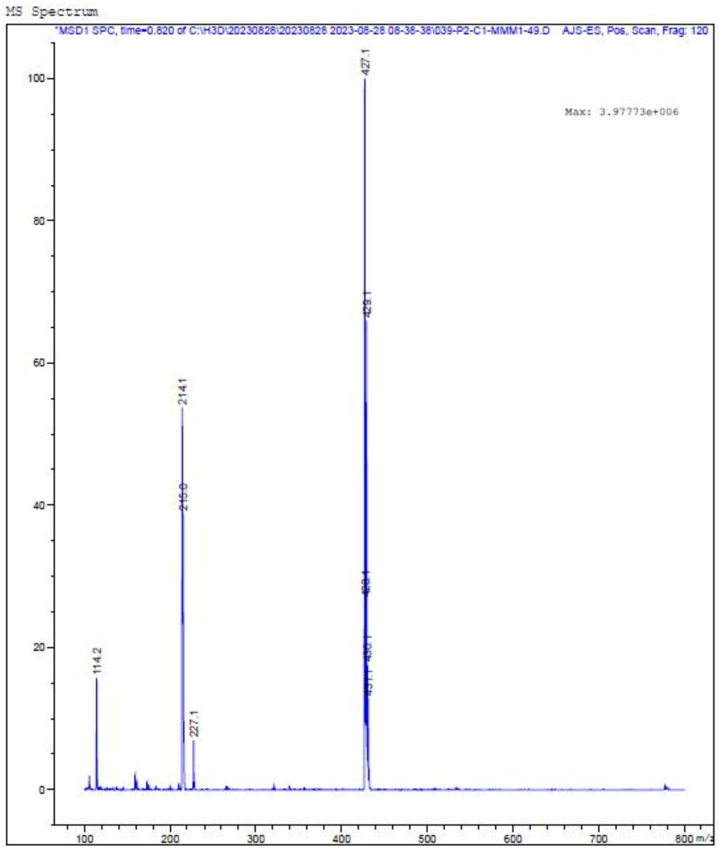

NMR data of final compounds

^{1}H NMR spectrum **GSK-190937 (4)**

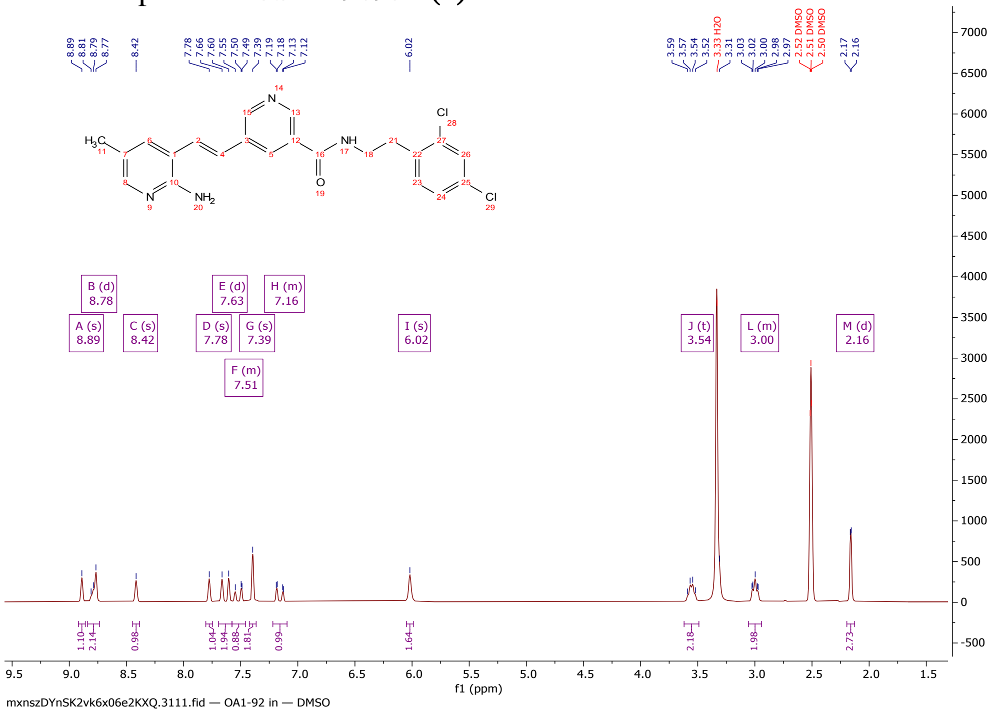

^{1}H NMR spectrum of **5**

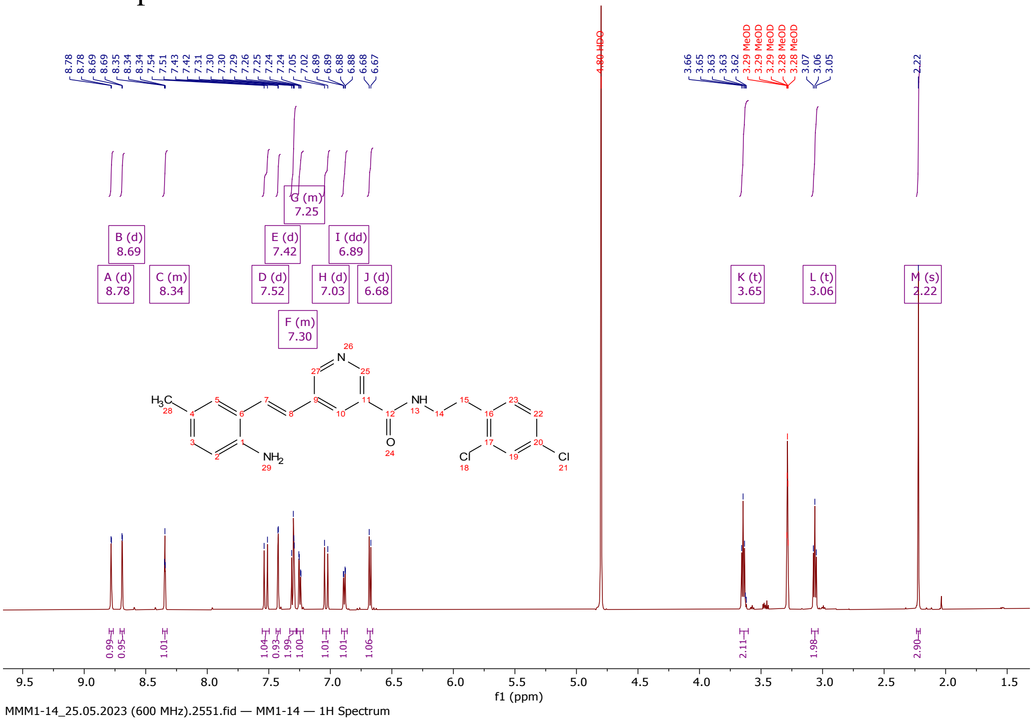

^{1}H NMR spectrum of **6**

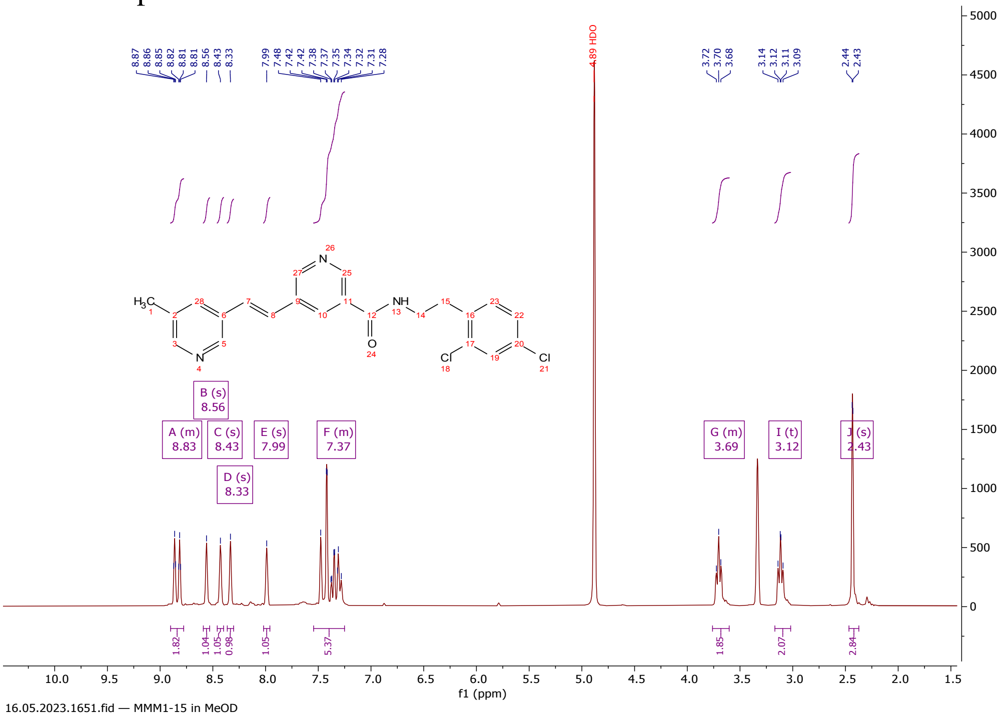

^{1}H NMR spectrum of **7**

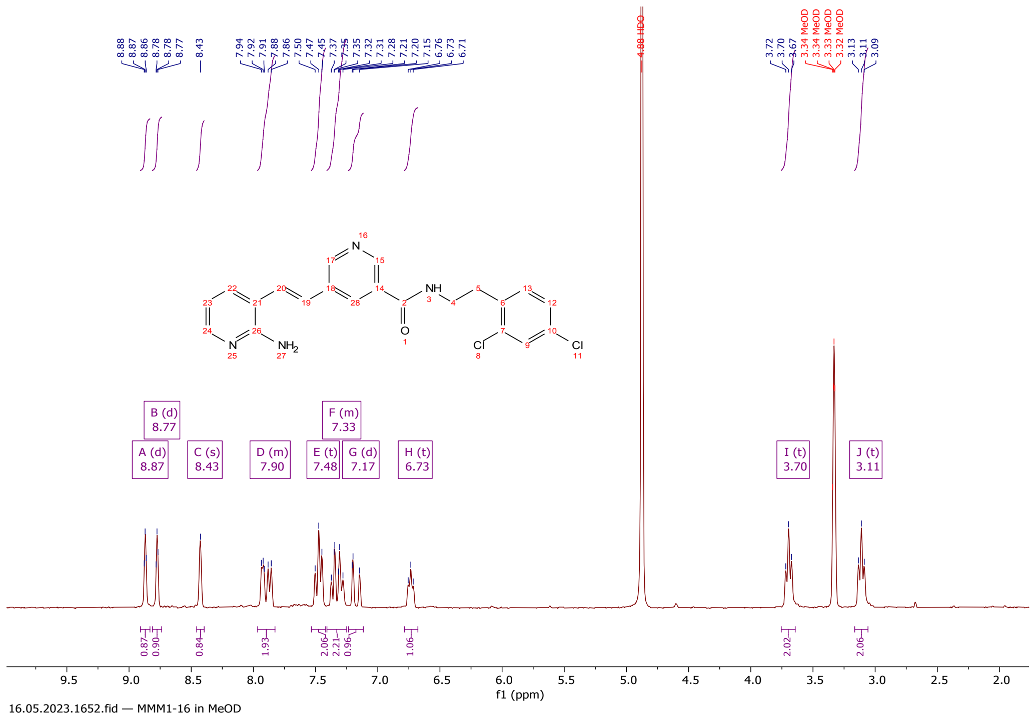

^{1}H NMR spectrum of **8**

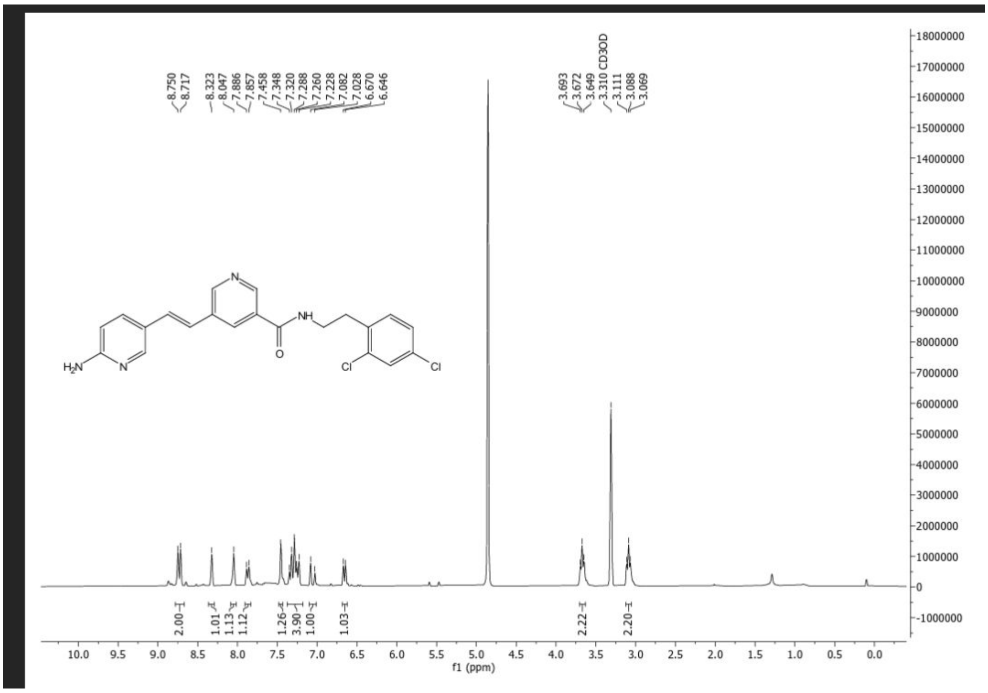

^{1}H NMR spectrum of **9**

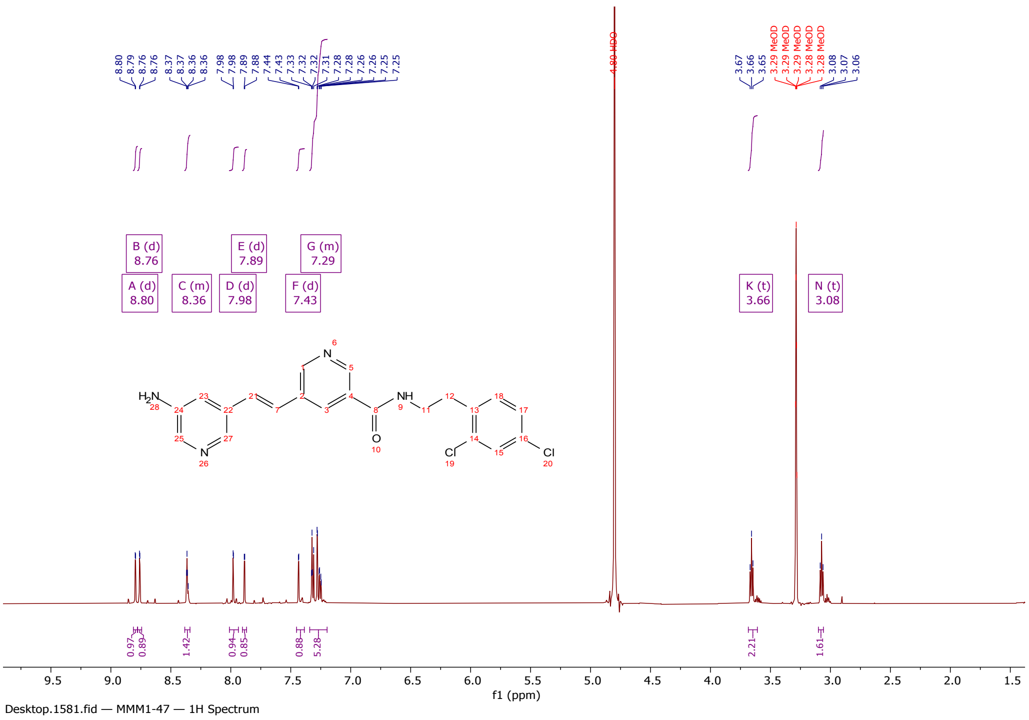

^{1}H NMR spectrum of **10**

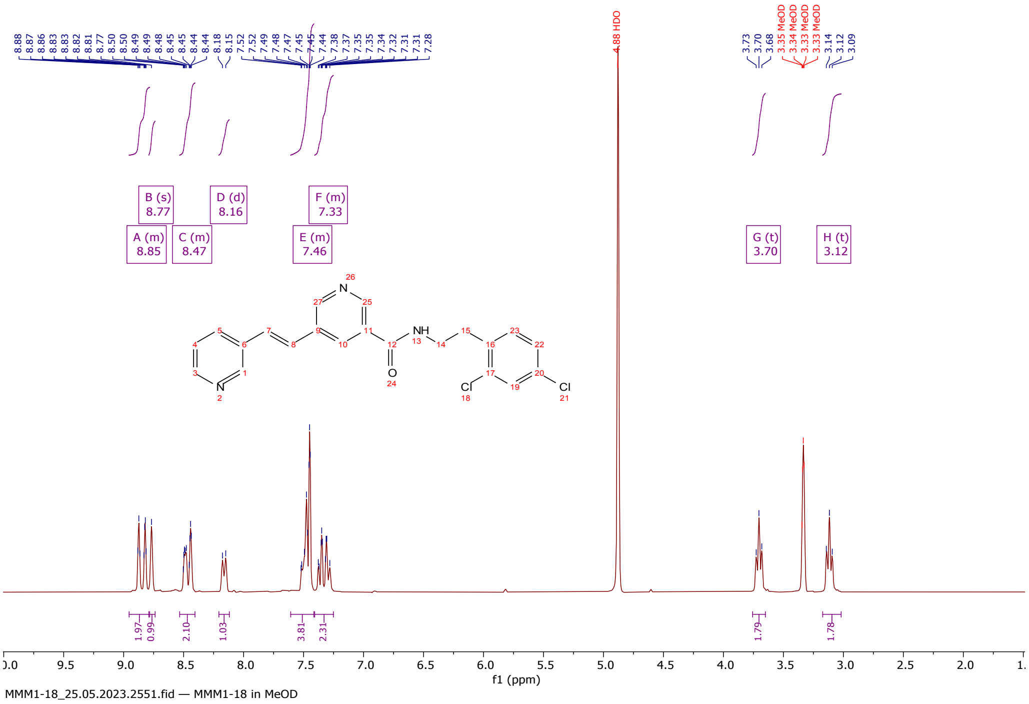

^{1}H NMR spectrum of **11**

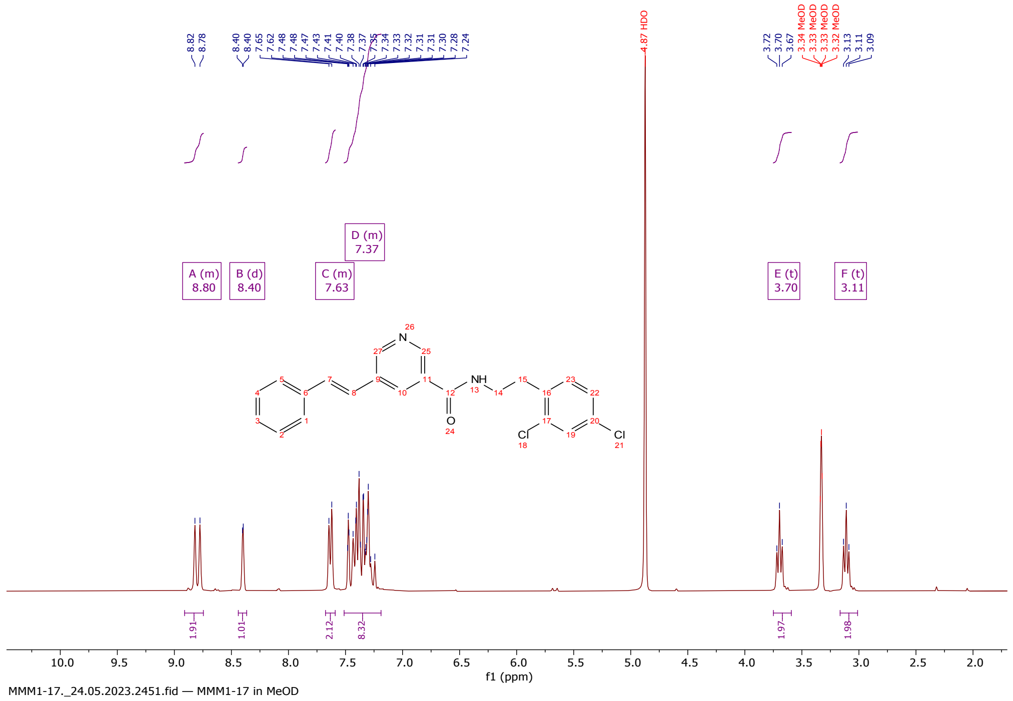

^{1}H NMR spectrum of **12**

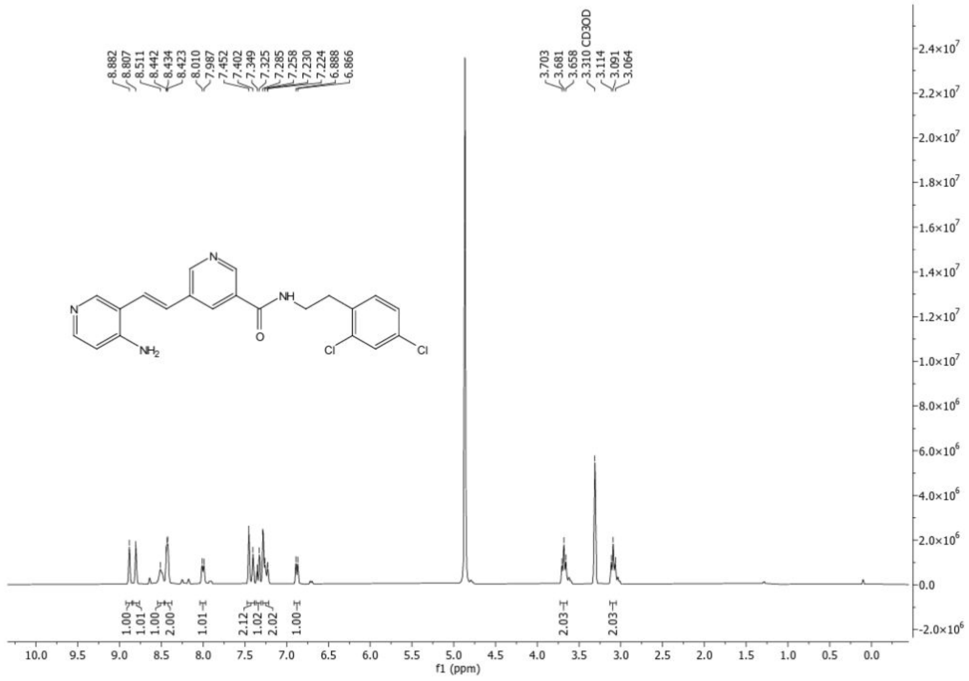

^{1}H NMR spectrum of **13**

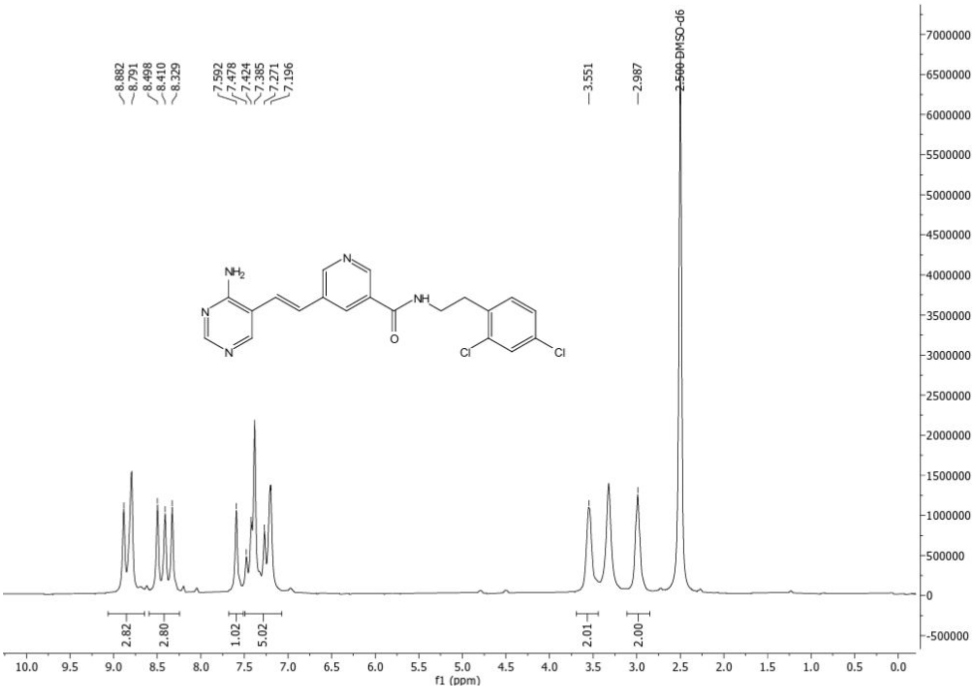

^{1}H NMR spectrum of **17**

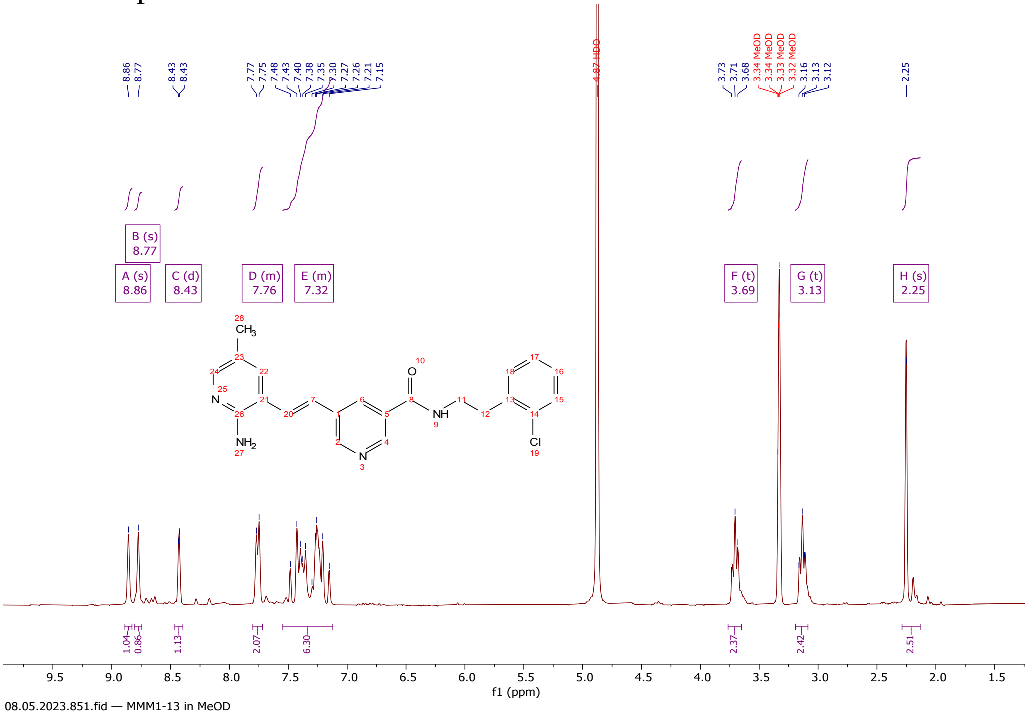

^{1}H NMR spectrum of **18**

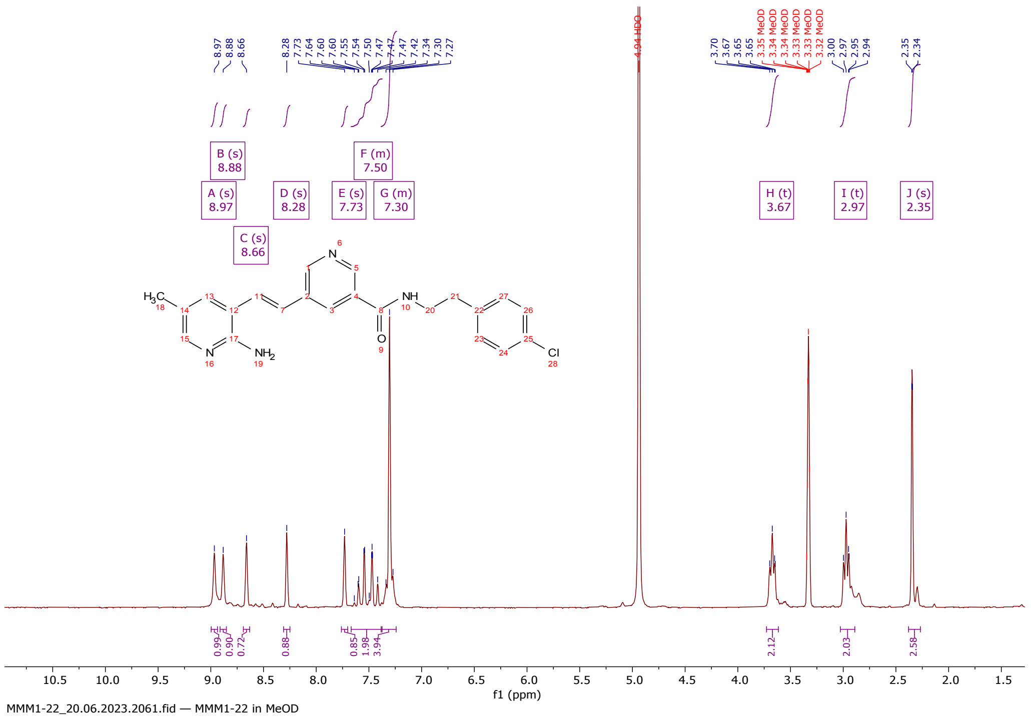

^1H NMR spectrum of **19**

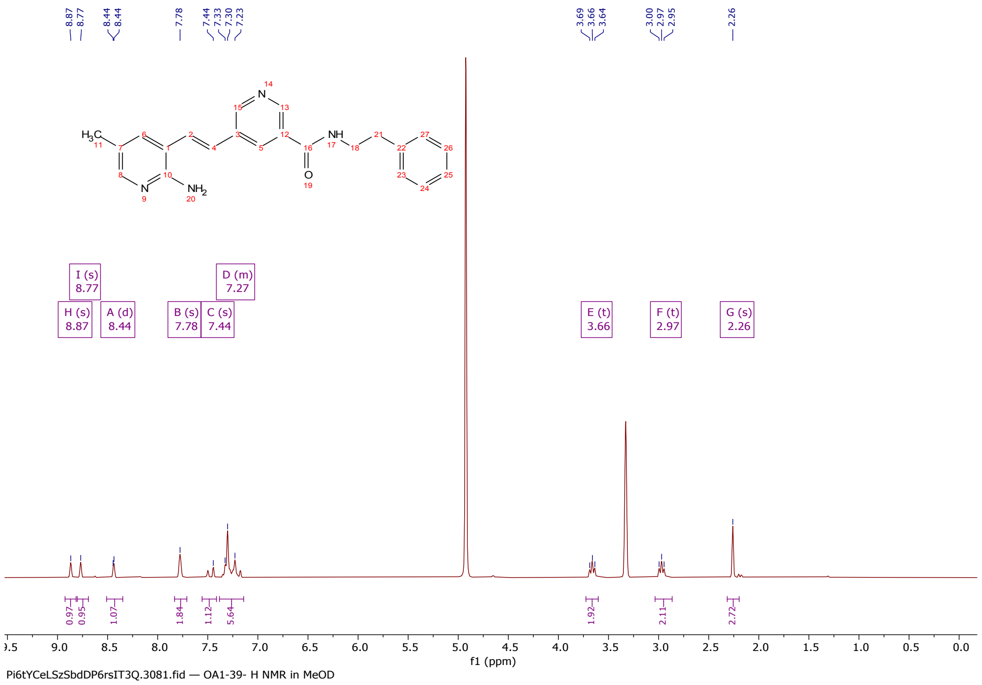

^1H NMR spectrum of **20**

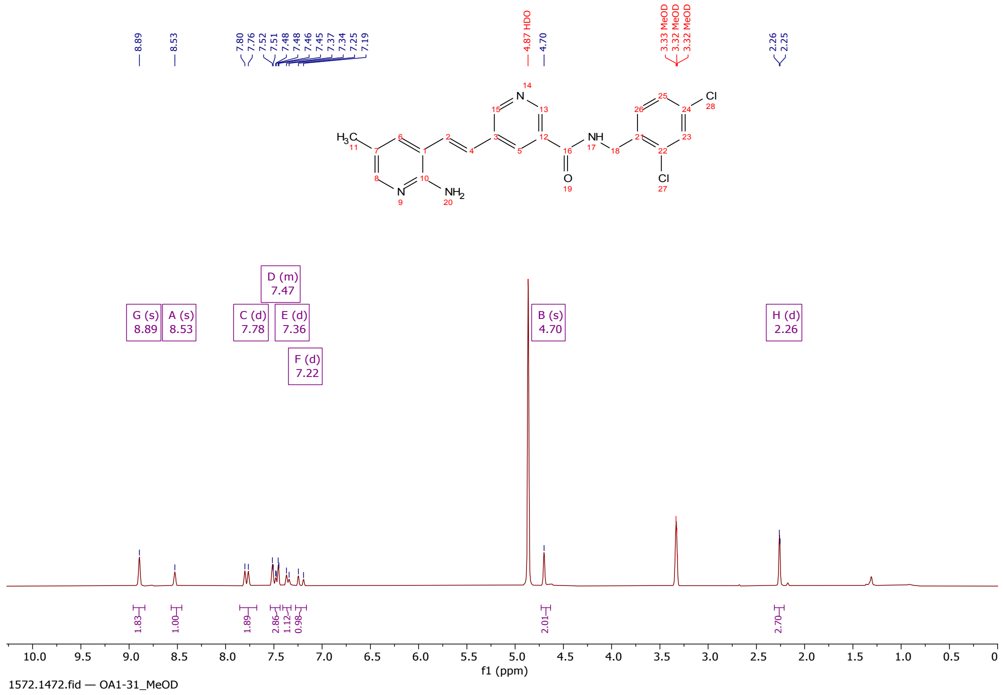

^{13}C NMR spectrum of **20**

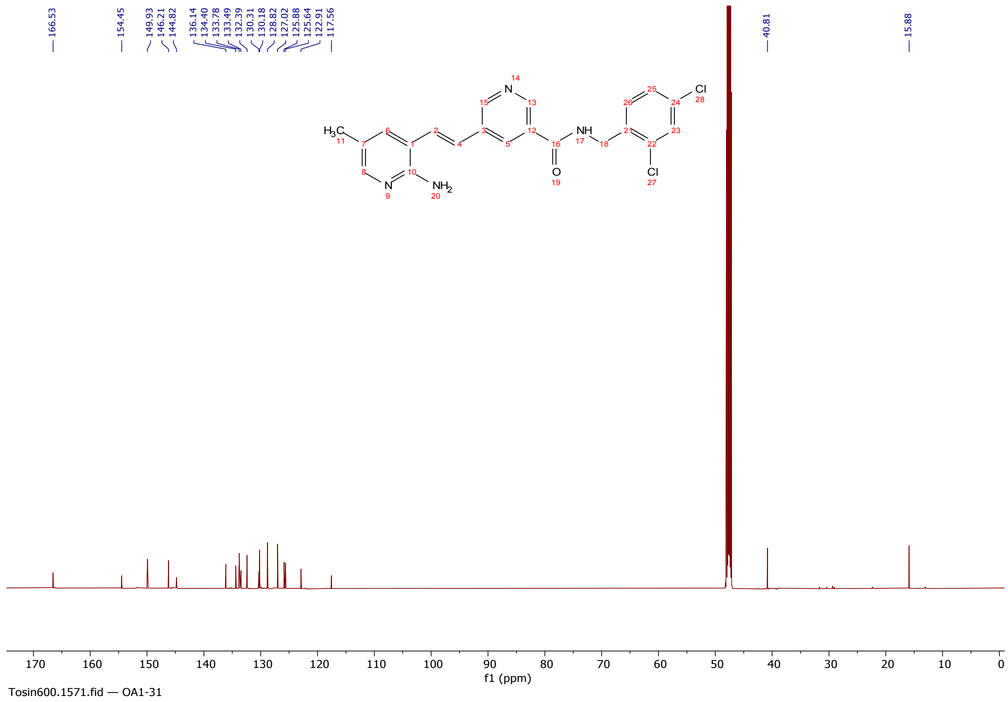

^{1}H NMR spectrum of **21**

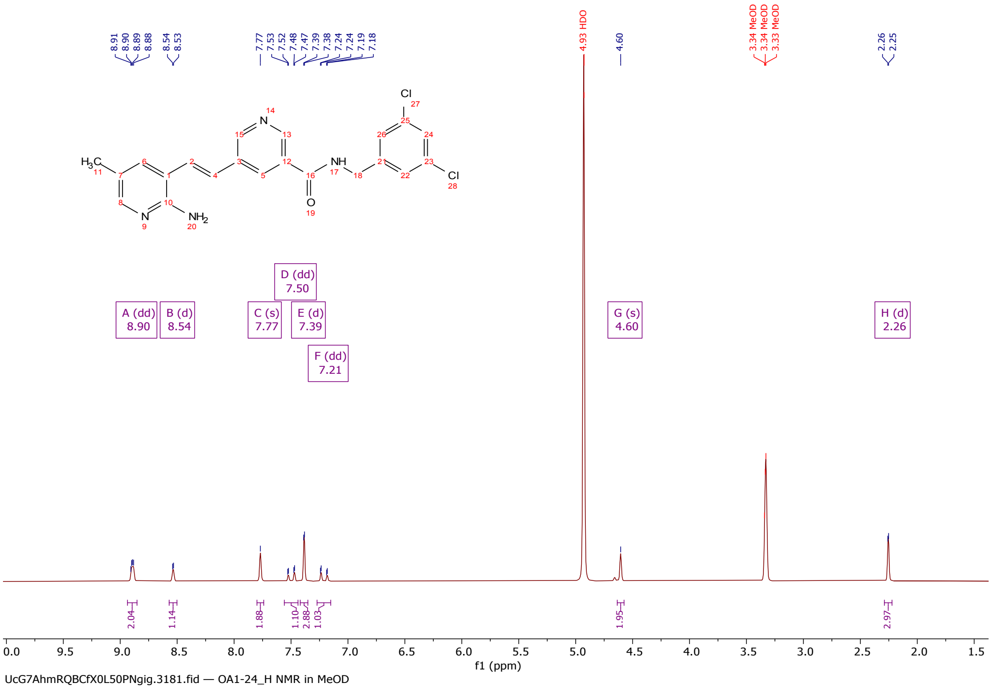

^{1}H NMR spectrum of **22**

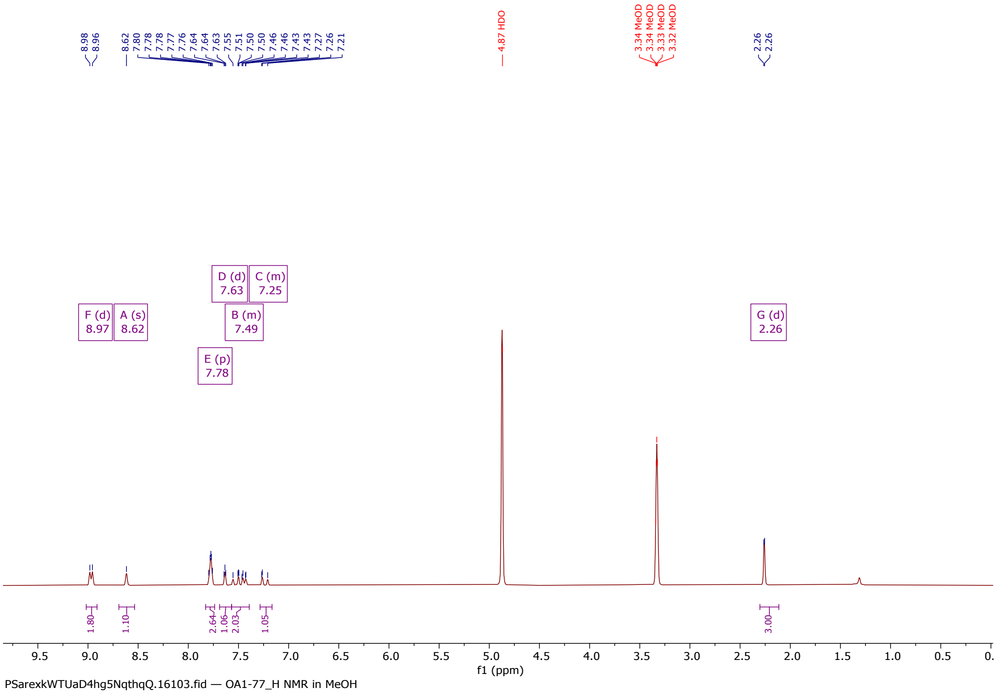

^{1}H NMR spectrum of **23**

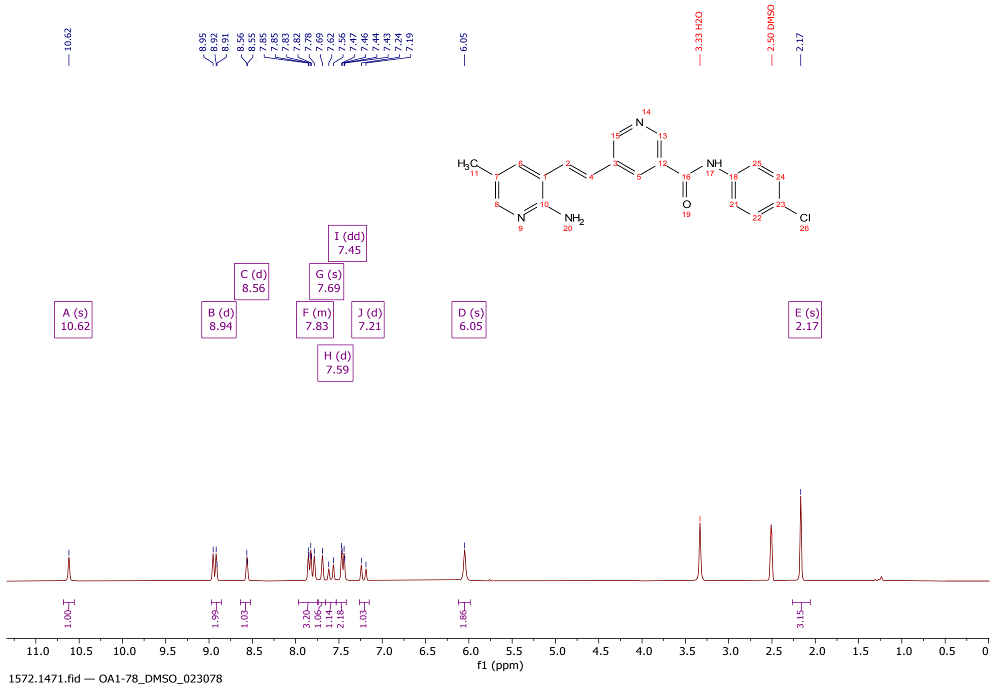

^{13}C NMR spectrum of **23**

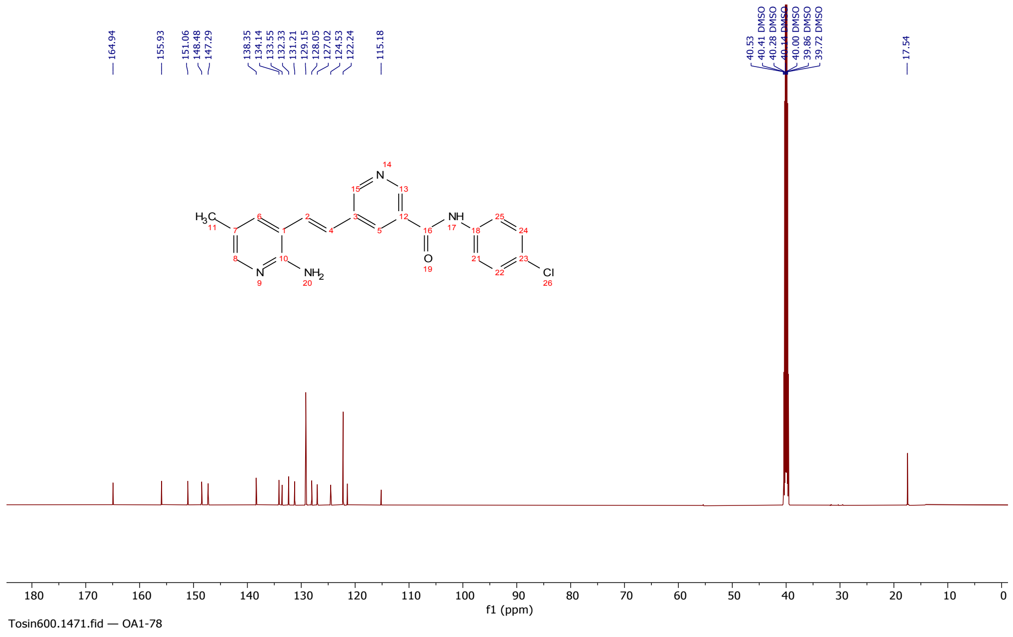

^{1}H NMR spectrum of **24**

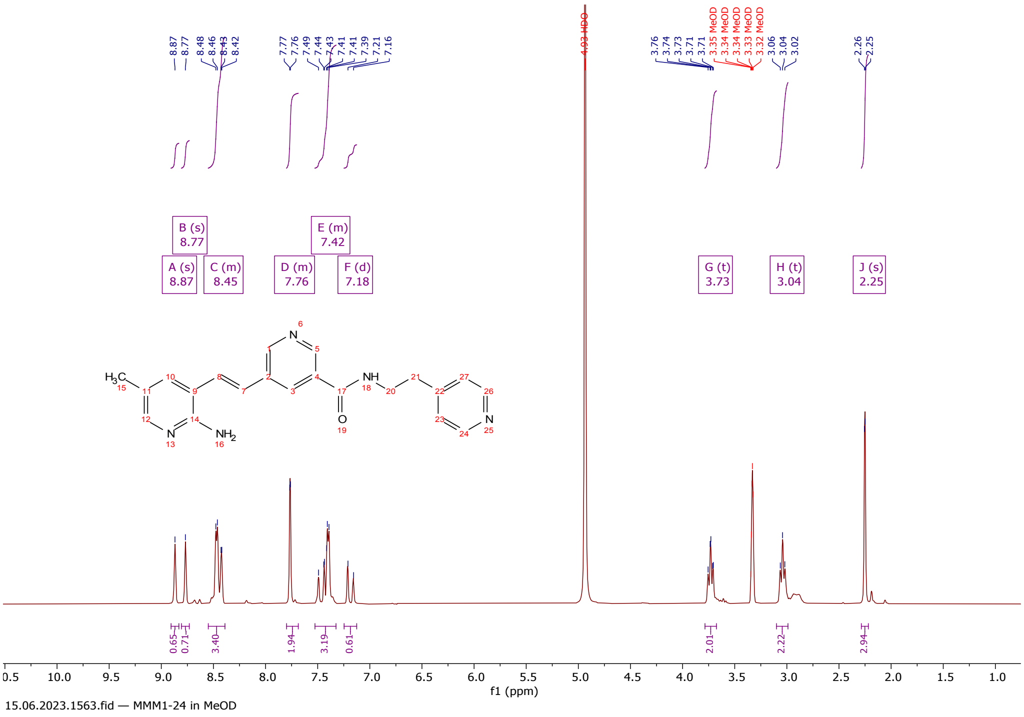

^{1}H NMR spectrum of **25**

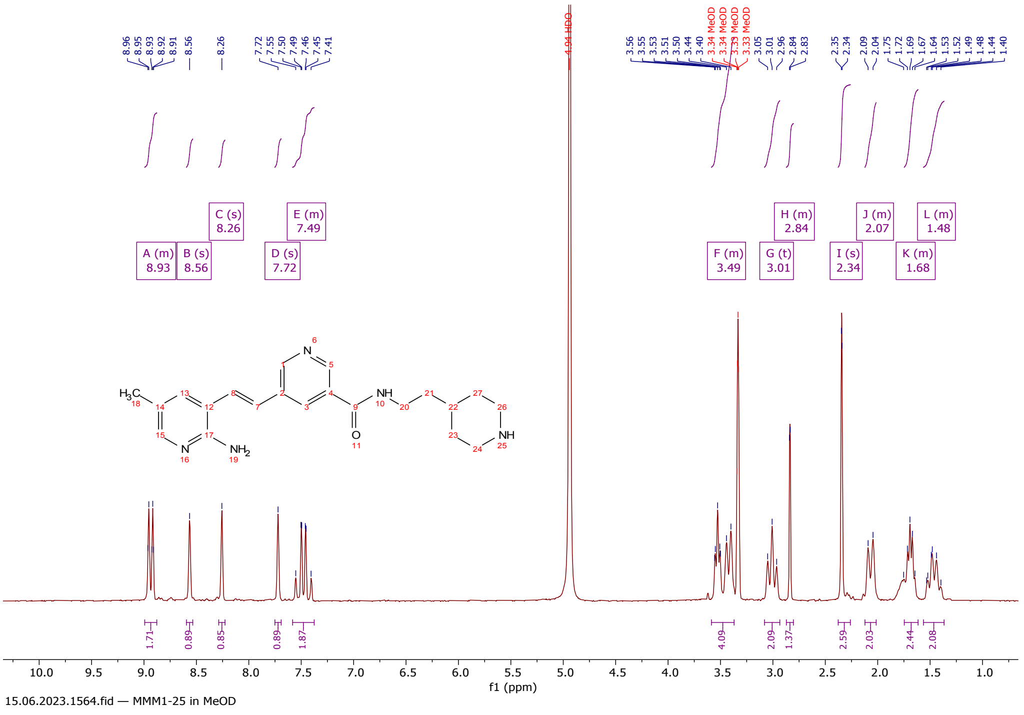

^{1}H NMR spectrum of **26**

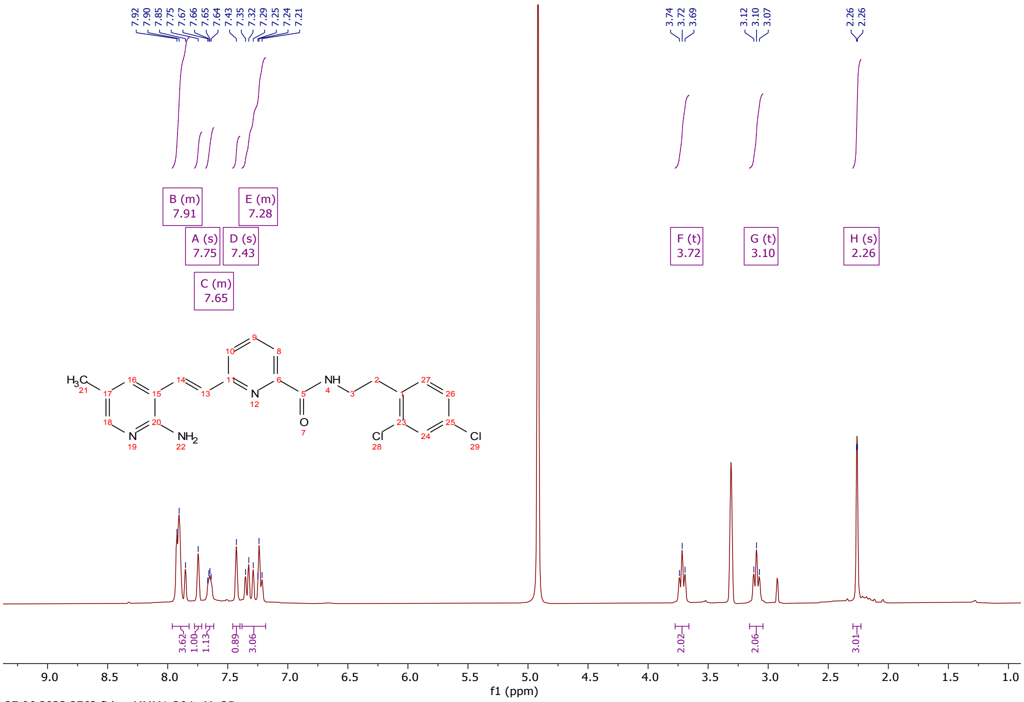

^{1}H NMR spectrum of **27**

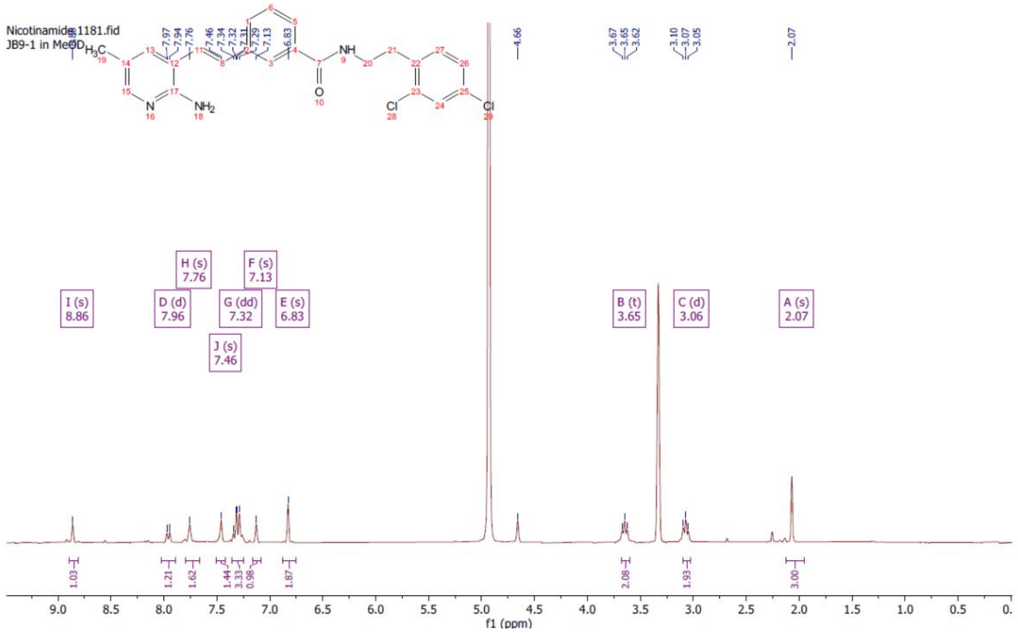

^{1}H NMR spectrum of **28**

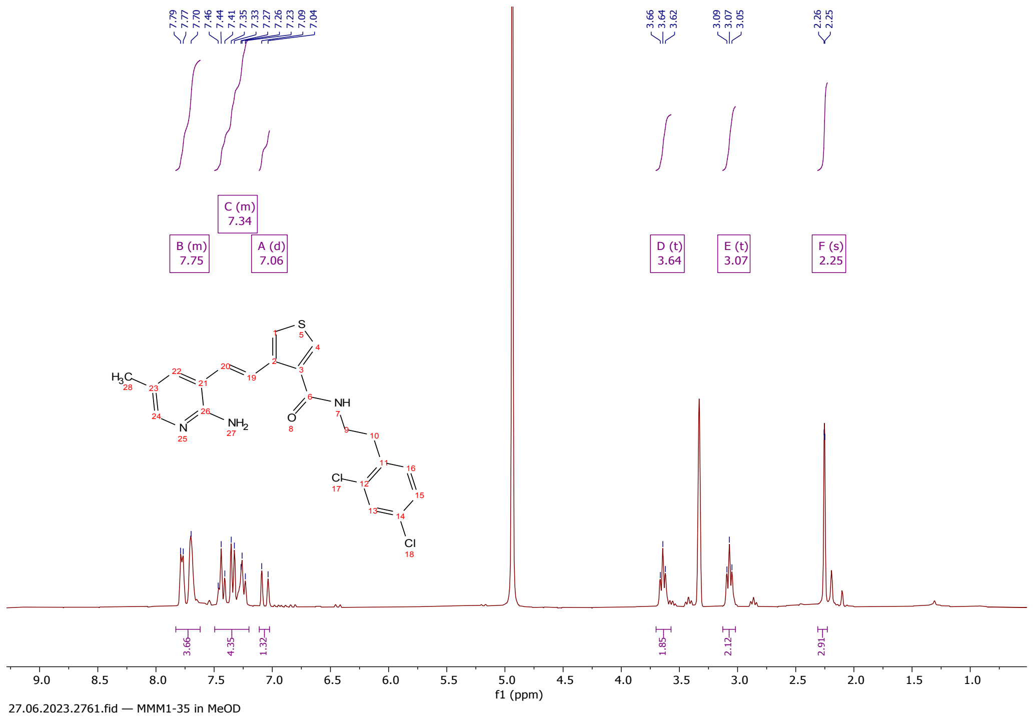

^{1}H NMR spectrum of **29**

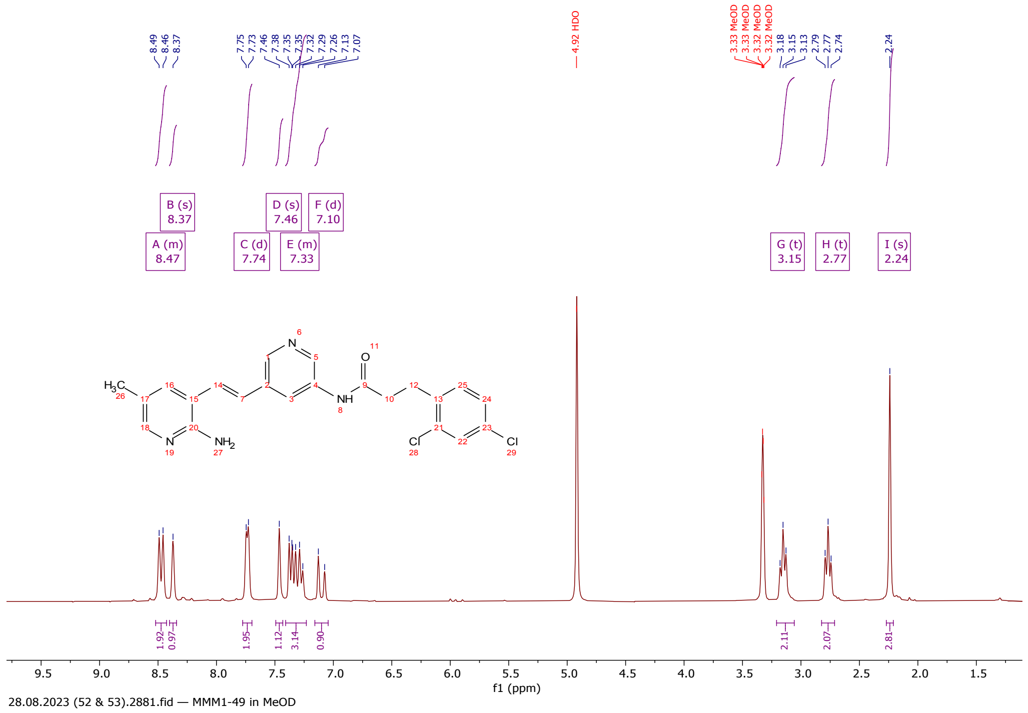

^{13}C NMR spectrum of **29**

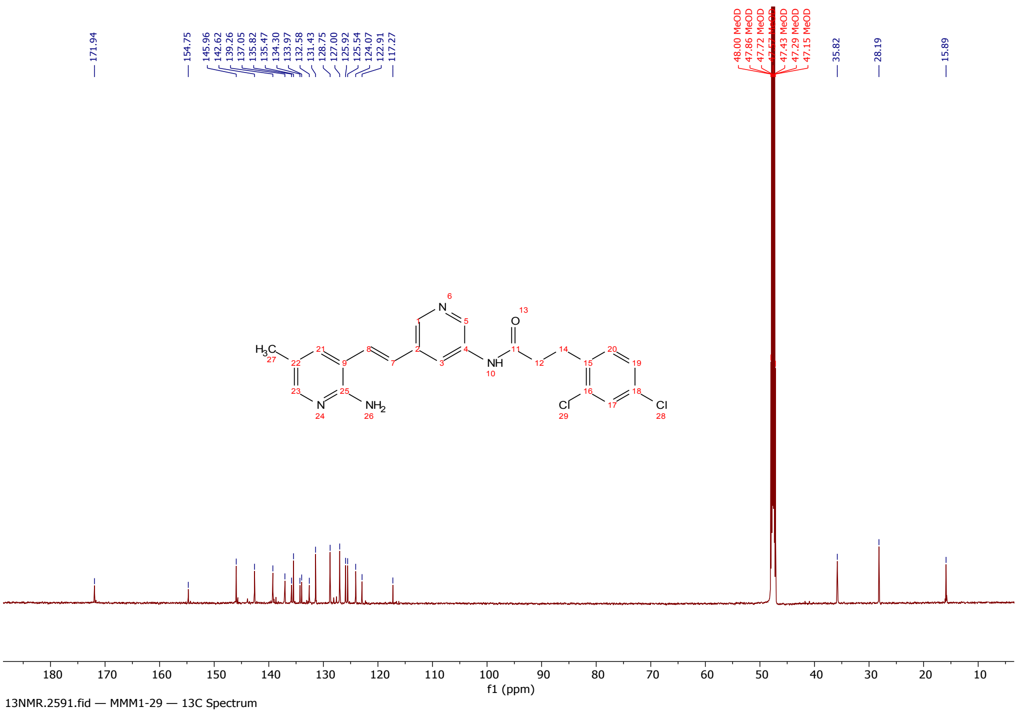

3. Biological and physicochemical assays

In vitro antiplasmodium activity

Test samples were screened for *in vitro* antiplasmodium activity against a chloroquine sensitive (CQS) strain (NF54) and multidrug-resistant (K1 and Dd2) strains of the malaria parasite *P. falciparum*. Continuous *in vitro* cultures of asexual erythrocyte stages of *P. falciparum* were maintained using a modified version of the method of Trager and Jensen (1976).[1] Quantitative assessment of antiplasmodium activity *in vitro* was determined via the parasite lactate dehydrogenase assay using a modified version of the method described by Makler (1993).[2] Samples were tested in triplicate on two separate occasions from 10mM stock solutions in DMSO, from which further dilutions were prepared in complete medium on the day of the experiment. Samples were tested as a suspension if not completely dissolved. A full dose-response was performed from a starting concentration of 6000 nM. Chloroquine and artesunate were used as the reference drugs and were tested from a starting concentration of 1000 ng/mL. The highest concentration of solvent to which the parasites were exposed has no measurable effect on the parasite viability (data not shown). The IC_{50} was generated using non-linear regression analysis via the Dotmatics software platform.

***In vitro* cytotoxicity assays**: Compounds were tested for *in vitro* cytotoxicity against a mammalian cell-line, Chinese Hamster Ovarian (CHO) using the 3-(4,5-dimethylthiazol-2-yl)-2,5- diphenyl-tetrazoliumbromide (MTT)-assay.[3] The samples were tested in triplicate on one occasion from a starting concentration of 50µM as a dose-response evaluation. The test samples were prepared to a 10 mM stock solution in DMSO and were tested as a suspension if not properly dissolved. Emetine was used as the reference drug in all experiments. The highest concentration of solvent to which the cells were exposed to have no measurable effect on the

cell viability. The IC_{50} values were obtained from non-linear curve fitting analysis via the Dotmatics screening platform.

Cell-free β-hematin inhibition assay: The NP-40 detergent-based assay methods for inhibitors of βH formation described by Carter *et al.*[4] and Sandlin *et al.*[5,6] were modified for manual liquid delivery. Samples were dissolved in DMSO to give 10 mM solutions and 20 µL of each was delivered to wells in the last column (column 12) of a 96-well plate together with distilled water (140 µL) and NP40 substitute detergent (305.5 µM, 40 µL). A solution containing water/NP40 substitute (305.5 µM)/DMSO at a v/v ratio of 70%/20%/10% respectively was prepared and then 100 µL was added to all other wells (columns 1-11). A serial dilution of each compound (100 µL) from column 12 down to column 2 was carried out. Column 1 served as a blank with 0 µM sample. A 25 µM hematin stock solution was prepared by sonicating hemin in DMSO for one minute and then suspending 178 µL of this in a 2 M acetate buffer (20 mL, pH 4.8). The homogenous suspension (100 µL) was then added to the wells to give final buffer and hematin concentrations of 1 M and 100 µM respectively. The plate was covered and incubated at 37 ˚C for 5 h. Analysis was carried out using the pyridine-ferrichrome method developed by Ncokazi and Egan.[7] A solution of 50% (v/v) pyridine, 30% (v/v) H_2O, 20% (v/v) acetone and 0.2 M HEPES buffer (pH 7.4) was prepared and 32 µL added to each well to give a final pyridine concentration of 5% (v/v). Acetone (60 µL) was then added to assist with hematin dispersion. The UV-vis absorbance of the plate wells was read on a Thermo Scientific MultiskanGO plate reader. Sigmoidal dose-response curves were fitted to the absorbance data using GraphPad Prism v8 to obtain a 50% inhibitory concentration (IC_{50}) for each compound.

Cellular heme fractionation assay: Before commencing the experiment, the IC_{50} values of each compound was measured in duplicate with a starting parasitaemia of 5% in the 48 h pLDH assay, and this value was used to determine the concentration of test compound in the cell fractionation assay.[8] The 5% parasitaemia represents that used in the cell fractionation assay and accounts for any inoculum effects.

Cell fractionation was performed according to previously published methods, optimized to a multi-well colorimetric assay for determining heme species in *P. falciparum* as described by Combrinck *et al.*[8] The cellular fractionation allows for direct quantification of heme species in isolated trophozoites. The effect on the absolute amounts of heme and hemozoin after treatment at 2 x IC_{50} value of **4** or compound **17**, or 0.5-3x IC_{50} value for **4** were evaluated by measuring the increase in freely exchangeable heme and the decrease in hemozoin species in NF54 parasites in quadruplicate from two independent experiments. Data was plotted and analysed using GraphPad Prism v10.

Solubility: Solubility was performed using an adaptation of the miniaturised shake flask equilibrium solubility method.[9, 10] 10mM stock solutions of the compounds in DMSO were

transferred to 96-well plates using a Hamilton Microlab Starlet automated liquid handler. The DMSO was then dried down under vacuum in a Genevac Mivac Quattro centrifugal concentrator (90min, 37 °C). Thereafter, the samples were reconstituted (200 µM) in aqueous solution and shaken (200rpm, 20 hours, 25 ˚C). The same 10mM stocks were then used to prepare calibration standards (10-220 µM) in DMSO. The solutions were analysed by means of HPLC-DAD (Agilent 1200 Rapid Resolution HPLC with a diode array detector). Solubility was then determined using the peak areas of the aqueous samples and the best fit linear regression curves constructed using the calibration standards.

***In vitro* metabolic stability assay**: The *in vitro* microsomal stability assay was performed in duplicate in a 96-well micro titre plate, using a single-point experiment design.[11] The test compounds (1 µM) were incubated individually in human (pool of 50, mixed gender), rat (pool of 711, male Sprague Dawley) and mouse (pool of 1634, male CD1) liver microsomes (final protein concentration of 0.4 mg/mL; Xenotech, Kansas, USA), suspended in 0.1M phosphate buffer (pH 7.4). Incubations were started by addition of NADPH (1 mM) as cofactor and shaken for 30 minutes at 37 °C. The reactions were then quenched by adding 300 µL of ice-cold acetonitrile containing internal standard (carbamazepine, 0.0236 µg/mL) and centrifuged. The supernatant was transferred to a fresh 96-well plate and analysed by LC-MS/MS (Agilent Rapid Resolution HPLC, AB SCIEX 4500 MS). The relative loss of parent compound over time was monitored and plots (concentration vs. time) were prepared per compound to determine the first order rate constant for compound depletion. This was in turn used to calculate half-life, in vitro intrinsic clearance and in vivo hepatic extraction ratio.[12]

4. References

1. Trager, W.; Jensen, J. B. Human Malaria Parasites in Continuous Culture. *Science*, **1976**, *193*, 673–675.
2. Makler, M.T.; Ries, J.M.; Williams, J.A.; Bancroft, J.E.; Piper, R.C.; Gibbins, B.L.; Hinrichs, D.J. Parasite lactate dehydrogenase as an assay for *Plasmodium falciparum* drug sensitivity. *Am. J. Trop. Med. Hyg.* **1993** Jun;48(6):739-41. doi: 10.4269/ajtmh.1993.48.739. PMID: 8333566.
3. Mosmann, T. Rapid Colorimetric Assay for Cellular Growth and Survival: Application to Proliferation and Cytotoxicity Assays. *J. Immunol. Methods*. **1983**, *65*, 55–63.
4. Carter, M.D.; Phelan, V.V.; Sandlin, R. D.; Bachmann, B.O.; Wright, D.W. Lipophilic Mediated Assays for β-Hematin Inhibitors. *Comb. Chem. High T. Scr.* **2010,** *3*, 285–292.
5. Sandlin, R.D.; Carter, M.D.; Lee, P.J.; Auschwitz, J.M.; Leed, S.E.; Johnson, J.D.; Wright, D. W. Use of the NP-40 Detergent-Mediated Assay in Discovery of Inhibitors of Beta-Hematin Crystallization. *Antimicrob. Agents Chemother.* **2011,** *55*, 3363–3369.
6. Sandlin, R.D.; Fong, K.Y.; Wicht, K.J.; Carrell, H.M.; Egan, T.J.; Wright, D.W. Identification of beta-Hematin Inhibitors in a High-Throughput Screening Effort Reveals Scaffolds with *in vitro* Antimalarial Activity. *Int. J. Parasitol.* **2014,** *4*, 316–325.
7. Ncokazi, K.K.; Egan, T.J. A colorimetric high-throughput b-hematin inhibition screening assay for use in the search for antimalarial compounds. *Anal. Biochem.* **2005,** *338*, 306–319.
8. Combrinck, J.M.; Fong, K.Y.; Gibhard, L.; Smith, P.J.; Wright, D.W.; Egan, T.J. Optimization of a multi-well colorimetric assay to determine haem species in *Plasmodium falciparum* in the presence of anti-malarials. *Malar. J.* **2015** Jun 24;14:253. doi: 10.1186/s12936-015-0729-9. PMID: 26099266; PMCID: PMC4484700.
9. Alelyunas, Y. W.; Liu, R.; Pelosi-Kilby, L.; Shen, C. Application of a Dried-DMSO Rapid Throughput 24-h Equilibrium Solubility in Advancing Discovery Candidates. *Eur. J. Pharm. Sci.* **2009**, *37*, 172–182.
10. Zhou, L.; Yang, L.; Tilton, S.; Wang, J. Development of a High Throughput Equilibrium Solubility Assay Using Miniaturized Shake-Flask Method in Early Drug Discovery. **2007**, *96* (11), 3052–3071. https://doi.org/10.1002/jps.
11. Di, L.; Kerns, E. H.; Gao, N.; Li, S. Q.; Huang, Y.; Bourassa, J. L.; Huryn, D. M. Experimental Design on Single-Time-Point High-Throughput Microsomal Stability Assay. *J. Pharm. Sci.* **2004**, *93* (6), 1537–1544. https://doi.org/10.1002/jps.20076.
12. Obach, R. S. Prediction of Human Clearance of Twenty-Nine Drugs from Hepatic Microsomal Intrinsic Clearance Data: An Examination of in Vitro Half-Life Approach and Nonspecific Binding to Microsomes. *Drug Metab. Dispos.* **1999**, *27*, 1350–1359.
